# ENIGMA’s simple seven: Recommendations to enhance the reproducibility of resting-state fMRI in traumatic brain injury

**DOI:** 10.1016/j.nicl.2024.103585

**Published:** 2024-03-05

**Authors:** Karen Caeyenberghs, Phoebe Imms, Andrei Irimia, Martin M. Monti, Carrie Esopenko, Nicola L. de Souza, Juan F. Dominguez D, Mary R. Newsome, Ekaterina Dobryakova, Andrew Cwiek, Hollie A.C. Mullin, Nicholas J. Kim, Andrew R. Mayer, Maheen M. Adamson, Kevin Bickart, Katherine M. Breedlove, Emily L. Dennis, Seth G. Disner, Courtney Haswell, Cooper B. Hodges, Kristen R. Hoskinson, Paula K. Johnson, Marsh Königs, Lucia M. Li, Spencer W. Liebel, Abigail Livny, Rajendra A. Morey, Alexandra M. Muir, Alexander Olsen, Adeel Razi, Matthew Su, David F. Tate, Carmen Velez, Elisabeth A. Wilde, Brandon A. Zielinski, Paul M. Thompson, Frank G. Hillary

**Affiliations:** a Cognitive Neuroscience Unit, School of Psychology, Deakin University, Geelong, Australia; b Ethel Percy Andrus Gerontology Center, Leonard Davis School of Gerontology, University of Southern California, Los Angeles, CA, USA; c Alfred E. Mann Department of Biomedical Engineering, Andrew & Erna Viterbi School of Engineering, University of Southern California, Los Angeles, CA, USA; d Department of Quantitative & Computational Biology, Dana and David Dornsife College of Arts & Sciences, University of Southern California, Los Angeles, CA, USA; e Department of Psychology, UCLA, USA; f Brain Injury Research Center (BIRC), Department of Neurosurgery, UCLA, USA; g Department of Rehabilitation and Human Performance, Icahn School of Medicine at Mount Sinai, NY, USA; h Michael E. DeBakey VA Medical Center, Houston, TX, USA; i H. Ben Taub Department of Physical Medicine and Rehabilitation, Baylor College of Medicine, Houston, TX, USA; j TBI and Concussion Center, Department of Neurology, University of Utah, Salt Lake City, UT, USA; k Center for Traumatic Brain Injury, Kessler Foundation, East Hanover, NJ, USA; l Rutgers New Jersey Medical School, Newark, NJ, USA; m Department of Psychology, Penn State University, State College, PA, USA; n Women's Operational Military Exposure Network (WOMEN) & Rehabilitation Department, VA Palo Alto, Palo Alto, CA, USA; o Rehabilitation Service, VA Palo Alto, Palo Alto, CA, USA; p Neurosurgery, Stanford School of Medicine, Stanford, CA, USA; q UCLA Steve Tisch BrainSPORT Program, USA; r Department of Neurology, David Geffen School of Medicine at UCLA, USA; s Center for Clinical Spectroscopy, Brigham and Women’s Hospital, Boston, MA, USA; t Department of Radiology, Harvard Medical School, Boston, MA, USA; u George E. Wahlen Veterans Affairs Medical Center, Salt Lake City, UT, USA; v Minneapolis VA Health Care System, Minneapolis, MN, USA; w Department of Psychiatry and Behavioral Sciences, University of Minnesota Medical School, Minneapolis, MN, USA; x Department of Psychiatry and Behavioral Sciences, Duke University, Durham, NC, USA; y Department of Psychology, Brigham Young University, Provo, UT, USA; z Center for Biobehavioral Health, The Abigail Wexner Research Institute at Nationwide Children's Hospital, Columbus, OH, USA; aa Department of Pediatrics, The Ohio State University College of Medicine, OH, USA; ab Neuroscience Center, Brigham Young University, Provo, UT, USA; ac Emma Children's Hospital, Amsterdam UMC, University of Amsterdam, Emma Neuroscience Group, The Netherlands; ad Amsterdam Reproduction and Development, Amsterdam, The Netherlands; ae C3NL, Imperial College London, United Kingdom; af UK DRI Centre for Health Care and Technology, Imperial College London, United Kingdom; ag Division of Diagnostic Imaging, Sheba Medical Center, Tel-Hashomer, Israel; ah Sagol School of Neuroscience, Tel-Aviv University, Tel-Aviv, Israel; ai Sackler Faculty of Medicine, Tel-Aviv University, Tel-Aviv, Israel; aj Mind Research Network, Albuquerque, NM, USA; ak Departments of Neurology and Psychiatry, University of New Mexico School of Medicine, Albuquerque, NM, USA; al Duke-UNC Brain Imaging and Analysis Center, Duke University, Durham, NC, USA; am VA Mid-Atlantic Mental Illness Research Education and Clinical Center, Durham, NC, USA; an Department of Psychology, Norwegian University of Science and Technology, Trondheim, Norway; ao Clinic of Rehabilitation, St. Olavs Hospital, Trondheim University Hospital, Trondheim, Norway; ap NorHEAD – Norwegian Centre for Headache Research, Norwegian University of Science and Technology, Trondheim, Norway; aq Turner Institute for Brain and Mental Health, School of Psychological Sciences, Monash University, Clayton, VIC 3800, Australia; ar Wellcome Centre for Human Neuroimaging, University College London, WC1N 3AR London, United Kingdom; as CIFAR Azrieli Global Scholars Program, CIFAR, Toronto, ON, Canada; at Departments of Pediatrics, Neurology, and Neuroscience, University of Florida, Gainesville, FL, USA; au Departments of Pediatrics, Neurology, and Radiology, University of Utah, Salt Lake City, UT, USA; av Imaging Genetics Center, Mark and Mary Stevens Neuroimaging & Informatics Institute, University of Southern California, Marina del Rey, CA, USA; aw Department of Neurology, Hershey Medical Center, PA, USA

**Keywords:** Traumatic brain injury, Functional MRI, Resting state fMRI, Lesions, Functional connectivity, Reproducibility

## Abstract

Resting state functional magnetic resonance imaging (rsfMRI) provides researchers and clinicians with a powerful tool to examine functional connectivity across large-scale brain networks, with ever-increasing applications to the study of neurological disorders, such as traumatic brain injury (TBI). While rsfMRI holds unparalleled promise in systems neurosciences, its acquisition and analytical methodology across research groups is variable, resulting in a literature that is challenging to integrate and interpret. The focus of this narrative review is to address the primary methodological issues including investigator decision points in the application of rsfMRI to study the consequences of TBI. As part of the ENIGMA Brain Injury working group, we have collaborated to identify a minimum set of recommendations that are designed to produce results that are reliable, harmonizable, and reproducible for the TBI imaging research community. Part one of this review provides the results of a literature search of current rsfMRI studies of TBI, highlighting key design considerations and data processing pipelines. Part two outlines seven data acquisition, processing, and analysis recommendations with the goal of maximizing study reliability and between-site comparability, while preserving investigator autonomy. Part three summarizes new directions and opportunities for future rsfMRI studies in TBI patients. The goal is to galvanize the TBI community to gain consensus for a set of rigorous and reproducible methods, and to increase analytical transparency and data sharing to address the reproducibility crisis in the field.

## Highlights

• rsfMRI has seen increased adoption recently, with 181 published studies to date.
• 7 recommendations to produce results that are reliable, harmonizable and reproducible.
• Future directions include connectomics, lesion mapping and single-subject analyses.

## 1 Introduction

Resting-state functional magnetic resonance imaging (rsfMRI) has provided neuroscientists with a powerful tool to model synchronous low-frequency oscillations in the blood oxygen level dependent (BOLD) signal as an apparent measure of brain activity patterns (Biswal et al., 1995, Lee et al., 2013). Resting-state fMRI eliminates task demands and typically shortens scan times, making it easier to use with cognitively impaired participants who may have difficulty sustaining attention or completing complex fMRI tasks while in the scanner (e.g., Leunissen et al., 2013). In the first application of rsfMRI to brain injury, Nakamura and colleagues (2009) performed whole-brain network analyses of the rsfMRI signal in 8 patients at 3 months and 6 months following severe TBI. Their results revealed alterations of network properties including enhanced functional connectivity (FC) early after injury that became more comparable to healthy adults over the course of the first 6 months post injury. Since this initial study, more than 100 rsfMRI studies have been published in TBI patients with a particular surge in contributions in the most recent years. The scope, methodology, and utility of rsfMRI in TBI has been reviewed in previous review papers (e.g., Caeyenberghs et al., 2017, Hayes et al., 2016, Morelli et al., 2021, O’Neill et al., 2017, Esagoff et al., 2023). For example, Hayes and colleagues (2016) summarized findings from both structural, diffusion, and rsfMRI studies to examine how brain networks are disrupted by axonal injury. In another review, Caeyenberghs and colleagues (2017) reviewed alterations to graph theoretical properties of the functional brain networks in TBI patients, outlining pertinent methodological challenges associated with the examination of FC in these patients.

Despite the breadth of empirical work examining rsfMRI changes after TBI, the literature has yet to coalesce around a consistent set of findings. Efforts for meaningful *meta*-analyses have been unsuccessful (Bickart et al., 2023, Eierud et al., 2014; Hallquist and Hillary, 2018; Hannawi et al., 2015, Verhulst et al., 2023) and were at least partially the inspiration for this critical review. For example, Bickart and colleagues (2023) recently concluded that there were no consistent findings in TBI and rsfMRI. Another *meta*-analysis by Verhulst and colleagues (2023) demonstrated that FC within the default-mode network showed consistent associations with cognitive performance in all types of acute onset brain injury and was associated with worse cognition. However, this consistent finding was only found in four studies in TBI and stroke patients. Inconsistent results across studies pose critical challenges to the field and hinder progress towards clinical and diagnostic applications of rsfMRI. With the effects of the replication crisis now observable in virtually every discipline in science, there is increasing concern regarding the reproducibility of TBI research (Priestley et al., 2023).

The methods used in rsfMRI have not been spared from this scrutiny, with several reviews outlining the challenges of reproducibility in the rsfMRI literature in both basic (Esteban et al., 2019, Poldrack et al., 2017) and applied neurosciences (Hallquist and Hillary, 2018). There has been growing concern about the reliability of rsfMRI methods and how they might be appropriately applied to the study of TBI across basic (cognitive neuroscience) and applied (neurorehabilitation) topics. For example, in a review of 106 rsfMRI studies in the clinical neurosciences (including TBI), more than 50 brain parcellation schemes were utilized to create networks for analysis, rendering the literature nearly impossible to integrate and interpret (Hallquist and Hillary, 2018). The wide range of pathways for data analysis results in a decentralized implementation of rsfMRI procedures, with low methodological overlap, poor harmonizability, and conflicting interpretations. For example, when 70 investigators were asked to test the same hypotheses on the same imaging data set, no two teams chose an identical analysis workflow: thus, the investigators’ results were highly discrepant (Botvinik-Nezer et al., 2020). Concerns about researcher degrees of freedom (Gelman and Loken, 2013) and under-powered studies (Button et al., 2019) are now being voiced, refocusing attention on maximizing reliability in rsfMRI studies (Teeuw et al., 2021). Unfortunately, the problem of “forking paths” (which refers to investigator freedom to choose from myriad analytical approaches) that Gelman and Loken (2013) discuss, appears trivial when compared to the massive parameter space facing investigators using rsfMRI. When considering only the *pre-processing* of data, Poldrack and colleagues (2017) show that there are more than 60,000 pathways to analyze data using accepted procedures. There is an urgent need to reconcile inconsistent findings in rsfMRI studies (see Hallquist and Hillary, 2018; Rajtmajer et al., 2022), a seemingly impossible task in the face of methodological divergence of this magnitude.

This reproducibility crisis is more pronounced when studying patients with TBI due to the heterogeneity in brain injuries. TBI can result from different causes (such as traffic accidents, falls, assaults, blast explosions), leading to diverse lesion types, locations and sizes across TBI patients (Covington and Duff, 2021). This variability in lesion characteristics can result in inconsistencies in resting state networks and other diverse patterns of FC across patients, making it challenging to identify reproducible findings across rsfMRI studies in TBI patients (Bickart et al., 2023). There is also a high between-subject variability in brain reorganization mechanisms (i.e., differences in restoration and compensation mechanism) across TBI patients, which can lead to differences in FC patterns (Hylin et al., 2017). Patients with TBI can present with a wide range of cognitive and motor deficits, potentially increasing head motion during fMRI scans (Caeyenberghs et al., 2009; Irimia & Van Horn, 2015). This can introduce artifacts and spurious correlations in the data and reduce the quality of the rsfMRI data, impacting the reproducibility of results. The majority of rsMRI studies in TBI patients have low sample sizes with insufficient statistical power, impacting the generalizability. There are several strategies that TBI researchers can employ to enhance reproducibility of rsfMRI studies, which will help improve the reliability and generalizability of findings in this challenging population.

**Fig. 1:**
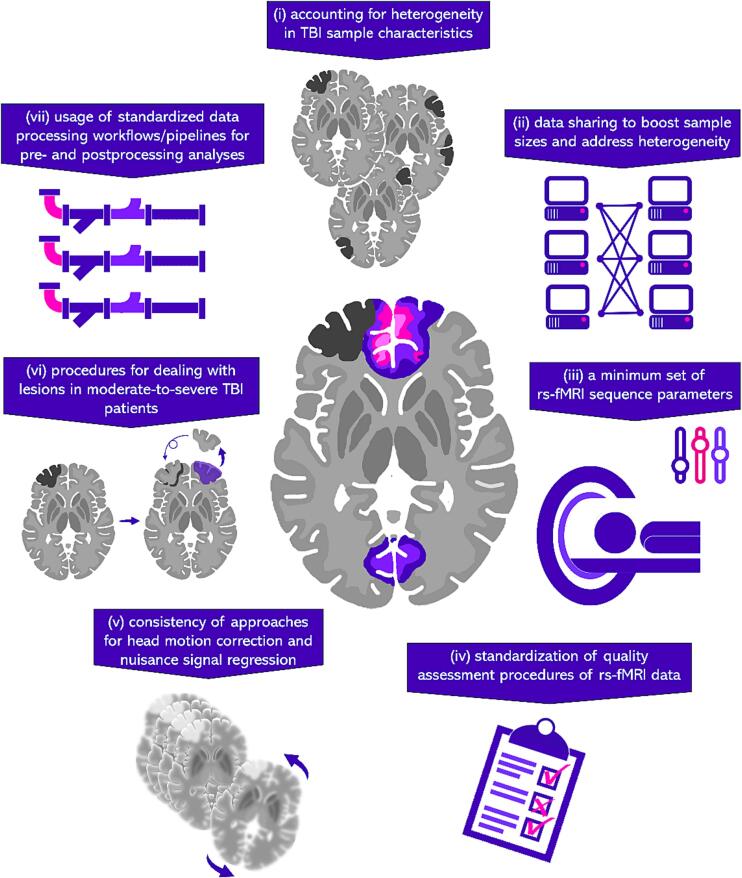
Schematic overview of the seven recommendations from the ENIGMA rsfMRI working group.

One approach to address issues of reproducibility is to identify a minimum set of recommended methods that are reliable, harmonizable, and reproducible within the TBI imaging research community. *The primary focus of this review is to galvanize the TBI research community in favor of an agreed-upon set of reproducible methods.* We aim to increase analytical transparency and data sharing to address long-standing methodological challenges in the field, while preserving investigators’ freedom to choose methods appropriate to their empirical questions. To achieve this, we will outline the following seven recommendations (see Fig. 1 for an overview) to improve the reproducibility of rsfMRI studies in TBI patients: (i) account for heterogeneity in TBI sample characteristics; (ii) share data to boost sample sizes, increase representativeness, and address heterogeneity; (iii) utilize a minimum set of rsfMRI sequence acquisition parameters; (iv) standardize quality assessment procedures of rsfMRI data; (v) employ consistent approaches for head motion correction and nuisance signal regression; (vi) standardize procedures for dealing with lesions in moderate-to-severe TBI patients; and (vii) use standardized data processing workflows for pre- and post-processing analyses. These seven recommendations should serve as a useful reference for those TBI researchers who are new to rsfMRI analysis, helping them to avoid ‘analysis paralysis’ in the face of tens of thousands of highly technical methodological decisions, and as a check for those who may already be running studies, but may have overlooked some important confounds. In part 1, we provide an overview of 181 currently published rsfMRI studies in TBI, focusing on their design choices and processing techniques. In part 2, we offer a set of recommendations for best practices, supported by key literature. In cases where no solutions are obvious, we highlight and chart important goals for their future resolution. Finally, in part 3, we outline new directions and opportunities for future rsfMRI studies in TBI patients, including multimodal MRI studies, lesion mapping strategies, and single-subject analyses.

## 2 Part 1: Summary of the studies

For this narrative review, we aimed to understand the methods used in rsfMRI studies of TBI, so a basic search strategy was conducted using Ovid Medline to retrieve rsfMRI studies in TBI patients. The search strategy used alternative terms for TBI (“traumatic brain injur*” OR “TBI” OR “moderate to severe TBI” OR “severe TBI” or “concussion”) AND rsfMRI (“rsfMRI” or “functional connect*” OR “functional network” OR “connect*” OR “graph theory”). The search strategy was further refined by limiting the results to studies carried out in human populations and excluding review papers. After this initial search, duplicates were manually removed, and remaining results were screened and reviewed in the full-text format for relevance and quality. Because the primary goal was to understand investigators' methods/approaches in this literature, inclusion of studies was also based on the co-authors’ expertise and familiarity with the rsfMRI literature. One hundred eighty-one research articles (153 in mild and 28 in moderate-to-severe TBI) met the search criteria.

To critique the methods used in this literature, we documented the key study parameters of each study (such as sample size, acquisition parameters, pre-and post-processing techniques) in the Supplementary Table. This Table provide an overview of the methodological issues that can affect the reliability of rsfMRI results in TBI patients. These results were used to guide our set of recommendations to improve the reproducibility of rsfMRI studies, which are outlined in section 3.

In the 181 studies reviewed in this paper, different approaches were used to examine resting-state brain activity in TBI patients (see Supplementary Table). The majority of the studies (86 studies) have used a region-of-interest (ROI) analysis, whereby a seed region is selected a priori, and the subsequent FC map is extracted from the temporal correlations between the ROI and all other brain regions. Fifty-six studies applied network analyses or graph theory on the resting-state fMRI data. Thirty-six papers have employed the independent component analysis (ICA) method, whereby the data for the entire brain is decomposed into a set number of components, each of which is depicted as a functional map. Only a few ICA studies evaluated resting-state FC differences of the same canonical network, including the default mode network (6 studies), task-positive network (2 studies), salience network (2 studies), fronto-parietal network (2 studies). Twelve studies extracted voxel-wise quantitative features from the rsfMRI data, such as the regional homogeneity (ReHo, 3 studies), in TBI patients. The remaining studies used either one of these methods in combination with other analysis techniques, or a completely different approach, such as principal component analysis (PCA, 4 studies), multi-voxel pattern analysis (MPVA, 2 studies), Bayesian multi-subject vector autoregressive modelling (BVAR, 1 study), and cross-correlational analysis (CCA, 1 study). This variability in the analysis characteristics varied so greatly that it was impossible for us to perform a *meta*-analysis.

## 3 Part 2: Recommendations

### 3.1 Account for heterogeneity in sample characteristics

It is critical to account for heterogeneity in patients with TBI when attempting to categorize injury-based rsfMRI connectivity alterations. More so than in other brain disorders, TBI patients present with a wide range of injury characteristics and individual factors that can result in resting state functional connectivity (rsFC) fluctuations (Priestley et al., 2023). However, 57.46% of studies in our review had sample sizes less than 30 (see Supplementary Table), resulting in conclusions that may not be generalizable within and across different populations. Focused attention is required to address how myriad effects ranging from demographics to clinical injury variables influence modeling and ultimately the reproducibility of rsfMRI results. A total of 7, 392 participants were studied across all papers included in the review. Mild TBI, as opposed to moderate-severe TBI, accounts for over 90% of the population studied.

Males commonly show higher rates of TBI (Frost et al., 2013) and have greater representation in the studied population (68.87%). Perhaps due to less representation of women, 90.06% of studies did not report including sex as a covariate in post-processing analyses. Sex remains a critical variable of study with studies now showing that females have higher rates of sports-related concussion in sex comparable sports (Bretzin et al., 2021, Covassin et al., 2016, O'Connor et al., 2017). Because of this, there is growing emphasis in examining sex differences in both the biological response to injury as well as in the study of long-term outcome. As one example, epidemiological data suggest sex differences in recovery trajectories for both sports-related (Bretzin et al., 2022) and non-sports related (Roby et al., 2023) concussions. Moreover, studies have demonstrated sex differences in cognitive functioning for both acute (Broshek et al., 2005, Iverson et al., 2017) and chronic TBI stages (Forslund et al., 2019, Levin et al., 2021, Rauen et al., 2021). Biological older adult males are more likely to show decreases in rsFC six months after mild TBI, compared to similar age females (Amgalan et al., 2022a). Others have pointed to sexual dimorphism of brain morphometry including axonal structure as a source of between-sex difference in symptom presentation and recovery following TBI (Dolle et al., 2018). While this literature continues to emerge, there is ample evidence that investigating the biological basis for recovery of TBI requires consideration of biological sex, and work to date has only begun to do so.

With respect to age, the majority of studies in our review (70.59%) recruited mostly adult TBI patients (> 21 years). TBI rates increase with age (Peters and Gardner, 2018), and older adults are at greater risk of hospitalization after TBI (Coronado et al., 2005). Older age at injury is a risk factor for significant rsFC alterations, which underlie common post-traumatic cognitive deficits (Amgalan et al., 2022b). Physiologically and anatomically, brain injury during development is vastly different to that during adulthood (Figaji, 2017), making extrapolation of results between age groups inappropriate. Thus, studies should either examine TBI patients according to constrained age ranges, or age should be included as a covariate in all analyses.

Of the reviewed studies, ethnicity and/or race were not reported as covariates in any post-processing analyses. Ethnicity is known to affect TBI risk, treatment, and functional outcomes. In addition to having greater risk of sustaining a TBI (Brenner et al., 2020, Chen et al., 2020), Black and Hispanic patients are less likely to receive treatment post-TBI (Gary et al., 2009) and to be discharged to rehabilitation (Asemota et al., 2013, McQuistion et al., 2016, Budnick et al., 2017, Brenner et al., 2020) relative to White patients. Representation of non-White persons with TBI in the literature is a critical issue as well as the lack of representation of premorbid psychiatric history, homelessness, and substance abuse even though those groups are over-represented in the population of head injury as it naturally occurs (see Dell et al., 2021a, Dell et al., 2021b) These longstanding issues with regard to representativeness must be addressed for generalizability of results; social and biological determinants of health have clear influence on TBI outcome and should be directly modeled.

Time post injury is a critical determinant of system-level plasticity, yet it was modeled in the analysis of only 4.42% of studies. Time since injury is a critical factor to consider in understanding recovery trajectories and has been shown to be vital in the understanding of network plasticity. There is evidence that rsFC connectomics may change over time during the first year post injury, with increases in rsFC found at 6-months, 1-year, and 18-months after injury in adult patients with mild, moderate, and severe TBI (Hillary et al., 2011, Sharp et al., 2011, Hillary et al., 2014, Dall’Acqua, P., Johannes, S., Mica, L., Simmen, H. P., Glaab, R., Fandino, J., & Hänggi, J., 2017, de Souza et al., 2020; Roy et al., 2018). Other studies have shown limited changes in rsFC between 3 and 5 months after injury (Mayer et al., 2011), as well as heterogeneous patterns of altered whole-brain connectivity at the chronic stage (Palacios et al., 2013, Venkatesan et al., 2015).

Accounting for important demographic and clinical differences such as injury severity, sex, age, ethnicity, and time since injury when analyzing rsFC after TBI is crucial. Where possible, these variables could be included as covariates in rsfMRI models to understand their influence on network metrics. There have been recent calls to redefine our science with focus on better isolating and defining features that comprise our samples (Boukrina et al., 2020). In other areas of the behavioral sciences there has been a call for a “heterogeneity revolution” (Bryan et al., 2021) in order to address contextual differences in groups – an issue critical to the understanding of TBI and interventions designed to improve outcome. Reliable rsfMRI findings will require that the next generation of studies can define their TBI samples using Common Data Elements (CDEs) or standardized assessments for assessing demographic and clinical characteristics of the TBI patients. For example, a combination of CDEs is commonly utilized to assess TBI severity (Tenovuo et al., 2021), including injury-related measures (such as the Glasgow Coma Scale (GCS) scores, duration of Posttraumatic Amnesia (PTA) assessed using tools such as the Galveston Orientation and Amnesia Test or Westmead PTA scale), clinical assessments (using standardized tools, such as the Injury Severity Score or Abbreviated Injury Score), imaging findings using CT or MRI scans at the time of injury (such as number of lesions, type of lesions, midline shift), or biomarkers (such as levels of axonal markers). Standardizing the collection of these data elements will enhance the comparability of TBI assessments across rs-fMRI studies and facilitate data-sharing (as discussed in the next section). It will contribute to the development of comprehensive databases for advancing TBI research and enable the conduct of *meta*-analyses.

### 3.2 Share data to boost sample sizes, increase representativeness, and address heterogeneity

The rsfMRI TBI literature reviewed here is composed primarily of samples that are heterogeneous with respect to demographics and injury characteristics. Functional imaging studies are uniformly under-powered (see Button, 2019, Poldrack et al., 2017). Our Supplementary Table reveal consistently small sample sizes (< 30, an arbitrarily chosen cut-off to suggest low sample size) across the reviewed papers. The reasons for these shortfalls are many, including challenges with participant enrollment, and in longitudinal studies, retainment, MRI contraindications, and financial expense. Data sharing holds new opportunities to address long-standing problems with under-powered studies and heterogeneity in demographic and clinical factors in TBI that have undoubted influence on patient outcome. Opportunities through team science and data sharing now make this possible (Thompson et al., 2020). For example, the Enhancing Neuro Imaging Genetics through Meta-Analysis (ENIGMA) Brain Injury working group (e.g., Dennis et al., 2021, Dennis et al., 2022, Olsen et al., 2021) has addressed the inconsistent neuroimaging findings in TBI by aggregating MRI data from TBI patients across multiple sites to obtain sufficient power to reliably conduct analyses on MRI data. As one example, Bruin and colleagues (2023) have recently conducted a mega-analysis of rsfMRI data from 1, 024 obsessive compulsive disorder (OCD) patients and 1, 028 healthy controls from 28 independent samples of the ENIGMA-OCD consortium. Their images were analyzed using HALFpipe (Harmonized AnaLysis of Functional MRI pipeline) (Waller et al., 2022), which is an fMRIPrep-based analytical tool modified for the clinical neurosciences (Esteban et al., 2019). HALFPipe follows standardized protocols to standardize data processing, increase data fidelity, and harmonize data across multiple sites (see Adhikari et al., 2019). Their mega-analysis revealed several rsFC differences between people with OCD and healthy matched controls, showing global hypo-connectivity across networks, most prominently in the sensorimotor network, as well as fewer hyper-connections, mostly pertaining to the thalamus. A similar coordinated analysis of rsfMRI data of TBI patients across multiple cohorts of the ENIGMA TBI working group with standardized pipelines is ongoing and may help to address issues of sample heterogeneity and provide novel insights into system-level plasticity and convergence in findings.

A fundamental concern for data sharing and collaborative science is the need to protect participants’ privacy. Researchers need to employ techniques like anonymization, removing personally identifiable information, and implementing strict access controls (see critical reviews, Poline et al., 2012, White et al., 2022). Additionally, establishing data-sharing agreements and obtaining informed consent with clear privacy provisions are crucial steps in safeguarding participants’ confidentiality. To anonymize rsfMRI data investigators should consider applying de-identification software (e.g., Conquest DICOM software, DICOM library, DICOMworks) to automatically scrub *meta*-data from the DICOM data, to ensure that the sensitive information is eliminated (Aryanto et al., 2015). Investigators should use MR defacing algorithms (such as afni_refacer, deepdefacer, mri_deface, mridefacer, etc) to remove facial features, preserving anonymity of the participants (Theyers et al., 2021). Structural anonymizing (defacing) is standard for BIDS format as well. The use of *meta*-data or aggregating data to a higher level (e.g., group averages) can minimize the risk of re-identifications. For example, HALFpipe enables users to tackle consortium analyses of multi‐cohort fMRI data by running a standardized analysis protocol (preprocessing and feature extraction) at each site and/or cohort prior to sharing the derived maps and summary statistics to perform group level statistics (Waller et al., 2022).

### 3.3 Utilize a minimum set of rsfMRI acquisition parameters

The Supplementary Table displays the variability in sequence parameters for rsfMRI data acquisition across the reviewed studies. Resting-state fMRI acquisition parameters depend on several factors, including scanner hardware (field strength, type of radiofrequency coils, gradient strength) and software, and studies’ research questions. However, scanning parameters must be carefully considered and optimized to obtain reliable and reproducible rsfMRI data in TBI patients. In multisite TBI studies, MRI physicists can approximate sequence parameters for different scanners. Also, scanning parameters should be consistently reported: many important parameters, including the resting state condition (eyes closed 41%, eyes open 18%, eyes open with fixation point 13%), were not reported in 40% of the publications (Supplementary Table). While a single set of parameters is unlikely to meet the goals for all investigators, we propose a range of acceptable and literature-driven acquisition parameters (in alignment with the scanning protocol of the Human Connectome Project, see Harms et al., 2018) that should be considered when conducting resting state fMRI studies in TBI patients, such as the field strength, scan duration, spatial resolution, and repetition time. We refer the interested reader to a review paper by Raimondo and colleagues (2021), which provides an overview of recommendations for rsfMRI acquisition strategies for a range of applications, from the most common approaches for rsfMRI acquisition strategies, to more recent rsfMRI studies with dedicated scanner hardware and ultra-high field scanners.

In our review, the majority of the rsfMRI studies were conducted on 3T scanners (approximately 95%; see Supplementary Table). Only 8 studies out of 181 employed 1.5T scanners (and no studies were performed on ultra-high field scanners, such as 7T). Even though high-field MRI scanners (3T or more) increase the risk of susceptibility artifacts, they are preferred for rsfMRI studies as they provide better signal-to-noise ratio (SNR) and a higher spatial resolution (Wardlaw et al., 2012). The scan duration should be long enough to capture the resting-state fluctuations in the BOLD signal, but short enough to avoid fatigue in the participants (Birn et al., 2013, Power et al., 2014, Raimondo et al., 2021). Resting-state fMRI scans typically lasted between 4.1 and 20 min (mean: 7.19 min). However, TBI researchers should consider a minimum of 13 min (for group-based analyses; Birn et al., 2013) and a minimum of 25 min (for single-subject analyses, Laumann et al., 2015; see also section 4.3) to improve the reliability of the rsfMRI scan in TBI patients. This length of the scan duration increases the risk of head motion. Therefore, it is important to consider to minimise head motion through the following steps: (i) advanced motion correction procedures (see section 3.5); (ii) practice sessions in a mock scanner before the actual scanning session if available and (iii) administer two runs (6 min 30 each) of rsfMRI; and (iv) employ multiband acceleration factors (e.g., a multiband factor between 2 and 4) to acquire multiple slices simultaneously (Preibisch et al., 2015, Risk et al., 2021).

As can be seen in the Supplementary Table, repetition time (TR) ranged between 460 and 6565 ms (mean 2041 ms). We recommend a TR ∼ 1 s as a minimum in future studies (e.g., Fan et al., 2021; Gilbert et al., 2018; Lu et al., 2022, Hou et al., 2019), which is feasible on most scanners used in standard clinical practice. Scans with a very short TR [< 1000 ms (range = 460–––900 ms); as in the studies by Boroda et al., 2021, Cassoudesalle et al., 2020, Rangaprakash et al., 2017, Rangaprakash et al., 2018, Mayer et al., 2019, Meier et al., 2021] can capture more rapid changes in the signal and provide a better sampling of physiological noise (from respiration and cardiac pulsation) that can then be filtered out (Jahanian et al., 2019, Raimondo et al., 2021). These fast rsfMRI scans allow the exploration of dynamic and transient brain states that change over shorter time intervals (see critical reviews by Preti et al., 2017; Zalesky et al., 2014).

The range of spatial resolutions (i.e., voxel size) common across rsfMRI studies of TBI was between 1.2 mm^3^ and 4 mm^3^ (see Supplementary Table). A small isotropic voxel size (e.g., 2.5 mm^3^) is optimal as it provides higher spatial resolution and reduces partial volume effects (Raimando et al., 2021; Van Dijk et al., 2010). Moreover, a large field of view (FOV) (e.g., 250 mm x 250 mm) and many slices (50–70) are ideal, as they reduce the spatial aliasing and increase the SNR, although the additional collection time might be considered for patient tolerance. Several rsfMRI studies in TBI cropped the cerebellum; however, this subcortical region is very important in TBI impairment and recovery, therefore this practice is best avoided (Keleher et al., 2022, Caeyenberghs et al., 2009). It is also recommended to acquire fieldmaps or additional (two or more) reverse phase encoded spin echo images for distortion correction (Raimondo et al., 2021).

As can be seen in the Supplementary Table, there is high variability across rsfMRI studies in terms of acquisition of rsfMRI data with eyes open (e.g., Shumskaya et al., 2017, Konstantinou et al., 2019), eyes fixated on a crosshair (e.g., Bruijel et al., 2022, Grossner et al., 2019), or eyes closed (e.g., Lancaster et al., 2019; Threlkeld et al., 2018). Although these conditions produce comparable FC results, several studies have shown that acquiring rsfMRI data with eyes fixated on a cross may reduce variability (more control of eye movements), show greater reliability of within-network connections (Patriat et al., 2013, Zou et al., 2015), and show more significant correlations with demographic and behavioral variables (Agcaoglu et al., 2019). Recent work (Vanderwal et al., 2015, Gal et al., 2022) has also demonstrated the benefits of collecting rsfMRI data while participants are exposed to naturalistic stimuli, such as watching abstract shapes (e.g., headspacestudios.org/inscapes), instead of fixating on a crosshair. These studies have revealed that naturalistic-stimulus-derived FC predicted individual brain activity and cognitive traits more accurately than rs-derived FC, and improved participant compliance related to motion and wakefulness. These naturalistic stimuli will provide a powerful tool of studying brain organization in TBI patients. Finally, it is also important to consider to monitor physiological parameters (such as respiration and heart rate) and eye tracking of the TBI patients during the rsfMRI acquisition, which can be corrected for during the preprocessing of the rsfMRI data.

### 3.4 Standardize quality assessment procedures of rsfMRI data

Besides optimizing rsfMRI acquisition protocols, it is crucial to ensure the quality of the data to obtain meaningful and reliable results. As seen in the Supplementary Table, several studies (n = 115) did not report performing quality assessment steps on the rsfMRI data. Quality assessment of rsfMRI data will ensure that the acquired fluctuations in the BOLD signal truly reflect biological signals of interest and are not contaminated by noise or artifacts, such as head motion, physiological noise, high intensity wrap-around, scanner drift, susceptibility-related artifacts (Caballero-Gaudes and Reynolds, 2017, Friston et al., 1996, Power et al., 2015). While quality of the raw data is assessed in many laboratories, the details of the assessments are not always reported, possibly because conventions are lacking for reporting types of data quality checks and the measures to be reported. Further, there is a risk of assessment of the quality of preprocessed data being overlooked when using pipelines. We suggest details on all quality assessment measures be included in manuscripts. High-quality rsfMRI data leads to more robust findings and enhances the reproducibility of results of FC patterns in TBI patients, which allows different research groups to compare and validate rsfMRI findings in future *meta*-analyses.

**Fig. 2:**
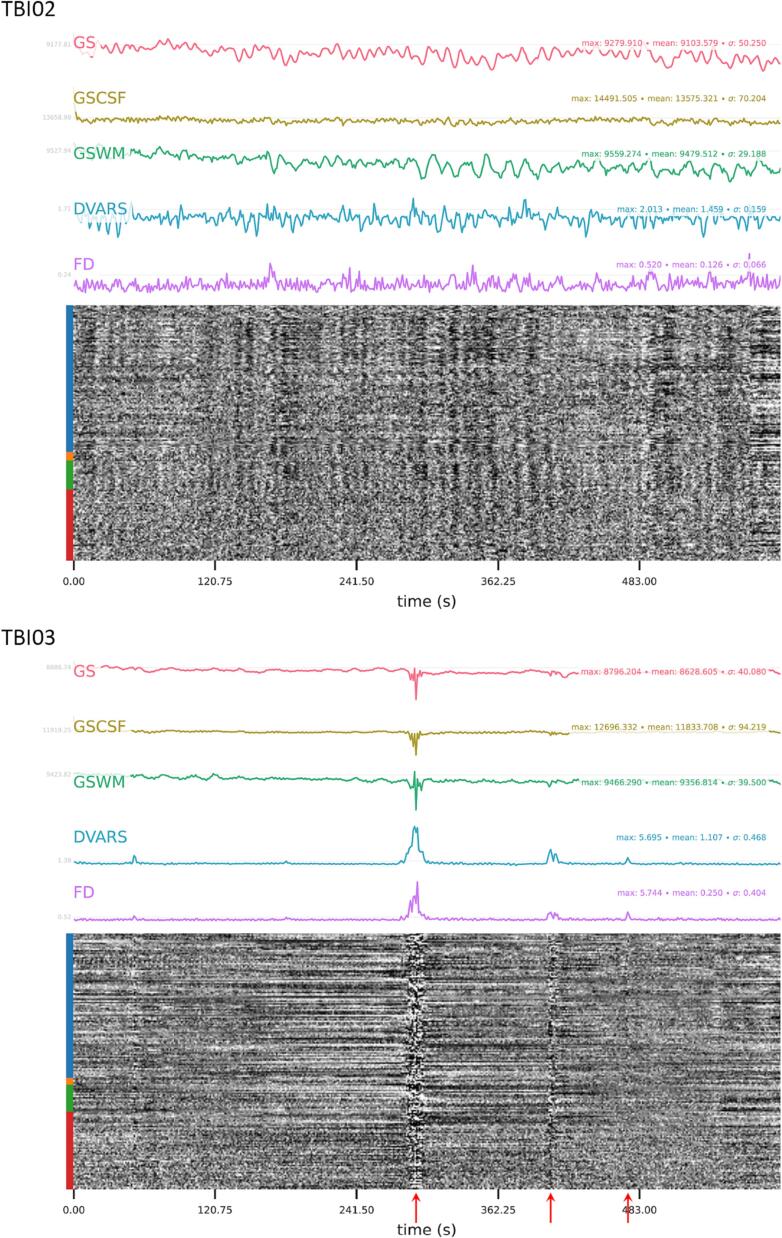
EPI Confounds output from a HALFpipe QC report for two mild TBI patients with good (top) and bad (bottom) data (from an ongoing study led by KC). Displayed are time courses (x-axis) of the magnitude (y-axis) of the global signal (GS), global signal in CSF (GSCSF) and white matter (GSWM), DVARS (D: temporal derivative of time courses; VARS: RMS variance over voxels) and the framewise displacement (FD). The time courses are followed by a carpet plot, a two-dimensional heatmap of the BOLD time series, with time on the x-axis and voxels on the y-axis. Voxels are arranged into cortical (blue) and subcortical (orange) grey matter, cerebellum (green), and white matter and cerebro-spinal fluid (red). QC involves looking for fluctuations in intensity in the carpet plot with reference to motion and signal changes in the time courses. Sudden changes in the carpet plot are likely to be caused by abrupt movement, whereas prolonged signal changes may be indicative of motion or acquisition artifacts. Sustained and substantial changes in the carpet plot of TBI03 (red arrows), particularly around the halfway mark, corresponding to changes in the time courses of all other quality metrics (with a maximum FD > 5 mm), are associated with movement (determined by visual inspection). (For interpretation of the references to colour in this figure legend, the reader is referred to the web version of this article.)

Therefore, it is very important to conduct quality assessment steps on the raw and preprocessed rsfMRI data. Several tools are available that allow users to perform both visual inspection and extraction of quality metrics, such as the configurable pipeline for the analysis of connectome C‐PAC (Craddock et al., 2013), the Conn toolbox for Statistical Parametric Mapping (SPM) (Whitfield-Gabrieli & Nieto-Castanon, 2012), ENIGMA HALFpipe (Waller et al., 2022), the eXtensible Connectivity Pipeline XCP (Ciric et al., 2018), the data processing and analysis of brain images toolbox DPABI (Yan et al., 2016), fMRIPrep (Esteban et al., 2019), Magnetic Resonance Imaging Quality Control tool (MRIQC) (Esteban et al., 2017), FitLins (Markiewicz et al., 2021), Analysis of Functional NeuroImages (AFNI) software suite (Cox, 1996), and SPM (Friston et al., 1995). There are several quality metrics that can be used to distinguish and quantify distinct noise sources in BOLD signal, which can typically be classified into separate domains based on criteria related to their ability to capture spatial versus temporal properties of fMRI time series. In Fig. 2, we present motion quality metrics in two mild TBI patients (one with (bad) and one without (good) the presence of motion artifact) generated by the ENIGMA HALFpipe using the default pre-processing steps and quality assessment criteria (see https://github.com/HALFpipe/HALFpipe#quality-checks for a detailed manual). Of note, these quality metrics are often interrelated, so they potentially represent a common basis set for the TBI field to use in determining image quality moving forward.

In addition to using visual inspection to check for artifacts in raw data, three types of visual inspection of preprocessed data that are commonly used are: (i) Visual inspection of the skull stripping of the anatomical image (to make sure that there are no portions of the brain missing or too much of the skull retained); (ii) Visual inspection of the registration of the anatomical scan to the MNI space (to ensure there is no misalignment between the MNI template and the anatomical scan); and (iii) Visual inspection of the registration of the EPI image to MNI space (to ensure there is no misalignment between the MNI template and the EPI).

Temporal Signal-to-Noise Ratio (tSNR) of the rs-fMRI data is a measure of the ratio of the mean signal to the standard deviation of the signal over time (with higher tSNR indicating better data quality). Global signal (GS) is the average time series of all brain voxels. Drastic fluctuations or outliers in the GS might be indicative of physiological noise or artifacts. DVARS, which is the temporal change in root-mean-square intensity (with D referring to the temporal derivative of the time course and VARS corresponding to the root-mean-square variance over all voxels), indicates the change in BOLD signal across the whole brain at each volume relative to the previous volume (Power et al., 2012). It therefore provides insight into the relationship between the BOLD signal and movement.

Excessive head motion is a common issue in rsfMRI studies in TBI patients as it can introduce spurious correlations or impair the detection of significant ones. Subject motion can be assessed by examining the motion parameters obtained during the preprocessing step (see also section 3.5). The above-mentioned software packages provide quality metrics related to motion correction, which can be used to identify and exclude volumes and participants with excessive motion from the analysis. Quality assessment of motion was reported in 167 of the 181 studies. One of the most common and informative metrics includes mean frame-wise displacement (FD), or the average displacement of the subject’s head between two consecutive fMRI volumes in all six directions (3 translations and 3 rotations) (with higher values of FD indicating more substantial head motion). It is important to note that there is no strict threshold for what constitutes acceptable head motion and the threshold is often driven by the TR of the rsfMRI acquisition (see section 3.3). However, most tools consider FD values below 0.5 mm as relatively low motion, while FD values greater than 3 mm are considered problematic.

While Independent Components Analysis (ICA) is itself an analysis method for rsfMRI data, the assessment of the signal components obtained from ICA decomposition can be used as a quality metric at the level of the individual. The temporal characteristics of each independent component can be inspected for irregularity. Signal components usually have low frequency fluctuations (0.01 – 0.1 Hz) and are fairly regular, while noisy components can be high in frequency with sudden spikes or differing patterns (Griffanti et al., 2017). In studies with healthy subjects, the spatial maps of independent components can be examined to determine if well-defined functional networks (e.g., the default mode network, salience network, visual network, etc) are represented. If the data from the healthy subjects looks correct, the patient data may be examined for maps that are accurate or partially match the standard networks.

### 3.5 Employ consistent approaches for head motion correction procedures and nuisance signal regression

The effects of head motion on data fidelity are now well recognized in rsfMRI data acquisitions (Power et al., 2012, Satterthwaite et al., 2012, Van Dijk et al., 2012). Rigid-body spatial realignment of each acquired volume, spatial and temporal smoothing, and regression of motion parameters (Friston et al., 1996; Birn et al., 2013), are known to be insufficient to mitigate such effects. Since then, a growing literature has aimed at finding the best procedures for data pre-processing to effectively remove BOLD oscillations due to motion while preserving oscillations related to neural activity. This issue is all the more critical in the context of TBI, particularly in acute and moderate and severe cases in both pediatric and adult subjects, since they are well known to have increased incidence of in-scanner motion (Monti et al., 2015; Hannawi et al., 2016).

**Fig. 3:**
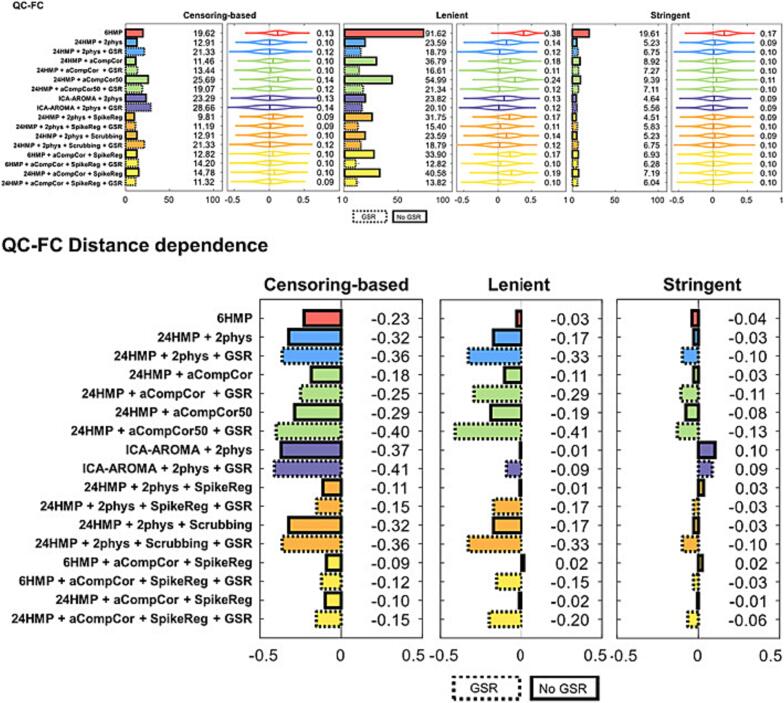
Ability of different preprocessing pipelines (rows) to mitigate spurious head-motion related noise, under different participant exclusion regimens (i.e., censoring-based, lenient exclusion threshold, stringent exclusion threshold), as captured by two quality control metrics (top: the correlation between head motion parameters and functional connectivity, QC-FC; bottom: the degree to which the QC-FC correlation depends on how distant two ROIs are, QC-FC Distance dependence). As discussed in the text, stringent rejection of datasets (i.e., exclude if: <4 min of the data; mFD > 0.25 mm; FD > 0.2 mm in more than 20% of volumes; or any volume has FD > 5 mm; cf., Satterthwaite et al., 2013) minimizes the spurious effects of motion and makes the choice of pipeline secondary, albeit at the cost of potentially large data loss. (Figure adapted from Weiler et al., 2023, OHBM).

It is crucial for studies including TBI populations to use exacting procedures to reduce motion during acquisition and correct the effects of motion during pre-processing. For example, in a recent survey of 88 acute moderate-to-severe TBI patients, application of three different approaches to determine when to reject BOLD data due to excessive motion resulted in the loss of 9% to 32% of datasets (Weiler et al., 2022). When compared across a range of group-level data quality measures (Parkes et al., 2018), the approach resulting in the greatest data loss minimized the spurious effects of motion on brain correlations. Furthermore, under this most stringent data exclusion regime, group-level quality was uniformly high compared to the other approaches, across a range of pre-processing pipelines (cf., Weiler et al., 2022, Fig. 2, Fig. 3). This is very important as it shows that appropriate rejection of datasets corrupted by excessive motion is a primary factor in determining quality of group-level data and allows researchers to then adopt the pre-processing strategy that best conforms to their research question.

Based on a recent comparison of different pre-processing strategies applied to moderate-to-severe TBI patients (Weiler et al., 2022), one of the most crucial aspects of denoising is the appropriate selection of which data to retain for analysis (Satterthwaite et al., 2012, Satterthwaite et al., 2013, Van Dijk et al., 2012). While costly in terms of degrees of freedom for inference, strict rejection of low-quality data (i.e., exclude if: < 4 min of data; mFD > 0.25 mm; FD > 0.2 mm in more than 20% of volumes; or any volume has FD > 5 mm; cf., Satterthwaite et al., 2013) offers several advantages. First, and foremost, it has been shown to be the best strategy for minimizing spurious motion-related associations (Power et al., 2012, Satterthwaite et al., 2012, Van Dijk et al., 2012). Second, when data are of high quality, selection of de-noising in the pipeline becomes secondary and can thus be guided by the nature of the datum (e.g., degree of pathology present) and the analytical approach that best matches the researcher’s aims (Weiler et al., 2022). In the presence of extensive pathology, for example, methods that implement automated tissue segmentation (e.g., AROMA) might be less desirable than methods where segmentations can be performed with customized pipelines (e.g., aCompCor) or methods not requiring any segmentation (e.g., censoring). Similarly, choice of pre-processing strategy can constrain the analytical approaches that can then be applied. Techniques leveraging the frequency of BOLD fluctuations, for example, require the temporal structure of the data not to be altered by pre-processing; thus, pipelines that remove individual timepoints cannot be used (e.g., censoring). Retaining the freedom afforded by high-quality data to select the most appropriate pre-processing pipeline is thus an important aspect of being able to meet one’s intended scientific goals.

### 3.6 Standardize procedures for dealing with lesions in moderate-to-severe TBI patients

There are longstanding issues for handling MRI segmentation and normalization in the context of subcortical and cortical structural abnormalities, which are common in moderate to severe TBI. There have been no gold-standard solutions to date for handling brain lesions in the context of rsfMRI, often resulting in investigators removing any study participant with large identifiable changes due to neurotrauma, which is not ideal and fundamentally changes the scope of our work. There have been both manual (with some of the earliest over two decades ago, see Brett et al., 2001), and automated attempts to handle TBI brain lesions (see Hillary and Biswal, 2009, Diamond et al., 2020), including voxelwise examination of the constituents of the BOLD signal in *peri*-lesional space (e.g., oxygen extraction, cerebral blood flow) (see Hillary and Biswal, 2007). In what follows, we provide simple guidelines and recommendations to alleviate the challenges of automated rsfMRI analysis for TBI patients.

An important difference in the rsfMRI signal between lesioned and non-lesioned tissue is edema and increased water resulting in a distinct signal detectable in T2* data acquisition (Chan et al., 1984, Tang et al., 2020). We recommend that researchers acquire a high-resolution T2*-weighted scan as part of their resting-state study. Doing so is useful to 1) localize edematous lesions and 2) to guide co-registration of T2* scans with T1 volumes. First, brain cells in regions affected by edema often exhibit more oxidative stress and higher rates of catabolic turnover and abnormal reactivity to physiological changes than cells affected by other lesion types, and these changes ordinarily affect the BOLD signal (Toklu and Tumer, 2015). These processes typically affect T2* weighting more so than T1; the higher likelihood of BOLD signal hyperintensities due to these phenomena thus motivates the inclusion of T2* scans with rsfMRI protocols to render TBI-related fMRI signals less challenging to analyze. Second, to prevent co-registration failure in the presence of lesions, a first registration should be used to align rsfMRI scans to T2* weighted scans. In the second step, the T2* scans should be registered to T1 scans. Finally, the two registrations in steps 1 and 2 should be combined to generate a registration to align the fMRI scan to the T1 scan.

**Fig. 4:**
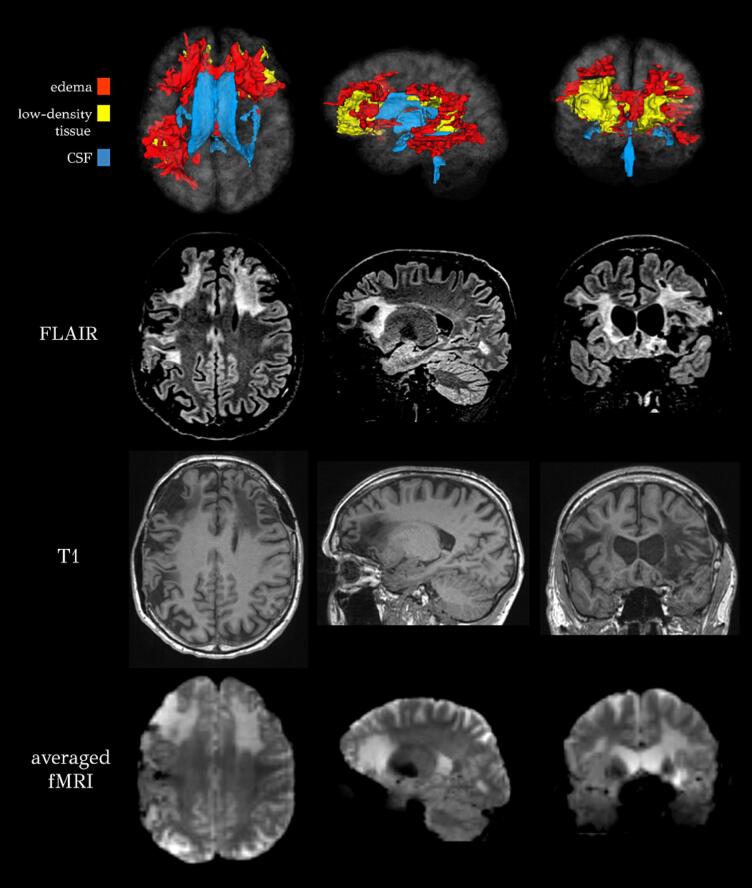
Models of edema (red), low-density tissue (yellow), and cerebral spinal fluid (blue) in a male subject with moderate-severe TBI (49 years old at time of scan, 15 years since injury). Lesion tracing indicates large areas of edema involving anterior and inferior frontal lobes, right lateral temporal and parietotemporal regions extending to the posterior frontal lobe. Low tissue density is observed in the anteromedial aspect of the right thalamus, and the anterior body and genu of the corpus callosum. MRI volumes (FLAIR, *T*_1_-weighted, and averaged rsfMRIs) are provided below the model. Canonical volumetric views (axial, sagittal, and coronal) are displayed in radiological convention for all images. Five trained lesion raters traced edema on FLAIR volumes, and low-density tissue was traced on *T*_1_-weighted MRI. (For interpretation of the references to colour in this figure legend, the reader is referred to the web version of this article.)

Importantly, to identify the spatial profiles of regions with rsfMRI hyperintensities, it is helpful to investigate time-averaged rsfMRI maps. In the presence of posttraumatic edema, the sole inspection of single-time frame functional images may not be helpful because frame-to-frame oscillations in the fMRI signals of edematous areas are relatively less predictable, such that their visibility is poorer than on time-averaged fMRI maps. Such maps can be cross-referenced against fluid attenuated inversion recovery (FLAIR) volumes, where edematous regions are more readily apparent in the presence of cerebrospinal fluid infiltration into the parenchyma (Fig. 4). In the absence of FLAIR scans, one can inspect T1-weighted scans for areas of low phospholipid density (Fig. 4). Such areas may appear hypointense if lipid density is sufficiently low relative to the healthy-appearing background; however, this is likelier for regions within gray matter rather than white matter. We recommend the inclusion of a susceptibility weighted imaging (SWI) scan as part of fMRI acquisition protocols, because T2*-weighted signal drop-out is produced by ferritin and/or hemosiderin accumulation during acute hemorrhage and/or chronic bleeds should be considered as additional factors that can alter the BOLD signal. Lesion boundaries can be difficult to delineate on typical T1- or T2*-weighted scans, but the synergy of SWI as part of a standard fMRI acquisition protocol for TBI patients with rsfMRI protocols can be helpful for lesion delineation (Robles et al., 2022). Because the intensity profile of SWIs is very different from that of fMRI scans, these should not be coregistered directly. Rather, the SWI scan should first be registered to either the T1 or T2* scan, to which the fMRI was already registered as described above.

### 3.7 Use standardized data processing workflows for pre-and post- processing analyses

Adoption of common data pre-processing and analysis procedures will maximize resources and opportunities for between-site data sharing and comparisons between studies. Of course, offering universal guidelines is challenging given the scientific demands of individual labs and investigators. To accommodate the tremendous diversity of pre- and post-processing analytical options (Esteban et al., 2019, Dafflon et al., 2022, Poldrack et al., 2017), there is a critical need for methodological universals that permit direct comparison to the literature. With respect to rsfMRI network analysis, there are (at least) two parts to this problem that require addressing. Both pertain to investigator degrees of freedom or “forking paths” (Gelman & Loken, 2013): the first path is related to data pre-processing and the second to the creation and analysis of functional neural networks.

Adhering to established data processing workflows that are supported by empirical comparisons of pre-processing options is key to addressing issues pertaining to the first “forking path”. fMRIPrep (Esteban et al., 2019) is now a widely used software tool that performs a series of standard pre-processing steps (including data organization, anatomical data pre-processing, fMRI pre-processing, and several quality control metrics) to ensure the reliability of the rsfMRI data. More recently, the ENIGMA consortium has developed HALFpipe (Waller et al., 2022), which is an open‐source tool that facilitates reproducible analysis of rsfMRI data through uniform application of pre-processing, quality assessment, single‐subject feature extraction, and group‐level statistics. This framework was developed specifically for neurological disorders with attention to the consequences of brain injury for structural and functional data (Waller et al., 2022).

It is recognized that while we promote standardization in this critique, the goal is not to stifle creativity or scientific innovation. For investigators who cannot (for whatever reason) employ standard pipelines it is incumbent upon the scientific teams to demonstrate the reliability of the workflow including the effect of idiosyncratic deviations from the standard. This may include testing how each decision point influences data analysis and outcome – an approach now being formalized as multiverse analysis (see Dafflon et al., 2022). Multiverse analysis provides the opportunity to directly examine the most consistent results across a range of data processing decisions. Such analyses have been applied to understand the effect of distinct covariates on analytical outcome (see Demidenko et al., 2022) and may serve as a powerful tool to help in determining the consequences of investigator decisions on analytical outcomes and ensuring that the most robust findings emerge, independent of workflow decisions. These approaches may also aid in developing standards where the gold standard remains uncertain (e.g., some types of motion correction, segmentation with large brain lesions).

Recent critiques have edged the clinical neurosciences toward standardization in network creation and analysis. This second “forking path” allows for fewer investigator decisions than those of pre-processing (Botvinik-Nezer et al., 2020, Esteban et al., 2019), that nonetheless are critical to data interpretation (see Van Dijk et al., 2010, Hallquist and Hillary, 2018). We summarized in Table 1 several key elements that all analyses should include to offer comparable network indices across papers.

**Table 1:** Specific considerations for functional connectomic studies in TBI (Table adapted from Hallquist & Hillary, 2018).

**Issue**	**Recommendation**
*Pre-processing*	Use a standard approach for data pre-processing (e.g., fMRIprep). For decisions that are investigator/lab specific, directly examine the effects of those decision(s) on the outcome (e.g., multiverse analysis).
*Brain parcellation and network size*	A functional atlas should ideally be used for parcellation. Parcellation should include at least 200 nodes, cortical and subcortical structures, and cerebellum. It is best practice to compare distinct parcellation schemes (Craddock, 2012).
*Scope of graph*	Conceptualize and report graph analyses in terms of telescoping levels of analysis, from global (e.g., path length) to regional (nodal strength).
*Edge definition for networks*	The reliability of functional connectomes based on conditional association (e.g., partial correlation) diminishes as the number of nodes increases or the number of measurements decreases (Cassidy et al., 2018).
*Graph metrics*	To promote formal comparisons across studies, report a standard set of graph metrics, even if these are in the form of descriptive tables or supplementary material. As a minimal set, we propose: 1) degree distribution, 2) global clustering coefficient, 3) average path length, 4) modularity, 5) network degree, and 6) summary statistics of edge strength.
*Tests of Reproducibility*	Whenever possible, include analyses of within-subject changes, such as test–retest reliability of effects (e.g., longitudinal data; repeat measurement within session).

## 4 Part 3: Future directions

The goal of our seven recommendations for data acquisition, processing, and analysis of rsfMRI data in patients with TBI is to maximize study reliability and between-site comparability, pushing the field towards the future rsfMRI research in TBI patients. In the following section, we outline new directions for future rsfMRI studies, such as multimodal MRI studies for examining the relationships between structural connectivity and functional connectivity (SC-FC), lesion mapping strategies and single-subject profiling of functional connectivity for making inferences at the single subject level in patients with TBI.

### 4.1 Multimodal MRI studies

Combining complementary information from different imaging modalities may be more fruitful than reporting rsFC metrics in isolation (for a review, see Damoiseaux and Greicius, 2009; Straathof et al., 2019; Suárez et al., 2020). This is especially important for the study of brain-injured patients, as TBI has a cascade of complex effects on the brain that are better captured by multiple modalities. For example, T1-weighted imaging is necessary to ascertain direct and secondary tissue loss and atrophy (Cole et al., 2018) and diffusion weighted imaging provides information about white matter (WM) damage (axonal loss and demyelination (Liang et al., 2021, Poudel et al., 2020)). A recent review has also identified multimodal neuroimaging biomarkers as one of the most promising areas of development, especially given the complexity of multiple secondary injury mechanisms in TBI, stating it may be “helpful to test multiple sources to identify a specific injury profile and tailor treatment more effectively” (Wilde et al., 2022).

As can be seen in the Supplementary Table (indicated by ◊), 53 studies analyzed other modalities in addition to rsfMRI. For example, Diez and colleagues (2017) examined TBI-induced alterations in both functional and structural networks, which showed overlapping results. Specifically, TBI patients demonstrated increased prefrontal connectivity in SC-FC networks, relative to controls. In a study by Raizman and colleagues (2023), the discriminatory power of their logistic regression models yielded higher accuracy if FA (fractional anisotropy) of the corpus callosum was modeled alongside functional connectivity values. Despite these promising multimodal findings, these studies often derived the structural connectivity measures in the form of a single score (e.g., FA value of a white matter tract). However, it is important and informative to reconstruct equidimensional SC-FC indices with respect to the same set of atlas templates (Caeyenberghs et al., 2013). Recent toolboxes, such as Micapipe (Cruces et al., 2022) and Connectivity Analysis Toolbox (CAT) (de Lange et al., 2023) allow researchers to reconstruct SC-FC for the same pair of regions of interest based on (sub)cortical atlases providing aligned connectivity matrices for integrative multimodal MRI analyses. Moreover, in order to extract relevant information from the brain’s structural–functional relationship, it is necessary to validate them against different parameters of another framework, such as graph theory.

Recent developments in network neuroscience facilitate investigating direct structure–function relationships. Kuceyeski and colleagues (2016) published one of the first studies using an advanced integrative network analysis to facilitate the integration of multiple sources of information. Specifically, they used a network diffusion model, which relates an individual's structural and functional connectomes by assuming that functional activation diffuses along structural pathways, to capture functional rerouting. Their findings revealed strong significant correlations between level of consciousness and network diffusion model propagation time (i.e., the time functional activation spends traversing the structural network) in severe TBI patients. In a recent study by Parsons et al (2023), the authors applied another novel mathematical framework, known as a multiplex network analysis (De Domenico et al., 2016), to quantify the proportion of direct and indirect SC-FC connections across the brain. They extracted the degree of structural connectivity between the functional synchronous nodes across the brain network, i.e., SC-FC Multiplex Bandwidth. Their multiplex analyses revealed that direct and indirect SC-FC Bandwidth predicted processing speed in mild TBI patients. Moreover, a subnetwork of interhemispheric edges with increased SC-FC Bandwidth was identified at the chronic, relative to the acute mild TBI post-injury interval. The increased interhemispheric SC-FC Bandwidth of this network corresponded with improved processing speed at the chronic post-injury interval. These integrative network analyses are novel and make a nice contribution to the rsfMRI literature in TBI patients, as they shed light on the relationship between connectomics and impairment/recovery after TBI. Future work should use more advanced models (including multilayer network analysis, neural mass models, etc) to study relationships between relevant variables across several brain metrics that may underpin behavioral outcomes in TBI patients.

### 4.2 Lesion mapping strategies

As raised in recommendation 3.6, there are no gold-standard solutions to date for handling brain lesions in the context of rsfMRI analyses. However, it is common practice in the TBI literature to compare whole-brain FC maps between groups without masking out lesioned areas (see Supplementary Table). The lesion masks can be generated using (semi) automated lesion identification tools (Sanjuán et al., 2013, Seghier et al., 2008), in conjunction with expertise from neurologists and neuroradiologists to enhance the accuracy of the lesion maps and clinical relevance of the rs-fMRI findings. For example, in Rigon and colleaugues (2016) T1-weighted images were inspected for focal lesions and manually traced using FSLVIEW and these resulting lesion masks were used during special normalization with FNIRT to increase the accuracy of the alignment to MNI space.

Another potential strategy is to verify whether FC differences between TBI patients and controls are due to the presence of focal lesions in the TBI sample. For example, Rigon and colleagues (2016) conducted a separate analysis confirming that differences in interhemispheric FC remained evident even when patients with focal lesions were excluded. Similarly, in work by van der Horn and colleagues (2020), their subgroup analyses were conducted to assess the influence of CT-lesions by comparing network measures between patients with (14 patients) and without lesions (54 patients) on CT scans.

Researchers can also consider adjusting for lesion characteristics (such as number of lesions, lesion volume) in the statistical analyses. This was done in a functional connectome study by Roy and colleagues (2018), whereby total lesion volume (calculated a percentage of lesion volume to total gray matter volume) was used as a predictor of total network strength at 3, 6, and 12 months following moderate and severe TBI. The results demonstrated a near-zero relationship between lesion volume and global network strength. Although white matter volume could also be important to take into account, and the method of lesion segmentation could affect accuracy of volume measures (Guo et al., 2019). Yet, another strategy is to implement ROI analyses focusing on specific brain regions affected by lesions to gain insights into localised effects. It should be emphasized, however, that unlike brain tumor or stroke, the pathophysiology of TBI is not easily isolated to a single location (Aerts et al., 2016). Therefore, convergent findings are often indicators of brain response to insult more generally as opposed to ROI-based injury-brain-behavior response. Thus, there is currently no agreed upon approach for handling conspicuous brain pathology after TBI in FC analyses. While this paper is focused on TBI, future work may be informed by lesion measurement and correction in white matter diseases (Guo et al., 2019). Other recent developments include lesion identification and in-painting of brain lesions, via virtual brain grafting or deep learning approaches (Almansour et al., 2021, Liu et al., 2021, Radwan et al., 2021) that may substantially improve brain parcellation thus reducing the extent to which individual regions or whole TBI patients need to be excluded from analysis due to failure.

### 4.3 Single-subject analyses

The majority of the rsfMRI studies (see Supplementary Table) have focused on *group-wise* comparisons in functional connectivity metrics (i.e., N patients vs M controls). These traditional group analyses cannot adequately reflect what happens in individual TBI patients or handle between-patient heterogeneity (Covington and Duff, 2021). Moreover, clinicians need to perform diagnostic and prognostic inferences at the level of *individual* TBI patients. There are several recent rsfMRI studies that exploit TBI heterogeneity with the aim of individualizing approaches to treatment.

In recent years, rsfMRI has been integrated in the clinical practice, where it is utilized to obtain personalized brain stimulation targets and to understand each patient’s brain-based changes in said targeted networks. For example, a proof-of-concept study combining data from five studies demonstrated that using multimodal neuroimaging (including rsfMRI) helps to customize Transcranial Magnetic Stimulation (TMS) treatment, by targeting specific brain areas and networks in mild and severe TBI patients with co-occurring depression, alcohol use disorder, and cognitive dysfunction (Herrold et al., 2020). Relatedly, analysis of rsFC at the network level has been suggested to be more important in TBI patients where injury may have further increased the inter-individual variability already observed across healthy brains (Gordon et al., 2017). Several studies have shown that focusing on rsfMRI subnetworks associated with complex symptoms reported across TBI patients may be the best way to tackle the challenge of interindividual variability (Herrold et al., 2020, Siddiqi et al., 2020, Siddiqi et al., 2023, Sultana et al., 2023). For example, the cingulo-opercular network and the dorsal attention network can be differentially targeted within the dorsolateral prefrontal cortex to address distinct clusters of cognitive deficits or mood problems. These two networks could be differentiated and identified in a patient with severe TBI and a comorbid disorder of consciousness (Siddiqi et al., 2020). Resting-state fMRI is being increasingly adopted to aid with both diagnosis and prognosis across several neurological disorders, such as schizophrenia, Alzheimer's disease, and Parkinson's disease (e.g., Baker et al., 2014, de Vos et al., 2018, Franzmeier et al., 2020, Wolters et al., 2019, Woodward et al., 2012). Similarly, in the context of severe brain injury, recent translational guidelines recommend including rsfMRI in the diagnostic and prognostic process for patients with disorders of consciousness (Kondziella et al., 2020).

Despite this emerging base of empirical work over recent years, there are only a few available neuroimaging-based clinical tools (Scarpazza et al., 2020) that allow single-subject level inferences to be made. Specifically, there is a need to develop patient-tailored frameworks that produce detailed subject-specific characterization of rsFC, including regional alterations, and changes in subnetworks, and network metrics of entire functional connectomes. The resulting individual profiles can then be evaluated against reference populations, such as a group of orthopedic or healthy controls. This contextual information can enable us to meaningfully, qualitatively, and quantitatively, assess rsFC alterations in single individuals. These individual brain measures can be used by clinicians for integrative neuroscience-guided rehabilitation (Dichter et al., 2012, Stoeckel et al., 2014, Wing et al., 2017), assisting them in designing personalized rehabilitation programs (based on the unique profile of each patient), track their progress and adjust care as necessary. Recent studies already successfully employed this novel single-subject brain profiling in TBI patients, albeit in small TBI samples using *structural* MRI metrics (Imms et al., 2023, Clemente et al., 2023, Attye et al., 2021, Jolly et al., 2020). These single-subject analyses will require the development of large reference cohorts of healthy controls (N > 100) (Scarpazza et al., 2020) that are stratified by age group, gender, and other important demographic variables (such as level of education, see section 3.1) to detect FC abnormalities at the level of the individual patient as statistical deviation from the reference group, as done in the ENIGMA Lifespan Working group using morphological brain metrics (CentileBrain, Ge et al., 2023). Such profiling will enable clinicians to progress from the traditional paradigm of group-based comparisons of TBI patients against controls, to a personalized medicine approach, taking us a step closer to translational integrative research that informs clinical practice.

## 5 Conclusion

There remains tremendous promise in the application of rsfMRI in the study of neurological disorders, and in particular TBI. To date, however, this potential has yet to be realized, slowed by limited sample sizes that fail to address clinical and demographic heterogeneity and the sheer volume of analytical approaches, rendering most findings as isolated observations that are irreproducible. In response to these widely recognized concerns in the fMRI literature, the goal of this position paper was to summarize the current literature and then offer directions for a path forward applying rsfMRI to the study of TBI. First, there is a need for data sharing and larger samples. Second, there is a need for greater consistency of methods and analyses to facilitate contributions to the literature that permit synthesis and more direct interpretation. There is also a need for more transparency with respect to analytical pipelines and making data and code available for confirmation of findings within the broader TBI community. While not all recommendations offered here will be agreed upon by the community, if this paper elicits conversations that achieve consensus on methods and procedures, then the field will benefit greatly.

## CRediT authorship contribution statement

**Karen Caeyenberghs:** Conceptualization, Writing – original draft, Writing – review & editing, Visualization, Investigation, Methodology, Supervision. **Phoebe Imms:** Conceptualization, Writing – original draft, Writing – review & editing. **Andrei Irimia:** Conceptualization, Writing – original draft, Writing – review & editing, Visualization, Supervision. **Martin M. Monti:** Conceptualization, Writing – original draft, Writing – review & editing, Visualization. **Carrie Esopenko:** Conceptualization, Writing – original draft, Writing – review & editing. **Nicola L. de Souza:** Writing – original draft, Writing – review & editing. **Juan F. Dominguez D:** Writing – original draft, Writing – review & editing, Visualization, Formal analysis. **Mary R. Newsome:** Conceptualization, Writing – original draft, Writing – review & editing, Supervision. **Ekaterina Dobryakova:** Writing – review & editing. **Andrew Cwiek:** Writing – original draft, Writing – review & editing. **Hollie A.C. Mullin:** Writing – original draft, Writing – review & editing. **Nicholas J. Kim:** Writing – review & editing. **Andrew R. Mayer:** Writing – original draft, Writing – review & editing. **Maheen M. Adamson:** Writing – original draft, Writing – review & editing. **Kevin Bickart:** Conceptualization, Writing – original draft, Writing – review & editing. **Katherine M. Breedlove:** Writing – review & editing. **Emily L. Dennis:** Writing – original draft. **Seth G. Disner:** Writing – review & editing. **Courtney Haswell:** Writing – review & editing. **Cooper B. Hodges:** Writing – review & editing. **Kristen R. Hoskinson:** Writing – review & editing. **Paula K. Johnson:** Writing – review & editing. **Marsh Königs:** Writing – review & editing. **Lucia M. Li:** Writing – review & editing. **Spencer W. Liebel:** Writing – review & editing. **Abigail Livny:** Writing – review & editing. **Rajendra A. Morey:** Writing – review & editing. **Alexandra M. Muir:** Writing – review & editing. **Alexander Olsen:** Writing – review & editing. **Adeel Razi:** Writing – original draft, Writing – review & editing. **Matthew Su:** Writing – review & editing. **David F. Tate:** Writing – review & editing. **Carmen Velez:** Writing – review & editing. **Elisabeth A. Wilde:** Writing – review & editing. **Brandon A. Zielinski:** Writing – review & editing. **Paul M. Thompson:** Writing – review & editing, Supervision, Resources. **Frank G. Hillary:** Conceptualization, Writing – original draft, Writing – review & editing, Visualization, Investigation, Methodology, Supervision.

## Conflicts of interest

MMA is on the scientific advisory board for Polaris Genomics. AO is a co-founder and owner of Nordic Brain Tech AS.

## Funding

KC acknowledges the Victorian Near-miss Award Pilot, administered by veski for the Victorian Health and Medical Research Workforce Project on behalf of the Victorian Government and the Association of Australian Medical Research Institutes. Funding for the Pilot has been provided by the Victorian Department of Jobs, Precincts and Regions. AI, PI, and NJK acknowledge NIH grants R01 NS 100973, RF1 AG 082201, and R01 AG 079957, DoD contract W81XWH-18-1-0413, an anonymous donor family, the Hanson-Thorell Research Scholarship Fund at the Leonard Davis School of Gerontology, the Undergraduate Research Associates Program (URAP), the Provost’s Undergrad Research Fellowship (PURF), and the Center for Undergraduate Research in Viterbi Engineering (CURVE) at the University of Southern California. MMM acknowledges the Tiny Blue Dot Foundation and NIH grant 5 R01 GM 135420. ED acknowledges R01-NS121107. MMA acknowledges the VA R&D SSPIRE award. ELD acknowledges R61NS120249. SGD acknowledges VA RR&D IK2RX002922-01A1. CBH acknowledges High Impact Doctoral Research Assistantship from Brigham Young University. KRH acknowledges NICHD grant K01HD083459. LML acknowledges DOD USAMRMC (PT13078). AL-E acknowledges Israel Innovation Authority, “Magneton” Grant. RAM acknowledges the VA Mid-Atlantic Mental Illness Research Education and Clinical Center (MIRECC) for Post-Deployment Mental Health. AO acknowledges the Liaison Committee between the Central Norway Regional Health Authority (RHA) and the Norwegian University of Science and Technology (NTNU) (2020/39645). FGH acknowledges PA Health Research Grant SAP #4100077082. Frank Hillary is supported with NIH funding (R33 NS 120249).

## Declaration of competing interest

The authors declare that they have no known competing financial interests or personal relationships that could have appeared to influence the work reported in this paper.

## Data Availability

The data that has been used is confidential.

## Appendix A Supplementary data

Supplementary data 1

Supplemental Table References
