# Supplementary material for: ENIGMA’s simple seven: Recommendations to enhance the reproducibility of resting-state fMRI in traumatic brain injury
Source: Neuroimage Clin. 2024 Mar 5;42:103585. doi: 10.1016/j.nicl.2024.103585 (PMC10982609; doi:10.1016/j.nicl.2024.103585)
Supplement: Supplementary data 1 [file mmc1.docx]

### **Supplementary Table. Summary of the rsfMRI studies in TBI patients**

**List of abbreviations:** mTBI= moderate TBI patients; msTBI= study includes both moderate to severe TBI patients**;** sTBI= study includes only severe TBI patients**;** CSF= cerebrospinal fluid; WM= white matter**;** TIV= total intracranial volume; M= male**;** F= female**;** T= Tesla; ICA= independent component analysis**;** TR= repetition time; DOC= disorder of consciousness; N/A= not available**;** QA= quality assurance; rsfMRI= resting state functional magnetic resonance imaging; RTP= return to play**;** ACU= acute**;** SUB= subacute; hr= hours; d= days**;** w= weeks**;** mo= months**;** y= year; ◊=multimodal MRI study

| **Study name** | **Sample** | **rsfMRI acquisition** | **QA rsfMRI data** | **Dealing with lesions** | **Dealing with motion** | **Covariates** | **Post-processing analyses** |
| --- | --- | --- | --- | --- | --- | --- | --- |
| ***Moderate to severe TBI*** | | | | | | | |
| Nakamura et al., 2009 | **8 sTBI**  5M/3F  Age range = 19-51y  Time since injury = 3 – 6mo | - 3T - TR = 2000ms - 166 volumes/run - 6min 32s - Resting state data extracted from task-related fMRI - Eyes open/closed not reported | N/A | N/A | 1 subject excluded due to significant motion. | N/A | Graph theory |
| Zhang et al., 2022 | **30 sTBI suffering from DOC**  19M/11F  Age = 58±15.39y  Time since injury = 1 – 4w | - 3T - TR = 2000ms - 8min - Eyes open/closed not reported | N/A | N/A | > 2mm translation and > 2degrees rotation. 2 patients removed due to significant motion. | CSF, WM, head motion coefficient, age, gender. | Seed (DMN) to whole brain, machine learning. |
| Zhang et al., 2022 | **28 sTBI**  19M/9F  Age = 58±15.39y  Time since injury = 2w | - 3T - TR = 2000ms - 8min - Eyes open/closed not reported | MRI-cro software. | N/A | > 2mm translation and > 2degrees rotation. | CSF, WM, head motion coefficient, age, gender. | Graph theory |
| Shumskaya et al., 2017 | **47 msTBI**  25M/22F  Age range = 18 – 65y  Time since injury = chronic | - 3T - TR = 2000ms - 800 volumes - 3.5x3.5x3mm^3^ - Eyes open (in darkness) | N/A | N/A | 3 patients removed due to excessive movement (>3mm). 6 motion parameters and 6 frame-to-frame parameters. | CSF, WM, 24 motion parameters, root mean square of relative motion. | Group ICA |
| Konstantinou et al., 2019 | **11 msTBI**  11M  Age = 34.11±11.92y  Age range = 24 – 60y  Time since injury = 127.33±83.68mo, > 24mo | - 3T - TR = 3000ms - 160 volumes - 2.4 x 2.4 x 4mm^3^ - Eyes open | Visual inspection. | N/A | 1 patient removed due to signification motion artefacts. | Age, years of education, time since injury, motion parameters. | Global FC (intrinsic connectivity contrast), seed-based analyses. |
| Hillary et al., 2011 | **10 msTBI**  7M/3F  Age = 29.4±11y  Time since injury = 3mo, 6mo | - 3T - TR = 2000ms - Resting state data extracted from task-related fMRI - 165 volumes/run - Eyes open/closed not reported | Trials with poor SNR were eliminated. | N/A | Trials with motion were eliminated. | CSF, WM, mean BOLD, motion parameters. | ROIS |
| ◊ Kazazian et al., 2020 | **1 sTBI**  1M  Case study  Age = 34y  Time since injury = 26d | - 1.5T - TR = 2500ms - 3.75 x 3.75 x 5mm^3^ - 132 volumes - 5min 30s - Eyes open/closed not reported   2 scanning timepoints = 26d + 9mo | N/A | N/A | N/A | N/A | ICA |
| ◊ Raizman et al., 2022 | **21 msTBI**  17M/4F  Age = 32.9±9.22y  Time since injury = 3 – 4mo | - 3T - TR = 2000ms - 300 volumes - 3mm^3^ - Eyes open/closed not reported | N/A | 5 patients with large lesions (seen through MRI) were excluded. | Correction for head motion, unwarping the images. | CompCor method, WM, CSF, noise from other sources. | Homotopic FC, seed-based analysis. |
| Chou et al., 2022 | **93 mildTBI**  **45 severeTBI** | 2 datasets:   - 3T - TR = 2000ms - 3.43 x 3.43 x 3mm - 206 measurements - 3T - TR = 2000ms - 3mm x 3mm x 3.97mm - 210 measurements - Eyes open/closed not reported | N/A | N/A | N/A | N/A | - ICA - Siamese neural networks |
| Venkatesan 2015 | **22 msTBI**  14M/8F  Age = 34.0±15.08y  Range time since injury = 0.5 – 5.67y  Median time since injury = 2y | - 3T - TR = 2000ms - 150-180 volumes - Eyes open/closed not reported | N/A | N/A | N/A | WM, GM, CSF, six motion regressors. | Seed to voxel analysis. |
| ◊Martina-Molinez | **40 msTBI**  24M/16F  Age = 41.3y  Time since injury = 8.9mo | - 3T - TR = 2000ms - 145 volumes - Eyes open fixation cross   3 scanning timepoints, baseline, 3mo, 6mo | N/A | Cost function masking (for subgroup of patients with focal lesions), lesion masks. | Artifact Repair Toolbox, >3SD z-scores global BOLD signal, >2mm linear motion. | Number of outliers. | FC of subnetworks - seed-based connectivity analyses. |
| Bernier et al., 2017 | **19 msTBI**  11M/8F  Age = 29.5±13.3y  Time since injury = 1y | - 3T - TR = 2000ms - 3mm x 3mm x 4mm - 145 measurements - Eyes open | N/A | N/A | Artifact Repair Toolbox (SPM8). Subjects with >25% volumes requiring interpolation due to motion were removed. | N/A | Graph theory (strength and degree). |
| Lancaster et al., 2019 | **21 msTBI**  18M/3F  Age =  Time since injury = 112.47±97.95mo | - 3T - TR = 2000ms - 6min - 2.3 x 2.3 x 3mm - 180 volumes - Eyes closed | N/A | N/A | ArtRepair toolbox. Subjects with greater than 20% removed (4 participants). | N/A | Functional connectivity analyses, DMN ROIs. |
| Gilbert et al., 2017 | **23 msTBI**  11M/12F  Age = 31.74±12.90y  Time since injury = 4.2±5.8y | - EPI - TR = 2000ms - 230 x 230mm^3^ - 480 volumes - Eyes open/closed not reported | N/A | N/A | ArtRepair Toolbox. Subjects with motion >20% volumes removed. | N/A | ICA, graph theory (static/dynamic FC). |
| ◊Vaughn et al., 2021 | **50 mild TBI**  33M/17F  Age = 12.33±2.17y  20 msTBI  10M/10F  Age = 12.50±2.5y | - 3T - Gradient echo EPI - TR = 2000ms - 1.25 x 1.25 x 3.50mm^3^ - 6min 6sec - Eyes open/closed not reported | N/A | N/A | Motion correction through realignment, Artirfact Detection Tools (ART) toolbox. Subjects with motion >15% volumes removed. | N/A | Bayesian multi subject vector autoregressive modelling (BVAR-connect). DMN effective connectivity. |
| ◊Grossner et al., 2019 | **21 msTBI**  12M/9F  Age = 32.9±14.0y | - 3T - TR = 2000ms - 10min - 3 x 3 x 4 mm - 145 volumes - Eyes open fixated on cross | Co-registration and 6mm isotropic smoothing in SPM8 to improve SNR. | N/A | ArtRepair. Subjects with movement in >20% of volumes were removed. | N/A | Graph Theory, network strength for 6 subsystem networks. |
| ◊Diez et al., 2017 | **14 msTBI**  6M/8F  Age = 13.14±3.25y | - 3T - TR = 3000ms - 10min - Gradient echo EPI - 2.5 x 2.5 x 3.1 mm - Eyes open/closed not reported | N/A | Stated that ‘all 14 had DAI and no severe focal lesions “independent of lesions observed during the acute scan’, where they did have focal lesions. | Head movement artifacts corrected by aligning each volume to the middle volume. Regressed out movement in time-courses. To remove effect of head movement in group comparison, global frame displacement was used as a covariate of non-interest. | Postural control, variance/kurtosis/skewness/PPA. Age and average frame displacement were covariates of non-interest. | PCA, ICA. |
| Guo et al., 2018 | **21 sTBI coma**  18M/3F  Age = 38.0±10.66y | - 3T - EPI - TR = 2000ms - 180 volumes - 6min - Eyes open/closed not reported | DPARSFA 2.3 toolbox (pre-processing in SPM8). | N/A | N/A | Head movement, white matter signal, CSF signal were removed prior to FC analysis. | Posterior cingulate cortex ROI FC analysis. |
| Threlkeld et al., 2019 | **17 sTBI coma**  15M/2F  Age = 29±9y | - 3T - TR = 2400ms - 3.4 x 3.4 x 3.5 mm - Eyes closed - 7min 12sec | CONN toolbox. | “No lesion extraction or masking was performed”. | Artifact rejection tool (ART) toolbox. Subjects with absolute motion >(or=to)0.5mm. | Motion parameters and first temporal derivatives included as first-level covariates. | Seed based DMN connectivity. |
| Thompson et al., 2016 | **24 msTBI**  24M  Age = 36.7  Age range = 16 – 67y  Time since injury =  8d (range = 1 – 22d) | - 1.5T - TR = 2500ms - 2 x 2 x 2 mm - 200 volumes - 8min 30sec - Eyes open/closed not reported | Data from one patient was excluded as it could not be pre-processed accurately. CONN toolbox v 14.k. | Data from one patient was excluded due to “difficulties with co-registration and normalization of the MRI imaging data due to extensive anatomical pathology”. | N/A | Combination of drugs administered (reduced by correspondence analysis) and age were used as nuisance regressors. | Multi-variate pattern analysis (MVPA)-informed seed-based correlation analysis, “connectome-MVPA” CONN toolbox. |
| Newsome et al., 2013 | **9 msTBI**  5M/4F  Age = 17.67±1.75y | - 3T - “BOLD T2* single shot gradient EPI” - TR = 1700ms - Eyes open/closed not reported | N/A | All TBI patients had focal frontal lobe lesions on structural MRI. | Excessive motion was observed in 2 patients, these were excluded. Data were spatially registered in 2D and 3D to minimize effects of head motions. | All six movement parameters. ROID based time series for CSF and WM. A constant term and a linear term were entered into a linear regression. | Functional connectivity of rACC and right amygdala seeds. |
| Rigon et al., 2016 | **21 msTBI**  11M/10F  Age = 51.76±4.7y  Time since injury = > 6mo | - 3T - T2*w rsfMRI – EPI - TR = 2000ms - 3.4 x 3.4 x 3.5mm - 6min - Eyes closed | N/A | T1 images were examined for lesions and hand traced using FSLVIEW. Masks were used during spatial normalisation to increase the quality of the fit to the MNI brain. | Motion correction using MCFLIRT. | N/A | ICA & Seed voxel correlation analysis. |
| Rigon et al., 2017 | **26 msTBI**  16M/10F  Age = 50.92±15.09y | - 3T - T2*w rsfMRI – EPI - TR = 2000ms - 3.4 x 3.4 x 3.5mm - 6min - Eyes closed | Two participants were excluded because of T1 or EPI artifacts. | N/A | Motion dealt with using nuisance regression and motion scrubbing. | Psychiatric diagnosis, sex, age. | Network-based statistics. |
| ◊Sours et al., 2017 | **27 sTBI**  16M/11F  Age = 29.3±8.9y | - 3T - EPI - TR = 2000ms - 171 volumes - 5min 42sec - Eyes closed | ConnToolbox. | N/A | Motion was dealt with using nuisance regression. | Age. | Seed-based FC analysis. |
| Bruijel et al., 2022 | **16 msTBI**  12M/4F  Age = 37.1±13.6y  Time since injury = 25±2.2mo | - 3T - EPI - TR = 2000ms - 2mm^3^ - 350 volumes - Eyes open fixation cross - Two EPI scans with reverse phase encoding were acquired to create field maps for susceptibility distortion | ConnToolbox. | N/A | Identified scans with excessive motion for scrubbing volumes (identified using 95^th^ percentile, global signal z-value threshold of 3, and subject motion threshold of 0.5mm.) Conducted analyses of group differences in motion between TBI and HCs. No subjects were removed despite motion. | Sex, age. | Seed-to-voxel analysis. |
| Hillary et al., 2014 | **21 msTBI**  18M/3F  Age = 27.9±9.1y  Time since injury = 113.5±32.3d | - 3T - TR = 2000ms - 135-142 volumes - Eyes open/closed not reported | Trials containing significant motion were discontinued or repeated. | N/A | ArtRepair was used to identify slice and volume movement effects using recommended cut-offs (5% slices and 25% volumes). 1 TBI patient removed for significant framewise motion. | N/A | ICA, Graph Theory. |
| ◊Gębska-Kośla et al., 2020 | **1 sTBI**  1M  Age = 66y  Time since injury = 2y | - 1.5T   Task:   - EPI - TR = 3000ms - 3mm^2^   Rest:   - TR = 2500ms - 3.5mm^2^ - Eyes open/closed not reported | N/A | N/A | N/A | N/A | Voxelwise t-scores. |
| ***mildTBI*** | | | | | | | |
| ◊Zhao et al., 2017 | **16 mTBI** | - 3T - TR = 2000ms - TE = 30ms - 240 volumes - Sequence Time: Not given (calc: 8 min) - 3.125 x 3.125 mm - Eyes closed | Removal of unspecified “problematic” cases. | N/A | FSL FEAT for motion correction. | N/A | 10 resting state network atlases from Smith et al., 2009, + Convolutional Autoencoder derived network atlases (data driven). FC values (identification of abnormal subnetworks for mTBI). |
| Wright et al., 2020 | **20 mTBI + fatigue**  7M/13F  Age = 48.0±9.2y  Time since injury = 21.2±17.9y | - 3T - TR = 3660ms - TE = 39ms - 164 volumes - 10min - 2.6 x 2.6 x 4 mm - Eyes open, cued to avoid thinking of specific thoughts, no mention of fixation point | N/A | 5 patients with large lesions (seen through MRI) were excluded. | CONN functional connectivity toolbox -realignment and unwarping for motion correction. | Change in score for each MOI, total intracranial volume (nuisance covariate). | Intrinsic Connectivity analysis. (Whole brain - Atlas not given. Additional ROI seed-based analysis for 10 regions selected a-priori). |
| Woytowicz et al., 2018 | **7 mTBI mild balance impairments+**  Age = 47.0±14.78y  **7 mTBI mild balance impairments-**  Age = 46.86±15.18y  12M/2F  Age range = 27 – 70y  Time since injury = 2 – 8w | - 3T - TR = 2300ms - TE = 30ms - 2.4 x 2.4 x 4 mm - Eyes open/closed not reported | N/A | Initial exclusionary criteria, otherwise N/A. | Motion correction using MCFLIRT + T test analysis of absolute and relative motion between groups. | Only stated in post-hoc: Individual subject’s L pre-SMC integration values (pre-supplementary motor cortex). | Group ICA for common reference network maps (resulting in seed based of 2 reference components of interest; both within DMN).  Subject-specific spatial maps. |
| Yuan et al., 2017 | **22 mTBI**  12M/10F  Age = 14.45±1.72y  Time since injury = 4 – 16w | - 3T - TR = 6565ms - Eyes open/closed not reported | N/A | N/A | Head motion correction. | N/A | Graph theory, DTI tractography. (Structual connectivity only). |
| Zhang et al., 2012 | **14 sport-related mTBI**  Sex not specified  Age = 20.8±1.5y  Time since injury = 10±2d | - 3T - TR = 2000ms - 3.1x3.1x5 mm - 6min 4sec - Eyes closed | N/A | No lesions found. | Motion parameters: white matter, CSF, noise source reduction, no GSR. | N/A | ROIs seed based. |
| Xu et al.,  2022 | **10 mTBI PTSD+**  **10 mTBI PTSD-**  Time since injury = 2w  Age + sex N/A | - 235 timepoints - Eyes open/closed not reported   2 scanning timepoints (2w + 3mo post injury). | N/A | N/A | N/A | N/A | ReHo (abnormal Regions of interest (ROIs) created based on average ReHo value). |
| Ye et al.,  2019 | **52 mTBI**  27M/25F  Age = 34.48±13.32y  Time since injury = < 1 w  GCS 13 – 15  LOC < 30min  PTA < 24h | - 3T - TR = 2000ms - Eyes closed, avoid thinking about anything | N/A | The presence of focal lesions and cerebral microbleeds was determined by an experienced clinical neuroradiologist. | Regression: head motion, white matter, CSF. | Gender. | ROIs, seed-based,  (defined by peak foci in canonical regions on the activation map). |
| ◊Zhou et al.,  2017 | **24 symptomatic mTBI**  17M/7F  Age = 34.2±11.4y  Age range = 18 – 56y  Time since injury = 22d (3 – 53d) | - 3T - TR = 2000ms - 153 volumes - 5min 6sec - Eyes closed but stay awake | N/A | Reviewed by two radiologists. No lesions found. | No motion artifacts > 2mm. | N/A | Seed based, ROI (seed region selected in whole hypothalamus). |
| ◊Zhou et al.,  2017 | **30 clinically defined mTBI**  24M/6F  Age= 35±13y  Time since injury = 22d (3 – 53d) | - 3T - TR = 2000ms - 153 volumes - 5min 6sec - Eyes closed but stay awake | N/A | Reviewed by two radiologists. No lesions found. | Regression: AFNI motion correction algorithms, 6 motion parameters, global signal, CSF, WM, residual signal at each voxel. | N/A | Seed-based (44 Broadmann areas from MRIco). |
| ◊Zhou et al.,  2014 | **27 mTBI**  21M/6F  Age = 33.4±11.2y  Time since injury = 23d (3 – 58d) | - 3T - TR = 2000ms - 153 volumes - 5min 6sec - Eyes closed but stay awake | N/A | Reviewed by two radiologists. No lesions found. | Regression: 6 motion parameters, global signal, CSF, WM. | N/A | Seed-based, ROI (whole brain). |
| Boroda et al., 2021 | **42 mTBI**  37M/5F  Age = 50.9±12.1y  Time since injury = 20.7±15.5y | - 3T - TR = 800ms - 8,600 volumes - 8min - Eyes open fixated on cross, don’t think about anything | Image quality for rsfMRI checked using the FSL toolbox and custom MATLAB scripts. | N/A | Software based realignment with 6 motion parameters. Motion correction using methods from Power et al. FD >0.5 mm and/or DVARS >8 along with 1 vol before and 2 vols after. Runs with >25% flagged volumes were rejected. 8 mTBI excluded due to motion. | N/A | Graph theory, ROI  (400 ROI Schaefer Atlas and 19 ROI Harvard-Oxford subcortical). |
| ◊Champagne et al.  2020 | **23 mTBI**  9M/14F  Age = 31.0±11.0y  Time since injury = 14.6±14.9mo (1 – 48mo) | - 3T - TR = 2000ms - Eyes open/closed not reported | N/A | N/A | Regression: Global signal, WM, CSF, and 6 motion parameters. Volumes > 0.2mm displacement were censored prior to smoothing. | N/A | Seed-based, ROIs (Yeo et al 2011 parcellation atlas). |
| Chong et al., 2019 | **15 mTBI**  2M/13F  Age = 39.1±10.1y  Time since injury = 28±9.5d | - Eyes closed but remain awake, relax, and clear mind   2 scanning points, 28±9.5d + 5mo (121±13d) | N/A | N/A | Regression: WM, CSF, global signal, motion. Checked motion with DPARSF. Any rotation over 2 degrees in any direction excluded. Bad time points were flagged if FD >0.5, including timepoints before and after. | N/A | ROIs (29 left and right hemisphere ROIs). |
| Churchill et al.,  2018 | **35 mTBI sport concussion**  16M/19F  Age = 20.3±2.2y  Time since injury = 5d (1 – 7d) | - 3T - TR = 2000ms - eyes closed and don’t focus on anything | N/A | N/A | Regression: WM. Rigid body motion correction (AFNI). Removal of outlier volumes using SPIKECOR algorithm. | N/A | ROIs (defined by novel multivariate approach where intra-network connectivity was associated with symptom severity). |
| ◊Churchill et al.,  2020 | **33 mTBI sport concussion**  16M/17F  Age = 20.3±2.0y  Time since injury = 1 – 7d | - 3T - TR = 2000ms - Eyes closed and don’t focus on anything   3 scanning timepoints: 1-7d, 1mo, 1y | N/A | N/A | Regression: Rigid body motion correction (AFNI). Removal of outlier volumes using SPIKECOR algorithm. | N/A | ROIs (AAL atlas). |
| Dona et al., 2017 | **15 mTBI concussion**  Age = 13.4±2.3y  Time since injury = 33.0±43.8d  Sex not disclosed | - 3T - TR = 2000ms - 180 timepoints - 6min 8sec - eyes open | N/A | N/A | Motion correction using 6 point affine transformation with AFNI 3DVolreg. | N/A | Voxel-based, ROI analysis (TT_Daemon atlas). |
| Han et al., 2018 | **56 chronic mTBI**  35M/21F  Age = 40.5±14.0y  Age range = 20 – 65y  Time since injury = 9y | - 3T - TR = 2000ms - 208 timepoints - 6min 56sec - Remain still with eyes closed   3 scanning timepoints: prior to training, after training, and 3mo after training. | N/A | N/A | Regression:  6 parameters for rigid body, motion, signal from lateral ventricles, deep cerebral white matter, temporal derivatives for these parameters, squared motion parameters, and regressors from band-pass filtering.  Motion correction, 6 parameters. | Age, education, estimated IQ, psychiatric (depression, PTSD) symptoms, post-injury time. | Seed-based (Yeo atlas). |
| Monroe et al., 2020 | **13 mTBI athletes (soccer players)**  13M  Age = 20.2±1.5y | - 3T - TR = 2000ms - Eyes open/closed not reported   2 scanning timepoints: before and after season, within 2 weeks of the beginning and end of potential exposure to head injury. | N/A | N/A | N Regression: aCompCor. CSF (12 components), WM (5 components), head motion (5 components). | Concussions sustained during the season, group membership. | ROI analysis. ROIs were selected for their putative core role in central autonomic regulation (Bennaroch, 2014). |
| Sours et al., 2015 | **28 mTBI**  18M/10F  Age = 38.9±15.9y  Time since injury = 11d (6±3d) | - 3T - TR = 2000ms - TE = 30ms - 171 timepoints - 5min 42sec - Rest peacefully eyes closed   3 scanning timepoints: 11d (6±3d), 1mo (36±13), and 198±26 (137-266). | N/A | N/A | Regression:  WM, CSF, 6 motion parameters.  M Motion Correction. | Age. | ROI analysis (Conn toolbox v13.h created average group networks for the DMN and TPN). |
| van der Horn et al.,  2017 | **54 mTBI**  36M/18F  Age = 37±15y  Age range= 18 – 65y  Time since injury = 4w | - 3T - TR = 2000ms - TE = 20ms - 300 timepoints - Eyes closed stay awake | N/A | Detected lesions with T2. | Regression: WM, CSF, motion.  > 0.5 mm fd were interpolated. | N/A | ICA (fMRI Toolbox). |
| ◊Wu et al., 2022 | **20 mTBI concussive head impacts**  Sex not disclosed  Age not disclosed  Time since injury not disclosed | N/A | N/A | Lesions to structural (white matter tracts). | N/A | N/A | ROIs (cortical parcellation atlas by Schaefer). |
| Abbas et al., 2015 | **22 mTBI collision sports**  22M  Age = 16.7y  Age range = 14 – 18y  Time since injury not disclosed | - 3T - TR = 1500ms - 5 min 30sec - 220 volumes - Eyes open/closed not reported   3-6 imaging sessions, preseason (4w interval before practice), in season (no two sessions < 3w apart), and post season (~5mo after season). | N/A | N/A | Regression: ANATICOR, white matter regressors, 6 motion estimates, GS (global signal), WM, LV (large ventricle signal).  > 0.4 mm were censored, despiking. | N/A | N/A |
| Abbas et al., 2015 | **10 mTBI high school athletes**  10M  Age = 17y  Age range = 16 – 18y  Time since injury not disclosed | - 3T - TR = 2000ms - 9min 46sec - 293 volumes - Eyes open/closed not reported   3 scanning timepoints: 1 preseason (before practice), 2 in season (within 48 hours of game or practice), and 6 postseason (6-month windows). | N/A | N/A | Regression: 6 motion estimates, GS (global signal), WM, LV (large ventricle signal). Despiking. | N/A | N/A |
| Borich et al., 2015 | **12 mTBI subacute concussion**  10M/2F  Age = 15.5±1.2y Age range = 14 – 17y  Time since injury = < 2mo | - 3T - TR = 2000ms - 8min 12sec - 246 volumes - Eyes open fixated on cross eyes on target, don’t fall asleep or think of anything in particular | N/A | N/A | Regression: Motion parameters. Motion correction, > 3.0 mm were excluded (1 subject excluded, not accounted for in n = 12). | N/A | N/A |
| Brett et al., 2022 | **100 mTBI history of sport concussion**  69M/31F  Age = 21.4±1.68y Age range = 18 – 26y  Time since injury = < 12mo | - 3T - TR = 1800ms - 10min - 334 volumes - Eyes open fixated on cross during scan | N/A | N/A | Regression: ANTICOR, WM, 6 motion parameters, CSF.  Despiking: participants with >15% volumes removed excluded. OR participants with Euclidian norm > 0.2 were excluded. | Age, sex. | N/A |
| Cassoudesalle et al., 2020 | **10 soccer players with no history of brain injury**  10M  Age range = 18 – 25y | - 3T - TR = 900ms - 1000 volumes - 15min - 2.5mm voxels (one dimension given) - Eyes closed   2 scanning timepoints: 10 – 12mo apart | N/A | Clinical T2 used to identify abnormalities, potential lesions, no further explanation provided. | SPM12 and CONN for regression of CSF, cerebral WM, motion (12 parameters), and outlier scans. | N/A | Seed-based as well as whole-brain voxel-wise, BOLD temporal correlations (within and between groups). Talairach Daemon labels. |
| ◊Champagne et al., 2021 | **15 combat-related mTBI**  15M  Age = 40.0±5.6y  Time since injury not disclosed | - 3T - TR = 2000ms - 180 volumes - 6min - 3.5mm voxels (one dimension given) - eyes open fixated on cross | N/A | N/A | Motion correction via MCFLIRT+FSL (6 rigid-body parameters), WM and CSF signals. | N/A | ICA, FC mapping. ROI (Yeo Parcellation). |
| Churchill et al., 2017 | **21 mTBI sports-related concussion**  10M/11F  Age = 21.0±1.7y  Time since injury = 26mo | - TR = 2000ms - 193 volumes - 6min 43sec - 3.125 × 3.125 × 4.5mm voxels - Eyes closed | N/A | FLAIR and SWI used for screening of abnormalities and lesions. No participants removed. | Motion correction via AFNI+3dTshift, CSF. | N/A | ROI, seed-based (via pcr), and whole-brain FC maps. AAL parcellation. |
| Churchill et al., 2021 | **45 mTBI sports-related**  23M/22F  Age = 20.3±2.0y  Time since injury = 1w | - 3T - TR = 2000ms - 195 volumes - 6min 30sec - 3.125 x 3.125 x 4.5mm voxels - Eyes closed | N/A | Initial inspection by an MRI technologist during the imaging session and later review by a neuroradiologist with clinical reporting.  No abnormalities (white matter hyper-intensities, contusions, micro-hemorrhage, or statistical outliers) were found. | PCA based 6 rigid-body movement parameters, PHYCAA+ for non-neural, WM, and CSF and regression. | N/A | ROI, whole brain connectivity (Pearson). Brainnetome Atlas (BNA) parcellation. |
| ◊Churchill et al., 2021 | **32 mTBI sports-related concussion**  15M/17F  Age = 20±2y  Time since injury = 39mo  (range= 10 – 61mo) | - 3T - TR = 2000ms - 195 volumes - 6min 30sec - 3.25 X 3.25 X 4.5mm voxels - Eyes open/closed not reported | N/A | Reviewed by technologist and neuroradiologist for abnormalities. | N/A | N/A | Whole-brain connectivity, network level analysis  "Shirer and Colleagues" parcellation -14 networks |
| ◊Costanzo et al., 2014 | **11 mTBI combat-related**  8M/3F  Age = 26.16±5.52y  Time since injury not disclosed | - 3T - TR = 2000ms - 206 volumes - 6min 52sec - 3.43 mm × 3.43 mm × 3mm voxels - Eyes open/closed not reported | N/A | N/A | Motion, CSF, and WM. Regressed, not stated to be removed. | N/A | Seed-based (a priori), FC (Pearson). Harvard-Oxford structural atlas parcellation. |
| D'Souza et al., 2020 | **60 mTBI**  34M/26F  Age = 30.4±10.34y  Time since injury = 7d | - 3T - TR = 2000ms - 205 volumes - 410min - 3.75 X 3.75 X 5mm voxels - Eyes closed   2 scanning timepoints: 7d + 6mo post injury | Independent components (ICs) were generated using FSL FEAT, | Examined by an experienced neuroradiologist. All subjects with any abnormality noted on MRI were excluded from the study. | N/A | Age. | GLM (voxel-wise FC), RS networks of interest, (FC correlations via atlas).  Harvard–Oxford cortical and subcortical atlases parcellation. |
| Dailey et al., 2018 | **15 mTBI**  4M/11F  Age = 21.86±2.79y  Time since injury = 6mo | - 3T - TR = 2000ms - 300 volumes - 10min - 2.5 × 2.5 × 2.5mm voxels - Eyes open fixated on cross | N/A | N/A | SPM 12 for motion correction, CONN functional connectivity toolbox, and artifact detection toolbox (threshold = .5mm). 2 participants excluded. | Depression (controlled for in one model, not necessarily regressor of non-interest). | GLM for FC. Shirer et al parcellation (functionally defined ROI's). |
| de Souza et al., 2020 | **39 mTBI**  29M/10F  Age = 42.28±18.65y  Time since injury = 10d | - 3T - TR = 2000ms - 171 volumes - 5min 42sec - 3.59 × 3.59 × 4mm voxels - Eyes closed   2 scanning timepoints: 10d + 18mo post injury | N/A | N/A | CONN and ART toolbox, WM, CSF, 6-motion parameters. Did not state any were removed. | N/A | Whole brain FC correlations, Seed-based FC. Andrews-Hanna and colleagues derived seeds parcellation. |
| ◊Dudley et al., 2020 | **72 sports-related sub-concussive impact**  72F  ‘High school age’  Time since injury N/A | - 3T - TR = 1200ms - 250 volumes - 5min - 3 x 3 x 3mm voxels - Eyes open fixated on cross   2 scanning timepoints: pre- and post-season | N/A | Visual inspection by board certified neuroradiologist for abnormalities. | Motion regressed w/ SPM12, + >0.5mm movement threshold for removal, 3SD BOLD signal change framewise threshold.  CSF and WM. | N/A | Whole-brain functional correlation matrices (Pearson). Graph theoretical for clustering analysis.  AAL (90, Tzourio-Mazoyer et al) parcellation. |
| Dumkrieger et al., 2019 | **44 veterans with PT headache/migraine**  28M/16F  Age = 37.5±10.8y Age range = 18 – 65y  Time since injury not disclosed | - 3T - TR = 2500ms - 240 volumes - 10min - 4 x 4 x 4mm voxels - Eyes closed | N/A | T2 used to rule out 'gross anatomical abnormalities" - did not report removing any participants or specifics. | SPM 8 - motion correction and regression reported (DPARSF - >2mm threshold for exclusion), in addition to WM, CSF, and Global mean signal. | N/A | A-priori ROI based FC (Spearman), sliding window analysis (Pearson's R).  No atlas given; listed ROI's as defined by 8 papers: Tedeschi et al, Stankewitz, Moulton, Mickleborough, Chong, Li, Amin, and Maniya. |
| Guell et al., 2020 | **32 sports-related mTBI**  Age = 52.22±15.14y  Sex and time since injury not disclosed | - 3T - TR = 2400ms - 180 volumes - 7min 12sec - 3mm voxels (one dimension given) - Eyes closed | N/A | N/A | SPM12 w/ CONN+ART, global signal, CSF, and WM, and motion regressors. Motion threshold of 0.9mm (did not state needing to remove). | N/A | Whole-brain MVPA (w/ PCA), post-hoc seed based analysis. Data-driven parcellation (MVPA). |
| Lyer et al., 2020 | **62 paediatric mTBI**  27M/35F  Age = 14±2.43y  Time since injury not disclosed | - 3T - TR = 2000ms - 155 volumes - 5min 9sec - 3.6mm voxels (one dimension given) - Eyes open/closed not reported   2 scanning timepoints: roughly 4d apart (pre and during treatment). | N/A | N/A | Motion, CSF, and WM regressed. | GM and intracranial brain volumes regressed. | Whole-brain FC (Pearson, with network-based statistic approach), ReHo for intraregional FC.  Schaeffer parcellation. |
| Johnson et al., 2020 | **60 sport + non sport-related TBI (acute concussion)**  26M/34F  **30 TBI sport+**  13M/17F  Age = 19.4±2.6y  **30 TBI sport-**  13M/17F  Age = 23.1±3.8y  Time since injury not disclosed | - 3T - TR = 2000ms - 20000ms - 182 - AT = 6.067 - 3 × 3 × 5mm - 3.75 × 3.75 × 4.55mm voxels - "Eyes open fixation cross" | N/A | N/A | Motion regressed; as with CSF and WM. | Age. | Seed-based (a priori), averaged individual residualized time-courses using voxel-wise correlation maps (Pearson's).  3 networks based on Franco et al. 2009 parcellation. |
| Lewis et al., 2021 | **28 combat-related mTBI**  28M  Age = 34.4±7.5y  Time since injury = 56.1±31.9mo | - 3T - TR = 3000ms - 150 volumes - 7min 30sec - 1.7188 x 1.7188 x 3mm voxels - Eyes closed, awake | N/A | N/A | ANATICOR procedure to remove artifacts, including six motion parameters, localized average WM (within a 15-mm-radius sphere centered on each voxel) and CSF time-series, and respiration and cardiac signals | Depression. | Whole brain correlations 'connectedness' (Pearson's), seed-based for functional connectivity (cluster analysis with group-level T-tests).  Parcellation not disclosed. |
| Li et al., 2020 | **58 acute mTBI**  28M/30F  Age = 37.71±10.92y  Time since injury = < 7d (median= 3d) | - 3T - TR = 2000ms - 240 volumes - 8min - 4mm voxels (one dimension given) - Eyes not reported | N/A | Stated removal for two subjects due to 'abnormal intracranial findings from conventional MRI', no further details provided. | If movement direction is higher than 2.0°, excluded from this study.  Six head movement parameters and the average time series of global, cerebrospinal fluid and white matter signals were added into the regression analysis. | N/A | Seed based FC correlations (Pearson's) as well as whole brain FC between group analysis.  WFU PickAtlas (identification of seeds), DARTEL (parcellation using T1) parcellations. |
| Lu et al., 2019 | **58 mTBI**  33/25F  Age = 38.81±10.67y  Time since injury = 3.21±1.96d | - 3T - TR = 2000ms - 244 volumes - 8min 7sec - 4mm voxels (one dimension given) - Eyes open/closed not reported | N/A | N/A | Any participant who had a head motion greater than 3.0mm or a rotation in the x, y, or z directions higher than 3.0◦ were excluded. | N/A | Seed based (substrantia nigra as seeds) correlations (Pearson's) for FC.  WFU PickAtlas software (identification of seeds), AAL Atlas (whole-brain mask) parcellations. |
| Lu et al., 2022 | **76 mTBI**  34M/42F  Age = 43.79±10.22y  Time since injury not disclosed | - 3T - TR = 2000ms - 230 volumes - 7min 40sec - 4mm voxels (one dimension given) - Eyes open/closed not reported | N/A | N/A | Any subject with head motion greater than 2.0mm or a rotation in the x, y, or z directions higher than 2.0° were excluded. | N/A | Seed-based, network-level, static and dynamic functional network connectivity (Pearson's).  PCA, ICA parcellations using MATLAB's GIFT and ICASSO |
| Churchill et al., 2019 | **24 concussion**  11M/13F  Age = 20.0±1.9y  Time since injury = 1w | - 3T - TE = 30ms - TR = 2000ms - 3.125 x 3.125mm^2 in-plane resolution - 4mm slice thickness with 0.5mm gap - 195 time series - Lie still, eyes closed   3 scanning timepoints: 1w post injury, RTP + 1y post RTP. | N/A | N/A | Rigid-body motion correction (AFNI 3dvolreg). | SCAT3. | Voxel wise correlation, globe connectivity. |
| Churchill et al., 2020 | **61 acute phase concussion athletes**  30M/31F  Age = 20.4±2.0y  Time since injury = 4d (range = 1 – 6d) | - 3T - TE = 30ms - TR = 2000ms - 3.125 x 3.125mm^2 in-plane resolution - 4mm slice thickness with 0.5mm gap - 195 time series - Lie still, eyes closed - resampled at 3 × 3 × 3–mm3 resolution - Eyes open/closed not reported   5 scanning timepoints: 1-7d (acute), 8-14d (subacute), medical clearance RTS, 1mo post RTS, 1y post RTS. | N/A | N/A | Rigid-body motion correction (AFNI 3dvolreg).  Removal of outlier scan volumes through SPIKECOR algorithm (nitrc.org/projects/ spikecor).  Slice-timing correction (AFNI 3dTshift).  Spatial smoothing (6mm full width at half maximum (FWHM) isotropic 3D gaussian kernel (AFNI 3dmerge).  Regression of motion parameters and linear quadratic trends. | SCAT3. | Voxel wise correlation. |
| Churchill et al., 2021 | **26 concussion university athletes**  12M/14F  Age = 19.9±1.9y  Time since injury = 1w | - 3T - TE = 30ms - TR = 2000ms - 3.125 x 3.125mm^2 in-plane resolution - 4mm slice thickness with 0.5mm gap - 195 time series - Lie still, eyes closed - resampled at 3 × 3 × 3–mm3 resolution   3 scanning timepoints: ACU, RTP, 1y post RTP. | N/A | N/A | Rigid-body motion correction (AFNI 3dvolreg). | SCAT3. | Voxel wise correlation. |
| Champagne et al., 2019 | **23 sport-related concussion collegiate football players**  **14 SRC+**  Age = 21.0±1.0y  Time since injury = 5±3y (range = 2 – 10y)  No. previous concussions = 1.4±0.5 (range= 1 – 2)  9 SRC-  Age = 20.0±1.0y  Sex not disclosed | - 3T - TR = 4000ms - TE1/TE2 = 10/30ms - 9min - Eyes open fixated on cross | N/A | N/A | 1 removed due to excess motion. | N/A | Group spatial ICA (voxel-based network-wide correlations). |
| Botchway et al., 2022 | **35 mild/moderate TBI**  22M/13F  **20 mild complicated TBI**  13M/7F  Age = 10.5±1.85y  **15 moderate TBI**  10M/5F  Age = 9.94±1.40y | - 3T - TR = 2000ms - TE = 35ms - 3.3 x 3.3 x 3.3mm - 6min - 202 volumes - Eyes closed, think nothing | N/A | 1 removed due to extensive frontoparietal tissue damage. | Motion correction through MCFLIRT.  Volumes with a framewise displacement of >.5mm removed.  ICA-AROMA and nuisance regression used to remove motion artifacts. | N/A | Seed based analysis, ROI in cortex and subcortical structures, cortical ROI (automated anatomic labelling atlas), graph theory, global network organisation. |
| Bittencourt-Villalpando et al., 2021 | **54 subacute mTBI**  36M/18F  Age = 35y  Age range= 19 – 64y  Time since injury = 2w | - 3T - TR = 2000ms - TE = 20ms - 3.5 × 3.5 × 3.5mm - 10 min - 300 volumes - Eyes closed | N/A | N/A | Motion parameter. | Sex, motion correction for head movement, interval between the accident and the scan (in days), GCS score. | Group ICA, FNC correlation matrix. |
| Bittencourt et al., 2022 | **25 mTBI**  16M/9F  Age = 68±5y  Time since injury = 38±9d | - 3T - TR = 2000ms - TE = 9.74, 22.10, 34.46ms - 10min - 300 volumes - Eyes closed | N/A | N/A | N/A | Average framewise displacement, severity of complaints score (HISC-sev), age, group, interaction term group × HISC-sev. | Group ICA, SMI, FNC. |
| Banks et al., 2016 | **13 mTBI**  9M/4F  Age = 39.3±14.0y | - 3T - TR = 2000ms - TE = 35ms - 10min - 300 volumes - Eyes closed | N/A | N/A | Motion correction by SPM8 software. | N/A | ROI defined using WFU PickAtlas software. |
| Astafiev et al., 2016 | **20 chronic mTBI**  9M/11F  Age and time since injury not disclosed | - 3T - TR = 2000ms - TE = 27ms - Eyes open/closed not reported | N/A | N/A | Six parameters, 1 participant removed due to excess movement. | N/A | Seed analysis, ROI, voxelwise analysis. |
| Arciniega et al., 2021 | **23 mTBI**  13M/10F  Age = 22.3±3.4y  Time since injury = 4.2±3.6y (range= 5mo – 12.2y)  (Data from Experiment 3) | - 3T - TR = 2000ms - TE = 30ms - 155 volumes - Eyes closed | N/A | N/A | Six motion parameters. | N/A | Seed analysis, ROI generated manually with AFNI. |
| Amir et al., 2021 | **27 mTBI with persistent post-concussive symptoms**  8M/19F  Age = 43.9±10.2y  Time since injury = 8.8±6.0mo (range= 3.2 – 21.4mo) | - 3T - TR = 2250ms - TE = 30ms - 8min - 210 volumes - Eyes closed | N/A | Structural MRI assessed for abnormalities (subarachnoid haemorrhage, epidural and subdural haemorrhage, signs of diffuse axonal injury, any non-haemorrhagic contusion or encephalomalacia). | Band-pass filtered (motion regressed). | N/A | Seed to voxel analysis, ROI to ROI, Group ICA. |
| Yan et al., 2017 | **22 mTBI**  17M/5F  Age (median) = 23.5y  Age range = 18 – 60y  Time since injury = 3d | - 1.5T - TR = 2000ms - TE = 40ms - 6min 12s - Eyes closed | N/A | N/A | Head motion correction. | N/A | ROI, small world organization. |
| Xiong et al., 2016 | **25 chronic mTBI**  16M/9F  Age = 32.5 ± 10.4y  Time since injury not disclosed | - 3T - TR = 2000ms - TE = 50ms - 8min - Eyes closed, lights off | N/A | N/A | Head movement correction. | N/A | FC analysis. |
| Wang et al., 2021 | **42 mTBI**  23M/19F  Age = 37.4±11.9y  Time since injury = 14d | - 1.5T - 480 volumes - 16min - Eyes not reported | N/A | N/A | Motion correction FLIRT. | N/A | N/A |
| Wang et al., 2021 | **62 mTBI**  42M/20F  **30 original sample**  Age = 40.5±11.1y  **32 replicated sample**  Age = 36.8±11.3y | - 1.5T - Eyes closed | N/A | N/A | Motion correction FLIRT. | N/A | Graphy theory. |
| Vaughn et al., 2022 | **70 moderate/severe TBI children**  43M/27F  Age = 12.50±2.25y  Time since injury = 7w | - 3T - TR = 2000ms - TE = 30ms - 1.25 x 1.25 x 3.5mm - 180 volumes - 6min 3sec - Eyes closed | 104 lesions present in 48 patients. | N/A | Subject with motion outliers > 15% of volumes removed (n = 2). | N/A | N/A |
| Valera & Kucyi 2017 | **20 victims of DV**  20F  Age = 33.9±11.6y  Time since injury not disclosed | - 3T - TR = 3000ms - TE = 30ms - 3 x 3 x 3mm - 6min - Eyes open | N/A | N/A | Motion correction FLIRT, 6 motion parameters. | N/A | Seed based. |
| Trofimova et al., 2021 | **12 post-concussive vestibular dysfunction (PCVD)**  1M/11F  Age = 29.3±13.6y  Time since injury = 51±38d (range = 18 – 127d) | - 3T - TR = 2000ms - TE = 30ms - 3 x 3 x 3.5mm - 10min - Eyes open/closed not reported | N/A | N/A | N/A | N/A | Graph theory, group level ICA. |
| Sun et al., 2021 | **60 mTBI**  35M/25F  Age = 36.12±12.91y  Time since injury = 1w (median = 2d; range = 1 – 7d) | - 3T - TR = 2500ms - TE = 30ms - 180 volumes - 7min 30s - Eyes closed | N/A | No lesions found. | Head holder (head motion correction). | Years of education. | Group ICA, graph theory (Schrepf and colleagues). |
| Stevens et al., 2012 | **30 mild TBI**  20M/10F  Age = 31.7±13.9y  Time since injury = 60.9±35.77d | - 3T - TR = 1500ms - TE = 28ms - 210 time points - 5min 9sec - Eyes open fixated on cross | N/A | N/A | Head motion restricted by cusion. No excessive head motion, < 1 voxel length in any direction. | N/A | ICA |
| Stephenson et al., 2020 | **105 paediatric mild TBI**  58M/47F  Age = 14.04±2.7y | - 3T - TR = 460ms - TE = 29ms - 3.02 x 3.02 x 3mm - Eyes open/closed not reported | N/A | N/A | Foam padding.  Six motion parameters, their derivatives, average white matter, and cerebral spinal fluid (CSF) signals were regressed. | Age-at-injury, average of frame-by-frame translational and rotational head motion. | N/A |
| Spielberg et al., 2015 | **208 veterans**  **Time since injury not disclosed** | - Eyes open/closed not reported | N/A | N/A | N/A | N/A | Graph theory. |
| Zhan et al., 2015 | **15 mTBI**  6M/9F  Age = 38.5±11.5y  Time since injury = < 2w | - 3T - TR = 2000ms - TE = 30ms - 8min 6sec (first 6 sec consumed by dummy scan) - 240 volumes - Eyes closed | N/A | N/A | Participants with > 3mm of translation or > 3° rotation in any direction during the whole fMRI scan were excluded. | N/A | ReHo analysis. |
| Philippi et al., 2021 | **48 mTBI US servce members**  45M/3F  Age = 33.1±9.3y Age range = 19 – 59y  Time since injury = 317.9±193.9d | - 3T - Eyes open/closed not reported | 5 removed due to poor quality. | N/A | 2 removed due to excess motion. | N/A | Seed based voxelwise analysis. |
| Pagulayan et al., 2020 | **24 mTBI veterans (high-blast)**  24M  Age = 35.21±9.96y Age range = 22 – 61y  Time since injury = 73±26mo (range = 18 – 109mo) | - 3T - TR = 2000ms - TE = 24ms - 2 x 6min 30sec - Eyes open fixated on cross | N/A | N/A | Head immobilized, motion correction. Motion parameters, single point motion regressors. | PCL-M scores. | Seed based analysis. |
| Orr et al., 2016 | **16 athletes with remote history of concussion**  16M  Age range = 14 – 23y  Time since injury = 40.6mo (> 3mo) | - 3T - TR = 2000ms - TE = 35ms - 3.75 x 3.75 x 4mm - 10min - Eyes open/closed not reported | N/A | N/A | Six motion parameters. | Age. | ICA (post-hoc analysis). |
| Niu et al., 2019 | **70 mTBI**  45M/25F  Age = 34.7±12.2y  Time since injury = 2.0±2.3d | - 3T - 180 volumes - Eyes open/closed not reported | N/A | N/A | Six motion parameters. | N/A | PAG, seed based FC analysis. |
| Newsome et al., 2018 | **65 post-911 veterans**  53M/12F  Age = 42y  Age range = 26 – 59y  Time since injury (median) = 9.4y, (range = 0 – 44y) | - 3T - TR = 3000ms - TE = 30ms - 200 volumes - Eyes open | N/A | N/A | Motion parameters. Subjects with excessive motion in > 50% volumes were excluded. | N/A | Seed analysis. |
| Nathan et al., 2012 | **15 mTBI US military**  15M  Age = 25.6±4.4y  Time since injury = 143.3±85.4d | - TR = 2000ms - TE = 25ms - 3.75 x 3.75 x 4mm - 6min - Eyes closed | N/A | N/A | N/A | N/A | Graph theoretic analysis. |
| Nathan et al., 2015 | **15 mTBI active duty**  15M  Age = 25.6 ± 4.4y  Time since injury = 147.21±97.19d | - 3T - TR = 2000ms - TE = 25ms - 3.75 x 3.75 x 4mm - 6min - Eyes closed | N/A | N/A | 3 control subjects were excluded due to excessive head motion. | N/A | ICA group analysis. |
| Nathan et al., 2016 | **266 blast/non-blast mTBI**  **186 blast+**  183M/3F  Age = 32.4±7.3y  **80 blast-**  68M/12F  Age = 33.9±9.1y | - 3T - TR = 2000ms - TE = 25ms - 3.75 x 3.75 x 4mm - 6 min - Eyes closed | N/A | N/A | Motion parameters. | PTSS. | Graph theory. |
| Zhu et al., 2015 | **8 concussion patients**  8M  Age = 20±1.3y  Time since injury = 24hr | - 3T - TR = 2500ms - TE = 27.7ms - 2 x 7min - Eyes closed   3 scanning timepoints: 24hr, 7±1d + 30±1d after concussion | N/A | N/A | Rigid-body motion correction. Six motion parameters. | N/A | Network based FC analysis. |
| Nathan et al., 2017 | **56 mTBI low PTSS**  52M/4F  Age = 35.39±8.91y  **124 mTBI medium PTSS**  121M/3F  Age = 35.13±7.27y  **105 mTBI high PTSS**  103M/2F  Age = 34.05±6.64y | - 3T - TR = 2000ms - TE = 25ms - 3.75 x 3.75 x 4mm - 6min - Eyes closed | N/A | N/A | AFNI software for head movement correction. Motion parameters. | Age, years of education. | Group ICA. |
| Meier et al., 2017 | **49 mTBI**  49M  **25 mTBI** **concussion+**  25M  Age = 21.0±1.5y  **24 mTBI concussion-**  24M  Age = 20.2±1.2y | - 3T - TR = 2000ms - TE = 30ms - 6min - 180 volumes - Eyes open fixation cross | N/A | N/A | AFNI software. Six rigid-body motion parameters. | Age. | Seed-based analysis. |
| ***USC Papers- Phoebe*** | | | | | | | |
| ◊Wilde et al., 2019 | **11 paediatric TBI**  10M/1F  Age = 16.41±0.98y Age range = 15 – 18y  Time since injury = 33.1±1.7d | - 3T - TR = 3000ms - 200 volumes - 3.3mm isotropic - 10min - Eyes open fixated on a red circle in their line of sight | N/A | N/A | Head motion artifacts corrected (PRIDE). | N/A | N/A |
| ◊Wang et al., 2018 | **54 mTBI**  27M/27F  **27M**  Age = 35.4±9.7y  Age range= 19 – 54y  **27F**  Age = 35.6±9.4y  Age range = 21 – 54y  Time since injury = < 7d | - 3T - TR = 2000ms - 3mm isotropic - Keep eyes closed | N/A | Patients with lesions remained in study. | Head motion removed with multiple linear regression analysis.  Multi-resolution rigid body co-registration of volumes (MCFLIRT). | N/A | N/A |
| ◊Walton et al., 2022 | **48 former collegiate football players**  48M  Age range = 36 – 41y  Time since injury not disclosed | UNC MRIs (n = 38)   - TR = 802ms - 7min - 512 volumes - 2 x 2.1 x 2.1mm   Medical College of Wisconsin Pre-Update (n = 4)   - TR = 802ms - 6min 51sec - 512 volumes - 2 x 2.1 x 2.1mm   Medical College of Wisconsin post update (n = 6)   - TR = 800ms - 6min 50sec - 512 volumes - 1.5 x 2.08 x 2.08mm - Eyes open/closed not reported for any scan | N/A | N/A | 24 motion parameters were removed using a linear regression model.  FD-DVARS “scrubbing” approach was applied to further reduce motion effect.  Nuisance regression (mean signal from white matter, CSF, whole brain, and 24 motion parameters). | Age, BMI, MRI acquisition site. | N/A |
| Vergara et al 2018 | **48 mTBI patients**  23M/25F  Age = 22.9±9.18y  Time since injury = 14±5.3d | - 3T - TR = 2000ms - 5min - 3.75 x 3.75 x 4.55mm - 150 volumes - Eyes open fixated on a foveally presented cross | ICASSO (integrated in GIFT) was utilized to assess the quality of the resting state networks.  4 subjects were excluded due to high movement variance. | N/A | Artifactual motion components were detected and discarded. | Sex, age, education, WTAR score. | N/A |
| Vergara et al., 2017 | **50 mTBI patients**  25M/25F  Age = 27.9±9.2y  Time since injury not disclosed | - 3T - TR = 2000ms - 5min - 3.75 x 3.75 x 4.55mm - 145 volumes - Eyes open fixated on foveally presented cross | Artifactual RSNs were detected and discarded based on their frequency content. | N/A | Spike and motion correction. | Diagnosis, gender, age, translations, rotations, detected spikes. | N/A |
| ◊Vergara et al., 2017 | **50 mTBI patients**  25M/25F  Age = 27.3±9.0y  Time since injury not disclosed | - 3T - 5min - 3.75 x 3.75 x 4.55mm - 145 volumes - Eyes open fixated on foveally presented cross (stare passively) | Four subjects were excluded due to increased head motion. | N/A | Spike and motion correction. | N/A | N/A |
| ◊Vaninetti et al., 2021 | **26 mTBI with persistent headaches**  20M/6F  Age = 33±8y | - 3T - 5min - TR = 2000ms - Eyes open/closed not reported | N/A | N/A | N/A | N/A | N/A |
| ◊van der Horn et al., 2020 | **68 mTBI**  51M/17F  **20 incomplete recovery**  12M/8F  Age = 44.3y  Age range = 22 – 61y)  37 complete recovery  28M/9F  Age = 35.1y  Age range= 19 – 64y) | - 3T - TR = 2000ms - 3.5 x 3.5 x 3.5mm - 300 volumes - Eyes closed   2 scanning points: 37.2d + 96.5d post injury | 2 excluded for extreme head movement. | 9 mTBI had lesions on CT. Comparison of patients with and without lesions. | Extreme head movement defined as >3 SD on at least 2/6 of the mean framewise displacement measures. | Age, education. | N/A |
| ◊van der Horn et al., 2017 | **49 mTBI**  33M/16F  Age = 31y  Age range = 19 – 62y  Time since injury = 32d (range = 22 – 69d) | - 3T - TR = 2000ms - 3.5 x 3.5 x 3.5mm - 300 volumes - 10min - Eyes closed   2 scanning timepoints = 32d (22 – 69d) + 92d (61 – 207d) post injury | N/A | Scans were assessed for traumatic lesions. | 10 artifact components were discarded. | Age. | N/A |
| ◊van der Horn et al., 2016 | **54 mTBI +/- complaints**  36M/18F  Age = 35y  Age range = 19 – 64y  Time since injury = 33d (range = 22 – 69d) | - 3T - TR = 2000ms - 3.5 x 3.62 x 3.5mm - 300 volumes - Eyes closed | N/A | Patients with lesions were excluded (CT). | Images were realigned to correct for head motion. | HADS-A and HADS-D. | N/A |
| Vakhtin et al., 2013 | **13 mTBI blast veterans**  13M  Age = 34.3±6.6y  Time since injury not disclosed | - 3T - TR = 2000ms - 3.8 x 3.8 x 3.5mm - 5min 34sec - 167 volumes (150 analyzed) - Eyes open fixated on cross | Subjects whose average translation exceeded one voxel (3 mm) excluded (*N*= 0). | N/A | Largest average translation 2.10mm and the largest degree of rotation 1.02 degrees. | Age, translation in log(mm), rotation log(degrees). | N/A |
| Stephens et al., 2018 | **11 mTBI + moderateTBI in children**  6M/5F  Age = 16.0y  Age range = 12.6 – 18.7y  Time since injury = 381d (range = 364 – 403d)  **6 mTBI**  **5 moderateTBI** | - 3T - TR= 2500ms - 6min 30sec - Eyes open fixated on cross | One participant removed due to motion (>3mm displacement). | N/A | Nuisance regression (WM, ventricles, six motion parameters). | N/A | Seed maps – DMN. ROI analysis with Brodmann’s Area atlas. |
| ◊Sours et al., 2013 | **23 mTBI patients with/without memory complaints**  11M/12F  Age = 39.5±16.4y  Time since injury = 36d | - 3T - TR = 2000ms - 171 volumes - 5min 42sec - Eyes closed | Visual inspection of RSNs. | N/A | 6 motion correction parameters. Nuisance regression (CSF, WM). | N/A | FC (CONN). ROI - DMN, TPN, SN. |
| ◊Sours et al., 2015 | **41 mTBI**  30M/11F  Age = 43.7±17.0y  Time since injury = 7.7±2.4d | - 3T - TR = 2000ms - 171 volumes - 5min 42s - Eyes not reported | Motion parameters statistically compared between timepoints. 1 participant excluded due to head motion. | N/A | 6 motion correction parameters. Nuisance regression (CSF, WM, motion parameters). | N/A | FC (CONN), interhemispheric ROI analysis. |
| ◊Sours et al., 2015 | **77 acute mTBI**  59M/18F  Age = 44.0±17.0y  Time since injury = 6±3d | - 3T - TR= 2000ms - 5min 42sec - 171 volumes - Eyes not reported | N/A | N/A | 6 motion correction parameters. Nuisance regression (CSF, WM, motion parameters). | Age. | FC (CONN),  ROI analysis - Oxford thalamic connectivity atlas. |
| Sours et al., 2015 | **32 mTBI patients**  21M/11F  Age = 41.7±17.1y  Time since injury = 6d (range = 1 – 11) | - 3T - TR = 2000ms - 5min 32sec - 171 volumes - Eyes closed   2 scanning timepoints: < 10d + 6mo | Visual inspection of ROIs. | N/A | 6 motion correction parameters. Nuisance regression (motion parameters and global BOLD signal). | Age, 2 of the 6 motion parameters (x and roll). | DMN ROI analysis, Wavelet connectivity - strength and diversity. |
| ◊Smith et al., 2022 | **18 post-concussive vestibular dysfunction patients**  6M/12F  Age = 23.43±4.90y  Time since injury = 35.5± 29.0d | - 3T - TR = 750ms - 600 volumes - 7min 30sec - 2.5mm isotropic - Eyes not reported | Manual inspection of framewise displacement and TRs. | N/A | TR outliers, mean CSF and WM signal regressed out.  Groupwise mean and maximal motion examined. | Days elapsed since injury, age. | Seed to voxel and ROI to ROI connectivity (CONN). |
| ◊Slobounov et al., 2017 | **18 repetitive concussion in athletes**  18M  Age = 21.6±1.28y)  Time since injury not disclosed | - 3T - TR= 2000ms - 2 x 2 x 2mm - 300 volumes - Eyes closed | 1 subject removed due to motion. | N/A | Voxel outliers > 10% or normalised motion derivative > 0.5 removed.  3dDeconvolve (AFNI) to remove motion contribution.  Rigid body motion correction. | N/A | Yeo 17 networks, mean correlation of all node pairs, 3dfim+ (AFNI) seed regions. |
| Slobounov et al., 2011 | **17 sports related mTBI (student athletes)**  11M/6F  Age = 21.3±1.5y  Time since injury = 10±2d | - 3T - TR = 2000ms - 3.1 x 3.1 x 5mm - 6min 4s - Obtained at rest, stress, and recovery - Eyes open/closed not reported | N/A | No lesions of hyperintense signals present. | WM, CSF, physiological signals, motion, taken as confounds (CompCor). | Heart rate, motion artifact. | ICA, Brodmann Area atlas, ROI analysis. |
| Sheth et al., 2021 | **49 mTBI**  44M/5F  Age = 38y  Age range = 20 – 54y  Time since injury = 144.7±126.4mo  Time since most severe injury = 186.9±136.9mo  No. of TBI = 2.1±1.7 | - 3T - TR = 2000ms - 8min - 240 volumes - Eyes open | Significant differences in framewise displacement statistically compared between groups. | N/A | Minimum of 0.5 FD was allowed.  Rigid body motion correction.  Nuisance regression (6 head motion parameters, global signal, CSF, WM). | CAPS score, age, gender, education. | ROIs - left and right ACC from AAL atlas.  Seed-based whole brain correlation. |
| ◊Sharma et al., 2022 | **14 concussion children**  6M/8F  Age = 14.54±2.39y  Time since injury = 15.3±6.7d | - 3T - TR = 2000ms - 300 timepoints - 10min - Eyes open - Fieldmap acquired | Outlier scans exceeding 0.9mm FD or BOLD fluctuations > 5SD flagged (ART). | N/A | Nuisance regression of CSF, WM, outlier scans, subject motion (aCompCor). | Cardiorespiratory fitness (in adults). | Intra-network connectivity of DMN, SMN, SN, FPN. |
| Shapiro et al., 2021 | **41 concussion children**  29M/12F  Age = 13.08±2.62y  Age range = 5 – 18y  Time since injury = 2w | - 3T - TR = 1500ms - 6min 33sec - 2.5 x 2.5 x 2.5mm - Eyes open/closed not reported | Component maps visually inspected. | None had lesions based on visual inspection. | Motion correction (MCFLIRT). | N/A | ICA (MELODIC). Dual regression (FSL). |
| Shafi et al., 2020 | **80 concussion participants**  47M/33F  **47M**  Age = 32±13y  Time since injury = 18.79±21.28mo  **33F**  Age = 31.8±13.1y Time since injury = 20.70±29.37mo | - 3T - TR = 2500ms - 3.5 x3.5 x3.5mm - Eyes closed | ART based functional outlier detection. | N/A | Nuisance regression of CSF, WM, subject motion (aCompCor). | Age, sex, group (concussed vs control). | Within/between network FC and network to ROI FC.  DMN, SN, and FPN.  CONN default parcellation scheme (133 ROIs). |
| Robinson et al., 2015 | **134 veterans**  114M/20F  Age = 33.0±8.6y Age range = 19 – 62y  Time since injury = not disclosed | - 3T - TR = 3000ms - 3 x3 x3.75mm - 6min - Eyes open | 13 participants excluded due to motion. | N/A | Motion regressed out (FSFAST). | Age, gender, WTAR IQ, CAPS score. | Vertex-wise partial correlation to seed region. |
| Robinson et. al., 2017 | **269 TBI veterans**  241M/20F  Age = 31.9±8.3y)  **116 CBE+, 153 CBE-**  Time since injury = not disclosed | - 3T - TR = 3000ms - 3 x 3 x 3.75mm - 6min - 120 volumes - eyes open, awake | Poor MRI data (n = 76), incomplete covariates obtained (n = 34). | N/A | Motion correction (FSL/AFNI).  Global signal regression. | Clinician administered PTSD scale, age, gender, IQ. | Seed based (DMN, DAN). Correlation distribution analysis. |
| Rangaprakash et al., 2017 | 42 mTBI soldiers  42M  Age = 33.7±6.8y  Time since injury not disclosed | - 3T - TR = 600ms - 3 x 3 x 4mm - 1000 volumes - Eyes open fixated on cross | N/A | N/A | Nuisance regression (head motion, CSF, WM). | Age. | Seed based FC using ROI. |
| Rangaprakash et al., 2018 | **42 military**  42M  Age = 33.7±6.8y  Time since injury = 3mo − 5y | - 3T - TR = 600ms - 3 x 3 x 4mm - 1000 volumes - Eyes open fixated on cross | N/A | N/A | Nuisance regression (head motion, CSF, WM).  Max motion allowed was 1.5mm. | N/A | Effective connectivity analysis, HRF, behavioral relevance of connectivity values, classification using SVM. |
| ◊Rangaprakash et al., 2017 | **42 mTBI soldiers**  42M  Age = 33.7±6.8y | - 3T - TR = 600ms - 3 x 3 x 4mm - 1000 volumes - Eyes open fixated on cross | Visual inspection, partial brain coverage (n = 3), excess motion (n = 2). | N/A | Nuisance regression (head motion, CSF, WM).  Max motion allowed was 1.5mm. | N/A | Multivariate N-way ANOVA statistical test, regrouping based on connectivity. |
| Rajesh et al., 2017 | **22 mTBI**  Age = 36.16±16.35y  **12 mTBI 1 – 10y post injury**  5M/7F  **10 mTBI 20 – 65y post injury**  6M/4F | - 3T - TR = 2000ms - 3.4 x 3.4 x 4.0mm - 2 x 2min 20sec - Eyes open/closed not reported | Visual inspection of signal and noise components by two raters. | N/A | Motion correction (AFNI *3dvolreg*). Nuisance regression (head motion, WM, CSF). | Time since injury. | ICA (MELODIC), ROI analysis, seed-based. |
| ◊Peters et al., 2020 | **24 NFL players with history of concussion**  24M  Age (median)= 37.5y  Median 4 concussions | - 3T - TR = 2000ms - 210 volumes, - Eyes closed, keep still, awake | ArtRepair toolbox for excessive motion and global signal noise. | N/A | Motion correction (SPM12).  ArtRepair toolbox for excessive motion and global signal noise.  Nuisance regression (head motion, CSF, WM) (CompCor). | N/A | ROIs chosen based on PET imaging. |
| Palacios et al., 2017 | **75 mTBI**  Age range = 18 – 55y  Time since injury = 11.2±3.3d  Sex not disclosed | - 3T - TR = 2000ms - 7min - 3.4 x 3.4 x 4mm - Eyes closed | 4 subjects excluded due to rsfMRI artifacts. | N/A | Motion correction (Jenkinson et al., 2002). | N/A | FSLNETs for connectivity matrices.  Probabilistic ICA. |
| Nordin et al., 2016 | **10 chronic mTBI**  5M/5F  Age = 37.5±11.2y  Time since injury = 0.5 – 19y | - 3T - TR = 2000ms - 8min - 240 volumes - Eyes not reported | N/A | N/A | Rigid body registration.  Nuisance regression (6 motion parameters, CSF). | N/A | ROI, ICA, QDA, connectivity count index, connectivity strength index. |
| ◊Murdaugh et al., 2018 | **16 high school football players with SRC**  16M  Age = 15.99±1.18y Age range = 14 – 18y  Time since injury = < 7d | - 3T - TR = 2130ms - 3 x 3 x 3mm - 192 volumes - 7min - Eyes open/closed not reported   2 scanning timepoints: <7d + 21d post injury | No subjects excluded. | N/A | Motion corrected (SPM12). <10% of consecutive volumes had >0.2 mm movement.  Spike regression. | N/A | Group ICA (ICASSO).  GIFT, ICA toolbox.  Correlation w ImPACT scores. |
| ◊Muller and Virji-Babul, 2018 | **6 sports-related concussion adolescent athletes**  Age = 15.5y  Time since injury = > 2mo  Sex not disclosed | - 3T - TR = 2000ms - 3 x 3 x 3mm - 240 volumes - Eyes open fixated on cross | Subjects with 5% outliers over the 230 functional volumes excluded. | N/A | No displacement > 1 mm in 3 translation and 3 motion (at 50mm radius).  ART (scan-to-scan global signal z value threshold = 3,  scan-to-scan motion threshold = 0.5 mm). | N/A | GIFT ICA.  Graph theory (AAL atlas) - rich club, strength, betweenness centrality, nodal strength. |
| ◊Militana et al., 2016 | **7 college athletes SRC**  4M/3F  Age = 19.7±1.2y  Time since injury = 3 – 6d | - 3T - TR = 2000ms - 300 volumes - 10min - Eyes open/closed not reported | N/A | N/A | Motion correction (SPM8).  Nuisance regression (motion parameters, WM) | Cerebrovascular reactivity. | 18 ROIs (DMN, DAN, FPN). |
| ◊Messe et al., 2013 | **55 mTBI patients +/- PCS**  37M/18F  Age = 34.9±11.5y  Time since injury = 8 – 21d | - 3T - TR = 2650ms - 180 volumes - 10min - 1.5 x 1.5 x 2mm - Eyes closed   2 scanning timepoints: 8 – 12d + 6mo | 6 excluded due excessive head motion (greater than 3 mm or 3 degrees in any dimension), or MR artifacts. | N/A | Nuisance regression (motion parameters). | N/A | Functional brain networks.  Graph theory (AAL, BCT) - graph cost, nodal strength, diversity. |
| Meier et al., 2020 | **92 mTBI + concussion**  18M/14F  Age = 19.20±0.90y  Time since injury = 24 – 28hr | - 3T - 10mins - 3.5 x 3.5 x 3.5mm - Eyes open   GE MR750:   - TR = 2000ms - 300 volumes   Siemens Trio:   - TR = 2250ms - 267 volumes   Siemens Prisma:   - TR = 2300ms - 261 volumes | Visually inspected for artifacts.  Scans with Euclidean norm of motion > 0.2 or more than 30% of time-points were excluded. | N/A | Euclidean norm of motion > 0.30 were censored.  Nuisance regression (6 motion parameters and derivatives, zero through third-order polynomial trends, CSF, WM). | N/A | Graph theory (whole-brain Craddock r 2-level 200 region, BRAPH) - nodal strength. |
| Meier et al., 2021 | **37 mTBI**  **16 mTBI concussion-**  16M  Age = 17.81±1.83y  **21 mTBI concussion+**  21M  Age = 18.05±1.75y  Time since injury = 1, 8, 15, & 45d | - 3T - TR = 720ms - 501 volumes - 6min - 2 x 2 x 2.5mm - Eyes open | Scans in which the average Euclidean norm of motion > 0.2 were excluded. | N/A | Euclidean norm of motion >0.30 were removed along with the preceding volume.  Nuisance regression (6 motion parameters and derivatives, zero through third-order polynomial trends, CSF, WM). | N/A | FC: AFNI 3dNetCorr, AAL atlas and Craddock parcellation.  Graph Theory Software (BRAPH) - nodal strength. |
| ◊McCuddy et al., 2018 | **43 mTBI concussion**  34M/9F  Age = 20.29±1.31y  Time since injury = 1d | - 3T - TR = 2000ms - 180 volumes - 6min - 2.5 x 2.5 x 3.2mm - Eyes open fixated on cross   3 scanning timepoints: 1d, 1w + 1mo | 15 athletes excluded: <128 usable volumes following motion censoring, poor scan quality, or excessive head motion. | N/A | Euclidean norm of motion > 0.30mm were censored.  Six rigid-body motion parameters and their derivatives regressed out. | N/A | Functional Connectivity with HAM-D score.  Functional Connectivity of DMN.  Yeo Parcellation Scheme. |
| ◊Mayer et al. 2019 | **50 paediatric mTBI**  30M/20F  Age = 15.73±2.14y Age range = 12 – 18y  Time since injury = 1w | - 3T - 3mm^3^ - 647 volumes - TR = 460ms - Eyes open/closed not reported   2 scanning timepoints: 1w + 4mo | N/A | N/A | 12 motion parameters.  AFNI two- and three- dimensional motion correction.  Nuisance regression (motion, CSF, WM). | Retrospective ratings, mean FD. | FC vs CBF, HRF. |
| ◊Mayer et al., 2011 | **27 mTBI**  12M/15F  Age = 27.15±7.38y  Time since injury = < 3w | - 3T - TR = 2000ms - 3.75 x 3.75 x 4.55mm - 150 volumes - 5min - Eyes open/closed not reported | Group effect head motion – not significant, increased motion in mTBI group. | N/A | Nuisance parameters (head motion, CSF, WM, linear trends). | WTAR. | Seed-based analysis, network motif. |
| Mayer et al., 2015 | **48 semi-acute mTBI**  23M/25F  Age = 28.3±9.5y  Time since injury = 14d | - 3T - TR = 2000ms - 152 volumes - 5 mins - 2.5 x 2.5 x 3.2mm - Eyes not reported | N/A | N/A | 6 motion parameters.  Outliers above 3 standard deviations of the mean head movement excluded. | WTAR, mean FD, premorbid intelligence. | Functional Connectivity, ICA, PCA, seed-based analysis. |
| ◊Manning et al., 2017 | **17 mTBI/concussed athletes**  17M  Age = 13.3±0.6y  Age range = 11 – 14y  Time since injury = 24 – 72hr | 2 scanning timepoints: 24-72hr and 3mo post injury | N/A | N/A | Data >1-mm max displacement or >0.5-mm relative mean displacement excluded. | N/A | ICA, seed-based FC.  Harvard-Oxford Parcellation |
| Luo et al., 2021 | **24 mTBI patients** 15M/9F  Age = 38.88±13.33y  Time since injury = 3.58±3.28d | - 3T - TR = 2000ms - 250 volumes - Eyes open/closed not reported | 4 subjects excluded (> 1.5 degree of motion rotation and/or 1.5 mm maximum translation in x, y, or z). | N/A | Head motion correction (DPABI).  Head motion effect was regressed out (Friston 24). | N/A | FC binary classification.  AAL atlas |
| Lu et al., 2020 | **44 mTBI post traumaticheadache+**  17M/27F  Age = 40.9±10.9y  **27 mTBI post traumatic headache-**  14M/13F  Age = 40.5±10.6y  Time since injury not disclosed | - 3T - TR = 2000ms - 8min 8sec - 240 volumes - Eyes open/closed not reported | Any participant with motion >3.0 mm or a rotation >3.0° were excluded. | Lesions observed. | Motion correction (SPM, REST). | N/A | Seed-based FC.  ROI left and right hypothalamus. |
| Lu et al., 2020 | **53 mTBI**  27M/26F  Age = 37.96±10.708y  Time since injury = 2.95±1.611d | - 3T - TR = 2000ms - 8min 8sec - 240 volumes - Eyes open/closed not reported | Any participant with motion >3.0 mm or a rotation >3.0° were excluded. | N/A | Motion correction (SPM, REST). | N/A | Seed-based FC.  ROI insula. |
| Li et al., 2020 | **50 mTBI**  24M/26F  Age = 43.82±7.88y  Time since injury = < 7d | - 3T - TR = 2000ms - 243 volumes - 8min 6sec - Eyes closed | 3 participants excluded (movement >2.0 mm translation or >2.0° rotation). | N/A | Motion correction (DBAPI).  Nuisance regression (head motion, WM, CSF, whole brain). | Age, sex, education level. | Group ICA, 13 RSNs, Static FC. |
| Li et al., 2019 | **55 mTBI patients**  27M/28F  Age = 40.65±11.44y  Time since injury = 3.47d (0 – 7d) | - 3T - TR = 2000ms - 8min 6sec - Eyes closed | 6 subjects excluded (> 2.0° of rotation or > 2.0 mm of translation). | N/A | Motion correction (DBAPI).  Nuisance regression (head motion, WM, CSF, whole brain). | Age, sex, education. | Granger causality analysis, ICA, seed-based analysis, degree centrality. |
| Leung et al., 2016 | **15 mTBI**  **11M**  Age = 34.6±8.7y Age range = 25 – 54y  **4F**  Age = 31.2±4.0y Age range = 27 – 36y  Time since injury = 30min | - 1.5T - TR = 2000ms - 150 volumes - 5min - Eyes open/closed not reported | No patients were excluded because of excessive head motion. | N/A | Motion correction (Brain Voyager). | N/A | SogICA, Granger causality analysis, seed-based ROIs. |
| Lemme et al., 2021 | **21 mTBI persistent PTH**  7M/14F  Age = 16.18±2.0y  Time since injury = 106.62±22.4  **18 mTBI resolved PTH**  7M/11F  Age = 17.07±2.9y  Time since injury = 113.22±29.5 | - 3T - TR = 1100ms - 320 volumes - 6min - 3mm^3^ - Eyes open | 15 athletes with < 128 usable volumes following motion censoring were excluded. | N/A | Motion > 0.90mm and rotation >1deg were censored. | N/A | ROI (CONN), AAL atlas, seed-based analysis. |
| ◊Kuceyeski et al., 2019 | **51 mTBI**  35M/16F  Age = 29.6±8.6y  Time since injury = 1w | - 3T - TR = 2000ms - 7min - 3.4 × 3.4 × 4mm - 210 volumes - Eyes open/closed not reported   4 scanning timepoints: 1w, 1mo, 6mo, and 12mo post injury. | Removal of outlier volumes (CONN). | N/A | 24 motion covariates (CompCor). | N/A | Graph analysis (BCT, Desikan-Killianey atlas) - degree, characteristic path length, global and local efficiency, clustering coefficient, modularity, small-worldness, transitivity, mean first passage time, and mean navigation time. |
| ◊Kim et al., 2022 | **29 mTBI**  14M/15F  Age = 43.3±14.5y  Time since injury = 1mo | - 3T - TR = 3500ms - 116 volumes - 1.9 × 1.9 × 3.5mm - Eyes closed | Estimated each subject’s movement (FD). Mean FD comparison between groups. | Micro-bleeds, nonspecific T2 hyperintensities, or negligible subdural/epidural haemorrhage included. | Motion-related artifacts removal using ICA-AROMA (Pruim et al., 2015). Nuisance regression (WM, CSF). | Age, sex. | Graph analysis (BCT, AAL atlas) - betweenness centrality, strength, clustering coefficient, local and global efficiency. |
| ◊Killgore et al., 2020  (702)  (Risha) | **32 mTBI**  15M/17F  Age = 23.2±7.2y Age range = 18 – 48y  Time since injury = 6.8±4.0mo | - 3T - 6min - TR = 2000ms - 2mm^3^ - Eyes open/closed not reported | 2 subjects removed (> 20% of volumes were identified as outliers, ART toolbox). | N/A | Outlier images with movement exceeding 0.5mm or a global mean intensity > 3 SD.  Nuisance regression (first image, outliers, CSF, WM) (CompCor). | Age, intracranial volume, number of days using the light device. | Seed-to-voxel FC (CONN), Seed-to-ROI, Directed FC (Granger causality). |
| ◊Kaushal et al., 2019 | **62 high school/college athletes**  Time since injury = < 48hr acute  Age/sex not disclosed | - 3T - TR = 720ms - 501 volumes - Eyes open   4 scanning timepoints: < 48hr acute + 8d, 15d + 45d (sub-acute) | Visual inspection for artifacts.  Scans were average Euclidian norm of motion parameters was > 0.2 excluded. | N/A | Nuisance regression (CSF, WM, motion parameters and derivatives). | N/A | ROI analysis (Craddock whole-brain functional parcellation atlas).  Graph theory (BCT, BRAPH) – strength. |
| Johnson et al., 2012 | 23 sports concussion  5M/18F  Age = 20.6±1.2y  Time since injury = < 24hr | - 3T - TR = 2000ms - 3.1 x 3.1 x 5mm - Eyes closed - 6min 4sec - 182 volumes   2 scanning timepoints: < 24 hours + 10±2 days | N/A | No radiological findings such as lesions were present. | Nuisance regression (WM, CSF, motion, physiological noise, whole brain BOLD signal). | N/A | Voxel-based correlation (CONN).  ROI-based correlation (Brodmann area atlas). |
| Johnson et al., 2014 | **12 subconcussive sports injuries**  5M/7F  Age = 20.5y  Age range = 19 – 23y  Time since injury = 18.8mo (range = 1 – 48mo) | - 3T3.0mm^3^ voxels - TR = 2490ms - 5min 4sec - 122 volumes - Eyes open/closed not reported | N/A | N/A | Nuisance regression (motion, WM, CSF, physiological noise, whole brain BOLD signal) (CompCor). | N/A | Seed-based correlation analysis of the DMN (CONN).  ROIs based on Brodmann Areas. |
| ◊Jia et al., 2021 | **97 acute mTBI**  48M/49F  Age = 38.99±13.76y  Time since injury = < 7d  **56 chronic mTBI**  33M/23F  Age = 35.32±15.32y  Time since injury = 6 – 12mo | - 3T - TR = 2500ms - 3mm^3^ voxels - 180 volumes - Eyes closed | 16 subjects excluded for head motion exceeding 2mm and/or 2° rotation. | Participants with intracranial lesions not requiring surgery included. | Nuisance regression (motion parameters, CSF, linear trend signal, FD). | Age. | White-matter Functional Networks. |
| Iyer et al., 2019 | **62 children with persistent post concussive symptoms (PPCS)**  27M/35F  Age range= 8.5 − 17.9y  Time since injury = 4 – 6w | - 3T - TR = 2000ms - 5min 10sec - 155 volumes - Eyes open/closed not reported | 10 subjects excluded with less than 95% of data remaining after the removal of contaminated volumes (FD > 0.4 mm). | N/A | Nuisance regression (head motion, linear trends, CSF, WM). | N/A | Machine learning (SVM classifier), intra–regional functional connectivity (KCC), Seed-based interregional FC. |
| Iyer et al., 2019 | **100 children with persistent post concussive symptoms (PPCS)**  45M/55F  Age = 14.06±2.06y  Time since injury = 4w | - 3T - TR = 2000ms - 150 volumes - 5min 10sec - Eyes open/closed not reported | 10 children were excluded following neuroimaging data quality control. | N/A | Regression of nuisance covariates (linear trends, CSF, WM) (Friston 24). | N/A | Schaefer parcellation (214 regions), CCA connectivity analysis. |
| Irimia et al., 2020 | **29 geriatric mTBI**  16M/13F  Age = 68±6y  Age range = 57 – 79y  Time since injury = 5.6±0.5mo | - 3T - TR = 3000ms - 140 volumes - Eyes open/closed not reported | N/A | N/A | FS-FAST motion correction.  Nuisance regression (CSF, WM, motion parameters). | N/A | FC (FS atlas) seed based analysis), graph analysis (BCT), multivariate regression analysis. |
| ◊Iraji et al., 2016 | **16 mTBI**  10M/6F  Age = 34.52±13.85y  Age range= 19 – 63y  Time since injury = 82.64±121.90d | - 3T - TR = 2000ms - 3.125 x 3.125 x 3.5mm - 240 volumes - Eyes closed   2 scanning timepoiunts: 82.64±121.90d after initial injury + 42.68±17.48d after first scan | N/A | N/A | Motion correction (MedINRIA). | N/A | FC (DICCCOLs), graph theory (NBS). |
| ◊Iraji et al. 2015 | **12 mTBI**  6M/6F  Age = 38.0±17.0y Age range = 19 – 73y  Time since injury not disclosed | - 3T - TR = 2000ms - 3.125 x 3.125 x 3.125mm - 240 volumes - 8min - Eyes open/closed not reported | 3 patients removed due to motion. | N/A | Motion correction (FSL).  Nuisance regression (WM, CSF) (DPARSF). | N/A | Group, individual, and atlas-based ICA, seed-based analysis. |
| Hou et al., 2019  (720)  (Phoebe) | **47 acute mTBI**  34M/13F  Age = 41.7±17.3y  Time since injury = < 10d | - 3T - TR = 2000ms - 171 volumes - 5min 42sec - eyes closed | N/A | N/A | Nuisance regression (6 motion parameters, average BOLD signal, WM, CSF). | N/A | ICA, static and dynamic FC.  Graph analysis - characteristic path length, clustering coefficient, and minimum spanning trees. |
| Hogeveen et al., 2021 | **70 subacute mild + chronic mild-moderate TBI**  45M/25F  **44 subacute mild TBI**  29M/15F  Age = 28.1±9.61y  **26 chronic mild-moderate TBI**  16M/10F  Age = 32.6±12.0y  Time since injury = 3mo to 15y | - 3T - TR = 460ms - 3mm iso - Eyes open/closed not reported | 4 subjects removed due to excessive head motion during MRI. | N/A | Motion outliers defined by fMRIprep removed.  Nuisance regression (head motion, FD, DVARS, CSF, WM, physiological noise) (CompCor). | Time since injury, injury severity, disinhibition, executive dysfunction, anxiety, depression, fatigability. | Seed-to-voxel functional connectivity (CONN). |
| Hayes et al., 2018 | **110 combat veterans**  102M/8F  Age = 30.14±7.7y  Time since injury not disclosed | - 3T - TR = 3000ms - 3 x 3 x 3.75mm - 2 x 6min - 2 x 120 volumes - Eyes open | Visual examination. | N/A | Motion correction (FS-FAST).  Nuisance regression (motion). | Age, lifetime substance use, premorbid IQ, education. | ROI analysis (PCC, AG, MTG, mPFC). |
| Han et al., 2014 | **103 US military personnel**  100M/3F  Age = 24y  Age range= 19 – 57y  Time since injury = < 90d (median = 1 – 2w) | - 1.5T - TR = 2500ms - 3 x 6min 52sec (~20 mins total) - 165 volumes - remain still (no eye preference) | Visual inspection at every step of preprocessing.  25 subjects excluded (motion correction failure, intensity distortion, susceptibility artefacts, lack of frames/data after scrubbing). | N/A | Standard motion correction.  Nuisance regression (motion parameter, CSF, WM, global signal). | N/A | Graph theoretical analysis (BCT, Destrieux atlas) - modularity, strength, participation coefficient.  ROI analysis, multivariate. |
| ◊Goswami et al., 2016 | **19 self-reported multiple concussion (retired athletes)**  19M  Age = 50±12y  Age range = 30 – 74y  Number of self-reported concussions = 2 – 15 | - 3T - TR = 2000ms - 3.125 x 3.125 x 4mm - 5min 8sec - 154 volumes - Eyes closed | N/A | N/A | Motion correction (mcFLIRT).  Nuisance regression (motion parameters, WM, CSF) (CompCor). | N/A | FC between ATL and OFC, machine learning. |
| Gilmore et al., 2016 | 127 veterans  117M/10F  Age = 32.7±8.1y  Time since injury = 1 – 5y | - 3T - TR = 2000ms - 3.4 x 3.4 x 4.0 - 6min - 180 volumes - Eyes closed - field map acquired | N/A | N/A | Motion correction (FLIRT). | N/A | Seed to ROI FC. |
| Flowers et al., 2021 | **48 mTBI with related headache**  48M  Age = 32.81±5.72y Age range = 18 – 50y  Time since injury not disclosed | - 3T - TR = 2000ms - 5 - 6min - Eyes open/closed not reported | N/A | N/A | Trilinear/sinc-interpolation for 3 D motion correction. | N/A | ICA (Brain Voyager), Volume of interest (VOI). |
| Fang et al., 2021 | N/A | - 180 volumes - Eyes open/closed not reported | No patients excluded. | N/A | Images > 1.5mm translation and 1.5 degrees rotation excluded. | N/A | ROI based FC. |
| Fan et al., 2021 | **31 acute mTBI**  18M/13F  Age = 29.3±5.2y  Time since injury = 2.1±0.8d | - 3T - TR = 2000ms - 3 x 3 x 3mm - 240 volumes - Eyes closed | No subjects excluded. | N/A | Motion correction (SPM8).  Nuisance regression (head movement, CSF, WM). | N/A | Dynamic FC, sparse connectivity components (linear SVM). |
| ◊Di Battista et al., 2018 | **28 acute + chronic sports concussion (recently concussed + history of concussion)**  13M/15F  **16 acute sports concussion**  7M/9F  Age range= 18.0 – 19.75y  Time since injury = 1 – 7d)  **12 chronic sports concussion**  6M/6F  Age range = 20.5 – 22.0y  Time since injury = > 6mo | - 3T - TR = 2000ms - 3.125 x 3.125 x 4.0mm - 195 volumes - Eyes closed | N/A | N/A | Removal of outlier volumes (SPIKECOR).  Nuisance regression (motion parameters). | N/A | Global neural connectivity (Gconn). |
| DeSimone et al., 2021 | **50 American football athletes without concussion**  50M  Age = 11.5±1.2y Age range = 8 – 14y  No previous injuries (concussion) | - 3T - TR = 2000ms - 3.5 x 3.5 x 3.5mm - Eyes closed   2 scanning timepoints: pre and post football season. | N/A | N/A | Nuisance regression (consecutive functional volumes >0.5 mm in relative motion and time points with >10% of total voxels identified as signal outliers). | Age, BMI, time between pre- and post-season scans. | FC (whole brain and ROI). |
| Czerniak et al., 2015 | **9 sports concussion (student athletes)**  7M/2F  Age = 20.3±0.4y  Time since injury = 112d (range = 23 – 185d) | - 3T - TR = 2540ms - 6-7min - Eyes open fixated on cross | N/A | N/A | ICA nuisance regression (physiological noise and head motion). | Sex. | Hypothesis-driven seed-based approach (seed-to-ROI). |
| ◊Churchill et al., 2017 | 26 sports concussion  12M/14F  Age = 20y  Age range = 17 – 24y  Time since injury = 1 – 7d | - 3T - TR = 2000ms - 3.125 x 3.125 x 4.5mm - 194 volumes - Eyes closed | N/A | N/A | Removal of outlier scan volumes.  Nuisance regression (motion parameters, linear-quadratic trends, WM). | Acute symptoms, prior concussions, days post injury. | Global functional connectivity (Gconn). |
