# Supplementary material for: ENIGMA’s simple seven: Recommendations to enhance the reproducibility of resting-state fMRI in traumatic brain injury
Source: Neuroimage Clin. 2024 Mar 5;42:103585. doi: 10.1016/j.nicl.2024.103585 (PMC10982609; doi:10.1016/j.nicl.2024.103585)
Supplement: Supplemental Table References [file mmc2.docx]

**References Supplemental Table**

Abbas, K., Shenk, T.E., Poole, V.N., Breedlove, E.L., Leverenz, L.J., Nauman, E.A., Talavage, T.M., Robinson, M., E., 2015. Alteration of default mode network in high school football athletes due to repetitive subconcussive mild traumatic brain injury: a resting-state functional magnetic resonance imaging study. Brain Connect. 5 (2), 91–101. https://doi.org/10.1089/brain.2014.0279.

Amir, J., Nair, J.K.R., Del Carpio-O’Donovan, R., Ptito, A., Chen, J.K., Chankowsky, J., Tinawi, S., Lunkova, E., Saluja, R.S., 2021. Atypical resting state functional connectivity in mild traumatic brain injury. Brain Behav. 11 (8), e2261.

Amoroso, T., Iverson, K., 2017. Acknowledging the risk for traumatic brain injury in women veterans. J. Nerv. Ment. Dis. 205 (4), 318–323. https://doi.org/10.1097/ NMD.0000000000000621.

Astafiev, S.V., Zinn, K.L., Shulman, G.L., Corbetta, M., 2016. Exploring the physiological correlates of chronic mild traumatic brain injury symptoms. NeuroImage. Clin. 11, 10–19. https://doi.org/10.1016/j.nicl.2016.01.004.

Banks, S.D., Coronado, R.A., Clemons, L.R., Abraham, C.M., Pruthi, S., Conrad, B.N., Morgan, V.L., Guillamondegui, O.D., Archer, K.R., 2016. Thalamic functional connectivity in mild traumatic brain injury: longitudinal associations with patient- reported outcomes and neuropsychological tests. Arch. Phys. Med. Rehabil. 97 (8), 1254–1261. https://doi.org/10.1016/j.apmr.2016.03.013.

Bender Pape, T., Herrold, A. A., Siddiqi, S. H., Livengood, S. L., Bender Pape, T. L., Higgins, J. P.,... & Raij, T. (2020). Customizing TMS applications in traumatic brain injury using neuroimaging. *Journal of Head Trauma Rehabilitation*, *35*(6), 401-411. doi: 10.1097/HTR.0000000000000627.

Bernier, R.A., Roy, A., Venkatesan, U.M., Grossner, E.C., Brenner, E.K., Hillary, F.G., 2017. Dedifferentiation does not account for hyperconnectivity after traumatic brain injury. Front. Neurol. 8, 297. https://doi.org/10.3389/fneur.2017.00297.

Bittencourt, M., van der Horn, H.J., Balart-Sánchez, S.A., Marsman, J.C., van der Naalt, J., Maurits, N.M., 2022. Effects of mild traumatic brain injury on resting state brain network connectivity in older adults. Brain Imaging Behav. 16 (4), 1863–1872. https://doi.org/10.1007/s11682-022-00662-5.

Bittencourt-Villalpando, M., van der Horn, H.J., Maurits, N.M., van der Naalt, J., 2021. Disentangling the effects of age and mild traumatic brain injury on brain network connectivity: a resting state fMRI study. Neuroimage: Clin. 29, 1–14. https://doi.org/ 10.1016/j.nicl.2020.102534.

Black, L., Zammitti, E., Hoffman, H., & Li, C. (2016). Parental report of significant head injuries in children aged 3–17 years: United States, NCHS Data Brief, no 302. Hyattsville, MD: National Center for Health Statistics. 2018.

Botchway, E., Kooper, C.C., Pouwels, P.J.W., Bruining, H., Engelen, M., Oosterlaan, J., Königs, M., 2022. Resting-state network organisation in children with traumatic brain injury. Cortex 154, 89–104. https://doi.org/10.1016/j.cortex.2022.05.014.

Centers for Disease Control and Prevention. National Center for Health Statistics: Mortality Data on CDC WONDER. Accessed April 2023, https://wonder.cdc.gov/ mcd.html.

Champagne, A.A., Coverdale, N.S., Nashed, J.Y., Fernandez-Ruiz, J., Cook, D.J., 2019. Resting CMRO2 fluctuations show persistent network hyper-connectivity following exposure to sub-concussive collisions. NeuroImage. Clinical 22, 101753. https:// doi.org/10.1016/j.nicl.2019.101753.

Champagne, A.A., Coverdale, N.S., Ross, A., Chen, Y., Murray, C.I., Dubowitz, D., Cook, D.J., 2020. Multi-modal normalization of resting-state using local physiology reduces changes in functional connectivity patterns observed in mTBI patients. NeuroImage. Clinical 26, 102204. https://doi.org/10.1016/j.nicl.2020.102204.

Champagne, A.A., Coverdale, N.S., Ross, A., Murray, C., Vallee, I., Cook, D.J., 2021. Characterizing changes in network connectivity following chronic head trauma in special forces military personnel: a combined resting-fMRI and DTI study. Brain Inj. 35 (7), 760–768. https://doi.org/10.1080/02699052.2021.1906951.

Chong, C.D., Wang, L., Wang, K., Traub, S., Li, J., 2019. Homotopic region connectivity during concussion recovery: a longitudinal fMRI study. PLoS One 14 (10), e0221892.

Chou, Y., Chang, C., Remedios, S.W., Butman, J.A., Chan, L., Pham, D.L., 2022. Automated classification of resting-state fMRI ICA components using a deep siamese network. Front. Neurosci. 16, 768634. https://doi.org/10.3389/fnins.2022.768634.

Churchill, N.W., Hutchison, M.G., Richards, D., Leung, G., Graham, S.J., Schweizer, T.A., 2017. The first week after concussion: blood flow, brain function and white matter microstructure. NeuroImage. Clinical 14, 480–489. https://doi.org/10.1016/ j.nicl.2017.02.015.

Churchill, N.W., Hutchison, M.G., Graham, S.J., Schweizer, T.A., 2018. Connectomic markers of symptom severity in sport-related concussion: whole-brain analysis of resting-state fMRI. NeuroImage. Clinical 18, 518–526. https://doi.org/10.1016/ j.nicl.2018.02.011.

Churchill, N.W., Hutchison, M.G., Graham, S.J., Schweizer, T.A., 2019. Mapping brain recovery after concussion: from acute injury to 1 year after medical clearance. Neurology 93 (21), e1980–e1992. https://doi.org/10.1212/ WNL.0000000000008523.

Churchill, N.W., Hutchison, M.G., Graham, S.J., Schweizer, T.A., 2020. Neurometabolites and sport-related concussion: from acute injury to one year after medical clearance. NeuroImage. Clinical 27, 102258. https://doi.org/10.1016/j.nicl.2020.102258.

Churchill, N.W., Hutchison, M.G., Graham, S.J., Schweizer, T.A., 2021a. Concussion risk and resilience: relationships with pre-injury salience network connectivity. J. Neurotrauma 38 (22), 3097–3106. https://doi.org/10.1089/neu.2021.0123.

Churchill, N.W., Hutchison, M.G., Graham, S.J., Schweizer, T.A., 2021b. Brain function associated with reaction time after sport-related concussion. Brain Imaging Behav. 15 (3), 1508–1517. https://doi.org/10.1007/s11682-020-00349-9.

Churchill, N.W., Hutchison, M.G., Graham, S.J., Schweizer, T.A., 2021c. Long-term changes in the small-world organization of brain networks after concussion. Sci. Rep. 11 (1), 6862. https://doi.org/10.1038/s41598-021-85811-4.

Costanzo, M.E., Chou, Y.Y., Leaman, S., Pham, D.L., Keyser, D., Nathan, D.E., Coughlin, M., Rapp, P., Roy, M.J., 2014. Connecting combat-related mild traumatic brain injury with posttraumatic stress disorder symptoms through brain imaging. Neurosci. Lett. 577, 11–15. https://doi.org/10.1016/j.neulet.2014.05.054.

Czerniak, S.M., Sikoglu, E.M., Liso Navarro, A.A., McCafferty, J., Eisenstock, J., Stevenson, J.H., King, J.A., Moore, C.M., 2015. A resting state functional magnetic resonance imaging study of concussion in collegiate athletes. Brain Imaging Behav. 9 (2), 323–332. https://doi.org/10.1007/s11682-014-9312-1.

De Domenico, M., Sasai, S., Arenas, A., 2016. Mapping multiplex hubs in human functional brain networks. Front. Neurosci. 10, 326. https://doi.org/10.3389/ fnins.2016.00326.

De Simoni, S., Jenkins, P.O., Bourke, N.J., Fleminger, J.J., Hellyer, P.J., Jolly, A.E., Patel, M.C., Cole, J.H., Leech, R., Sharp, D.J., 2018. Altered caudate connectivity is associated with executive dysfunction after traumatic brain injury. Brain J. Neurol. 141 (1), 148–164. https://doi.org/10.1093/brain/awx309.

Di Battista, A.P., Churchill, N., Schweizer, T.A., Rhind, S.G., Richards, D., Baker, A.J., Hutchison, M.G., 2018. Blood biomarkers are associated with brain function and blood flow following sport concussion. J. Neuroimmunol. 319, 1–8. https://doi.org/ 10.1016/j.jneuroim.2018.03.002.

Diez, I., Drijkoningen, D., Stramaglia, S., Bonifazi, P., Marinazzo, D., Gooijers, J., Swinnen, S.P., Cortes, J.M. 2017. Enhanced prefrontal functional-structural networks to support postural control deficits after traumatic brain injury in a pediatric population. Net. Neuro. (Cambridge, Mass.). 1(2), 116–142. https://doi.org/10.1162/NETN_a_00007

Dona, O., Noseworthy, M.D., DeMatteo, C., Connolly, J.F., 2017. Fractal analysis of brain blood oxygenation level dependent (BOLD) signals from children with mild traumatic brain injury (mTBI). PLoS One 12 (1), e0169647.

D’Souza, M.M., Kumar, M., Choudhary, A., Kaur, P., Kumar, P., Rana, P., Trivedi, R., Sekhri, T., Singh, A.K., 2020. Alterations of connectivity patterns in functional brain networks in patients with mild traumatic brain injury: a longitudinal resting-state functional magnetic resonance imaging study. Neuroradiol. J. 33 (2), 186–197. https://doi.org/10.1177/1971400920901706.

Dudley, J., Yuan, W., Diekfuss, J., Barber Foss, K.D., DiCesare, C.A., Altaye, M., Logan, K., Leach, J.L., Myer, G.D., 2020. Altered functional and structural connectomes in female high school soccer athletes after a season of head impact exposure and the effect of a novel collar. Brain Connect. 10 (6), 292–301. https://doi.org/10.1089/ brain.2019.0729.

Dumkrieger, G., Chong, C.D., Ross, K., Berisha, V., Schwedt, T.J., 2019. Static and dynamic functional connectivity differences between migraine and persistent post- traumatic headache: a resting-state magnetic resonance imaging study. Cephalalgia : an International Journal of Headache 39 (11), 1366–1381. https://doi.org/10.1177/ 0333102419847728.

Edlow, B.L., Giacino, J.T., Wu, O., 2013. Functional MRI and outcome in traumatic coma. Curr. Neurol. Neurosci. Rep. 13, 1–11. https://doi.org/10.1007/s11910-013-0375-y.

Gębska-Kośla, K., Głąbiński, A., Sabiniewicz, M., Wolak, T., Wachowski, M., Kruczykowska, L., Majos, A., 2020. The use of functional magnetic resonance imaging techniques in the evaluation of patients with disorders of consciousness: a case report. Pol. J. Radiol. 85, e118–e124. https://doi.org/10.5114/pjr.2020.93664.

Gilbert, N., Bernier, R. A., Calhoun, V. D., Brenner, E., Grossner, E., Rajtmajer, S. M., & Hillary, F. G. (2018). Diminished neural network dynamics after moderate and severe traumatic brain injury. *PloS One*, *13*(6), e0197419. https://doi.org/10.1371/ journal.pone.0197419.

Goh, S.M., Irimia, A., Torgerson, C.M., Tubi, M.A., Real, C.R., Hanley, D.F., Van Horn, J.D., 2015. Longitudinal quantification and visualization of intracerebral haemorrhage using multimodal magnetic resonance and diffusion tensor imaging. Brain Inj. 29 (4), 438–445. https://doi.org/10.3109/02699052.2014.989907.

Goswami, R., Dufort, P., Tartaglia, M.C., Green, R.E., Crawley, A., Tator, C.H., Wennberg, R., Mikulis, D.J., Keightley, M., Davis, K.D., 2016. Frontotemporal correlates of impulsivity and machine learning in retired professional athletes with a history of multiple concussions. Brain Struct. Funct. 221 (4), 1911–1925. https://doi.org/ 10.1007/s00429-015-1012-0.

Guo, C., Ferreira, D., Fink, K., Westman, E., Granberg, T., 2019a. Repeatability and reproducibility of FreeSurfer, FSL-SIENAX and SPM brain volumetric measurements and the effect of lesion filling in multiple sclerosis. Eur. Radiol. 29 (3), 1355–1364. https://doi.org/10.1007/s00330-018-5710-x.

Guo, H., Liu, R., Sun, Z., Liu, B., Xiang, Y., Mao, J., Li, G., Zhang, M., 2019b. Evaluation of prognosis in patients with severe traumatic brain injury using resting-state functional magnetic resonance imaging. World Neurosurg. 121, e630–e639. https://doi.org/ 10.1016/j.wneu.2018.09.178.

Han, K., Mac Donald, C.L., Johnson, A.M., Barnes, Y., Wierzechowski, L., Zonies, D., Brody, D.L., 2014. Disrupted modular organization of resting-state cortical functional connectivity in US military personnel following concussive ‘mild’blast-related traumatic brain injury. Neuroimage 84, 76–96. https://doi.org/10.1016/ j.neuroimage.2013.08.017.

Han, K., Davis, R.A., Chapman, S.B., Krawczyk, D.C., 2017. Strategy-based reasoning training modulates cortical thickness and resting-state functional connectivity in adults with chronic traumatic brain injury. Brain and Behavior 7 (5), e00687.

Han, K., Chapman, S.B., Krawczyk, D.C., 2018. Neuroplasticity of cognitive control networks following cognitive training for chronic traumatic brain injury. NeuroImage. Clinical 18, 262–278. https://doi.org/10.1016/j.nicl.2018.01.030.

Hogeveen, J., Aragon, D.F., Rogge-Obando, K., Campbell, R.A., Shuttleworth, C.W., Avila- Rieger, R.E., Yeo, R.A., Wilson, J.K., Fratzke, V., Brandt, E., Story-Remer, J., Gill, D., Mayer, A.R., Cavanagh, J.F., Quinn, D.K., 2021. Ventromedial prefrontal-anterior cingulate hyperconnectivity and resilience to apathy in traumatic brain injury. J. Neurotrauma 38 (16), 2264–2274. https://doi.org/10.1089/neu.2020.7363.

Iraji, A., Benson, R.R., Welch, R.D., O’Neil, B.J., Woodard, J.L., Ayaz, S.I., Kulek, A., Mika, V., Medado, P., Soltanian-Zadeh, H., Liu, T., Haacke, E.M., Kou, Z., 2015. Resting state functional connectivity in mild traumatic brain injury at the acute stage: independent component and seed-based analyses. J. Neurotrauma 32 (14), 1031–1045. https://doi.org/10.1089/neu.2014.3610.

Iraji, A., Chen, H., Wiseman, N., Welch, R.D., O’Neil, B.J., Haacke, E.M., Liu, T., Kou, Z., 2016. Compensation through functional hyperconnectivity: a longitudinal connectome assessment of mild traumatic brain injury. Neural Plast. 2016, 4072402. https://doi.org/10.1155/2016/4072402.

Irimia, A., Van Horn, J.D., 2015. Functional neuroimaging of traumatic brain injury: advances and clinical utility. Neuropsychiatr. Dis. Treat. 11, 2355–2365. https:// doi.org/10.2147/NDT.S79174.

Irimia, A., Van Horn, J.D., Vespa, P.M., 2018. Cerebral microhemorrhages due to traumatic brain injury and their effects on the aging human brain. Neurobiol. Aging 66, 158–164. https://doi.org/10.1016/j.neurobiolaging.2018.02.026.

Irimia, A., Maher, A.S., Chaudhari, N.N., Chowdhury, N.F., Jacobs, E.B., 2020. Alzheimer’ s Disease Neuroimaging Initiative, acute cognitive deficits after traumatic brain injury predict alzheimer’s disease-like degradation of the human default mode network. GeroScience 42 (5), 1411–1429. https://doi.org/10.1007/s11357-020-00245-6.

Iyer, K.K., Barlow, K.M., Brooks, B., Ofoghi, Z., Zalesky, A., Cocchi, L., 2019. Relating brain connectivity with persistent symptoms in pediatric concussion. Ann. Clin. Transl. Neurol. 6 (5), 954–961. https://doi.org/10.1002/acn3.764.

Jia, X., Chang, X., Bai, L., Wang, Y., Dong, D., Gan, S., Wang, S., Li, X., Yang, X., Sun, Y., Li, T., Xiong, F., Niu, X., Yan, H., 2021. A longitudinal study of white matter functional network in mild traumatic brain injury. J. Neurotrauma 38 (19), 2686–2697. https://doi.org/10.1089/neu.2021.0017.

Johnson, B., Zhang, K., Gay, M., Neuberger, T., Horovitz, S., Hallett, M., Sebastianelli, W., Slobounov, S., 2012a. Metabolic alterations in corpus callosum may compromise brain functional connectivity in MTBI patients: an 1H-MRS study. Neurosci. Lett. 509 (1), 5–8. https://doi.org/10.1016/j.neulet.2011.11.013.

Johnson, B., Zhang, K., Gay, M., Horovitz, S., Hallett, M., Sebastianelli, W., Slobounov, S., 2012b. Alteration of brain default network in subacute phase of injury in concussed individuals: resting-state fMRI study. Neuroimage 59 (1), 511–518. https://doi.org/ 10.1016/j.neuroimage.2011.07.081.

Johnson, B., Zhang, K., Hallett, M., Slobounov, S., 2015. Functional neuroimaging of acute oculomotor deficits in concussed athletes. Brain Imaging Behav. 9 (3), 564–573. https://doi.org/10.1007/s11682-014-9316-x.

Johnson, B., Dodd, A., Mayer, A.R., Hallett, M., Slobounov, S., 2020. Correction to: are there any differential responses to concussive injury in civilian versus athletic populations: a neuroimaging study. Brain Imaging Behav. 14 (1), 118. https:// doi.org/10.1007/s11682-018-0015-x.

Kaushal, M., España, L.Y., Nencka, A.S., Wang, Y., Nelson, L.D., McCrea, M.A., Meier, T.B., 2019. Resting-state functional connectivity after concussion is associated with clinical recovery. Hum. Brain Mapp. 40 (4), 1211–1220. https://doi.org/10.1002/ hbm.24440.

Kazazian, K., Norton, L., Gofton, T.E., Debicki, D., Owen, A.M., 2020. Cortical function in acute severe traumatic brain injury and at recovery: a longitudinal fMRI case study. Brain Sci. 10 (9), 604. https://doi.org/10.3390/brainsci10090604.

Killgore, W.D.S., Vanuk, J.R., Shane, B.R., Weber, M., Bajaj, S., 2020. A randomized, double-blind, placebo-controlled trial of blue wavelength light exposure on sleep and recovery of brain structure, function, and cognition following mild traumatic brain injury. Neurobiol. Dis. 134, 104679. https://doi.org/10.1016/j.nbd.2019.104679.

Kim, E., Seo, H.G., Seong, M.Y., Kang, M.G., Kim, H., Lee, M.Y., Yoo, R.E., Hwang, I., Choi, S.H., Oh, B.M., 2022. An exploratory study on functional connectivity after mild traumatic brain injury: preserved global but altered local organization. Brain and Behavior 12 (9), e2735.

Knutson, K.M., Gotts, S.J., Wassermann, E.M., Lewis, J.D., 2020. Testosterone and resting state connectivity of the parahippocampal gyrus in men with history of deployment- related mild traumatic brain injury. Mil. Med. 185 (9–10), e1750–e1758. https:// doi.org/10.1093/milmed/usaa142.

Kuceyeski, A.F., Jamison, K.W., Owen, J.P., Raj, A., Mukherjee, P., 2019. Longitudinal increases in structural connectome segregation and functional connectome integration are associated with better recovery after mild TBI. Hum. Brain Mapp. 40 (15), 4441–4456. https://doi.org/10.1002/hbm.24713.

Kuceyeski, A., Navi, B.B., Kamel, H., Raj, A., Relkin, N., Toglia, J., O’dell, M., 2016. Structural connectome disruption at baseline predicts 6-months post-stroke outcome. Hum. Brain Mapp. 37 (7), 2587–2601. https://doi.org/10.1002/hbm.23198.

Lemme, J., Holmes, S., Sibai, D., Mari, J., Simons, L.E., Burstein, R., Zurakowski, D., Lebel, A., O’Brien, M., Upadhyay, J., Borsook, D., 2021. Altered brain network connectivity underlies persistent post-traumatic headache following mild traumatic brain injury in youth. J. Neurotrauma 38 (12), 1632–1641. https://doi.org/10.1089/ neu.2020.7189.

Leung, A., Shukla, S., Yang, E., Canlas, B., Kadokana, M., Heald, J., Davani, A., Song, D., Lin, L., Polston, G., Tsai, A., Lee, R., 2016. Diminished supraspinal pain modulation in patients with mild traumatic brain injury. Mol. Pain 12. https://doi.org/10.1177/ 1744806916662661.

Lewis, J.D., Knutson, K.M., Gotts, S.J., Tierney, M., Ramage, A., Tate, D.F., Clauw, D., Williams, D.A., Robin, D.A., Wassermann, E.M., 2021. Resting-state correlations of fatigue following military deployment. J. Neuropsychiatry Clin. Neurosci. 33 (4), 337–341. https://doi.org/10.1176/appi.neuropsych.20100255.

Li, F., Lu, L., Chen, H., Wang, P., Chen, Y.C., Zhang, H., Yin, X., 2019. Disrupted brain functional hub and causal connectivity in acute mild traumatic brain injury. Aging 11 (22), 10684–10696. https://doi.org/10.18632/aging.102484.

Li, F., Lu, L., Chen, H., Wang, P., Zhang, H., Chen, Y.C., Yin, X., 2020a. Neuroanatomical and functional alterations of insula in mild traumatic brain injury patients at the acute stage. Brain Imaging Behav. 14 (3), 907–916. https://doi.org/10.1007/s11682-019- 00053-3.

Li, F., Lu, L., Shang, S., Hu, L., Chen, H., Wang, P., Zhang, H., Chen, Y.C., Yin, X., 2020b. Disrupted functional network connectivity predicts cognitive impairment after acute mild traumatic brain injury. CNS Neurosci. Ther. 26 (10), 1083–1091. https:// doi.org/10.1111/cns.13430.

Lu, L., Li, F., Ma, Y., Chen, H., Wang, P., Peng, M., Chen, Y.C., Yin, X., 2019. Functional connectivity disruption of the substantia nigra associated with cognitive impairment in acute mild traumatic brain injury. Eur. J. Radiol. 114, 69–75. https://doi.org/ 10.1016/j.ejrad.2019.03.002.

Lu, L., Li, F., Chen, H., Wang, P., Zhang, H., Chen, Y.C., Yin, X., 2020a. Functional connectivity dysfunction of insular subdivisions in cognitive impairment after acute mild traumatic brain injury. Brain Imaging Behav. 14 (3), 941–948. https://doi.org/ 10.1007/s11682-020-00288-5.

Lu, L., Li, F., Wang, P., Chen, H., Chen, Y.C., Yin, X., 2020b. Altered hypothalamic functional connectivity in post-traumatic headache after mild traumatic brain injury. J. Headache Pain 21 (1), 93. https://doi.org/10.1186/s10194-020-01164-9.

Luo, X., Lin, D., Xia, S., Wang, D., Weng, X., Huang, W., Ye, H., 2021. Machine learning classification of mild traumatic brain injury using whole-brain functional activity: a radiomics analysis. Dis. Markers 2021, 3015238. https://doi.org/10.1155/2021/ 3015238.

Manning, K.Y., Schranz, A., Bartha, R., Dekaban, G.A., Barreira, C., Brown, A., Fischer, L., Asem, K., Doherty, T.J., Fraser, D.D., Holmes, J., Menon, R.S., 2017. Multiparametric MRI changes persist beyond recovery in concussed adolescent hockey players. Neurology 89 (21), 2157–2166. https://doi.org/10.1212/WNL.0000000000004669.

Markiewicz, Christopher J., De La Vega, Alejandro, Wagner, Adina, Halchenko, Yaroslav O., Finc, Karolina, Ciric, Rastko, Goncalves, Mathias, Nielson, Dylan M., Kent, James D., Lee, John A., Poldrack, Russell A., & Gorgolewski, Krzysztof J. (2021). poldracklab/fitlins: v0.9.2 (0.9.2). Zenodo. https://doi.org/10.5281/ zenodo.5120201.

Mayer, A.R., Ling, J.M., Allen, E.A., Klimaj, S.D., Yeo, R.A., Hanlon, F.M., 2015. Static and dynamic intrinsic connectivity following mild traumatic brain injury. J. Neurotrauma 32 (14), 1046–1055. https://doi.org/10.1089/neu.2014.3542.

McCuddy, W.T., España, L.Y., Nelson, L.D., Birn, R.M., Mayer, A.R., Meier, T.B., 2018. Association of acute depressive symptoms and functional connectivity of emotional processing regions following sport-related concussion. NeuroImage. Clinical 19, 434–442. https://doi.org/10.1016/j.nicl.2018.05.011.

Meier, T.B., Lancaster, M.A., Mayer, A.R., Teague, T.K., Savitz, J., 2017. Abnormalities in functional connectivity in collegiate football athletes with and without a concussion history: implications and role of neuroactive kynurenine pathway metabolites. J. Neurotrauma 34 (4), 824–837. https://doi.org/10.1089/neu.2016.4599.

Meier, T.B., Giraldo-Chica, M., Espana, L.Y., Mayer, A.R., Harezlak, J., Nencka, A.S., McCrea, M.A., 2020a. Resting-state fMRI metrics in acute sport-related concussion and their association with clinical recovery: a study from the NCAA-DOD CARE consortium. J. Neurotrauma 37 (1), 152–162. https://doi.org/10.1089/ neu.2019.6471.

Meier, T.B., Giraldo-Chica, M., España, L.Y., Mayer, A.R., Harezlak, J., Nencka, A.S., Wang, Y., Koch, K.M., Wu, Y.C., Saykin, A.J., Giza, C.C., Goldman, J., DiFiori, J.P., Guskiewicz, K.M., Mihalik, J.P., Brooks, A., Broglio, S.P., McAllister, T., McCrea, M.A., 2020b. Resting-state fMRI metrics in acute sport-related concussion and their association with clinical recovery: a study from the NCAA-DOD CARE consortium. J. Neurotrauma 37 (1), 152–162. https://doi.org/10.1089/neu.2019.6471.

Messé, A., Caplain, S., Pélégrini-Issac, M., Blancho, S., Lévy, R., Aghakhani, N., Montreuil, M., Benali, H., Lehéricy, S., 2013. Specific and evolving resting-state network alterations in post-concussion syndrome following mild traumatic brain injury. PLoS One 8 (6), e65470.

Militana, A.R., Donahue, M.J., Sills, A.K., Solomon, G.S., Gregory, A.J., Strother, M.K., Morgan, V.L., 2016. Alterations in default-mode network connectivity may be influenced by cerebrovascular changes within 1 week of sports related concussion in college varsity athletes: a pilot study. Brain Imaging Behav. 10 (2), 559–568. https:// doi.org/10.1007/s11682-015-9407-3.

Monroe, D.C., Blumenfeld, R.S., Keator, D.B., Solodkin, A., Small, S.L., 2020. One season of head-to-ball impact exposure alters functional connectivity in a central autonomic network. Neuroimage 223, 117306. https://doi.org/10.1016/ j.neuroimage.2020.117306.

Muller, A.M., Virji-Babul, N., 2018. Stuck in a state of inattention? functional hyperconnectivity as an indicator of disturbed intrinsic brain dynamics in adolescents with concussion: a pilot study. ASN Neuro 10. https://doi.org/10.1177/ 1759091417753802.

Murdaugh, D.L., King, T.Z., Sun, B., Jones, R.A., Ono, K.E., Reisner, A., Burns, T.G., 2018. Longitudinal changes in resting state connectivity and white matter integrity in adolescents with sports-related concussion. Journal of the International Neuropsychological Society: JINS 24 (8), 781–792. https://doi.org/10.1017/ S1355617718000413.

Nakamura, T., Hillary, F.G., Biswal, B.B., 2009. Resting network plasticity following brain injury. PLoS One 4 (12). https://doi.org/10.1371/journal.pone.0008220.

Nathan, D.E., Wang, B.Q., Wolfowitz, R.D., Liu, W., Yeh, P.H., Graner, J.L., Harper, J., Pan, H., Oakes, T.R., Riedy, G., 2012. Examining intrinsic thalamic resting state networks using graph theory analysis: implications for mTBI detection. annual international conference of the IEEE engineering in medicine and biology society. IEEE engineering in medicine and biology society. Annual International Conference 2012, 5445–5448. https://doi.org/10.1109/EMBC.2012.6347226.

Nathan, D.E., Oakes, T.R., Yeh, P.H., French, L.M., Harper, J.F., Liu, W., Riedy, G., 2015. Exploring variations in functional connectivity of the resting state default mode network in mild traumatic brain injury. Brain Connect. 5 (2), 102–114. https:// doi.org/10.1089/brain.2014.0273.

Nathan, D.E., Bellgowan, J.F., Oakes, T.R., French, L.M., Nadar, S.R., Sham, E.B., Liu, W., Riedy, G., 2016. Assessing quantitative changes in intrinsic thalamic networks in blast and nonblast mild traumatic brain injury: implications for mechanisms of injury. Brain Connect. 6 (5), 389–402. https://doi.org/10.1089/brain.2015.0403.

Nathan, D.E., Bellgowan, J.A.F., French, L.M., Wolf, J., Oakes, T.R., Mielke, J., Sham, E.B., Liu, W., Riedy, G., 2017. Assessing the impact of post-traumatic stress symptoms on the resting-state default mode network in a military chronic mild traumatic brain injury sample. Brain Connect. 7 (4), 236–249. https://doi.org/10.1089/ brain.2016.0433.

Newsome, M.R., Mayer, A.R., Lin, X., Troyanskaya, M., Jackson, G.R., Scheibel, R.S., Levin, H.S., 2016. Chronic effects of blast-related TBI on subcortical functional connectivity in veterans. J. Int. Neuropsychol. Soc. 22 (6), 631–642. https://doi.org/ 10.1017/S1355617716000448.

Newsome, M.R., Wilde, E.A., Bigler, E.D., Liu, Q., Mayer, A.R., Taylor, B.A., Steinberg, J.L., Tate, D.F., Abildskov, T.J., Scheibel, R.S., Walker, W.C., Levin, H.S., 2018. Functional brain connectivity and cortical thickness in relation to chronic pain in post-911 veterans and service members with mTBI. Brain Inj. 32 (10), 1236–1244. https://doi.org/10.1080/02699052.2018.1494853.

Niu, X., Bai, L., Sun, Y., Wang, S., Cao, J., Sun, C., Wang, Z., Xu, H., Gan, S., Fan, G., Huang, W., Gu, C., Yin, B., Bai, G., Xu, X., Zhang, M., 2019. Disruption of periaqueductal grey-default mode network functional connectivity predicts persistent post-traumatic headache in mild traumatic brain injury. J. Neurol. Neurosurg. Psychiatry 90 (3), 326–332. https://doi.org/10.1136/jnnp-2018-318886.

Nordin, L.E., Möller, M.C., Julin, P., Bartfai, A., Hashim, F., Li, T.Q., 2016. Post mTBI fatigue is associated with abnormal brain functional connectivity. Sci. Rep. 6, 21183. https://doi.org/10.1038/srep21183.

Orr, C.A., Albaugh, M.D., Watts, R., Garavan, H., Andrews, T., Nickerson, J.P., Gonyea, J., Hipko, S., Zweber, C., Logan, K., Hudziak, J.J., 2016. Neuroimaging biomarkers of a history of concussion observed in asymptomatic young athletes. J. Neurotrauma 33 (9), 803–810. https://doi.org/10.1089/neu.2014.3721.

Pagulayan, K.F., Petrie, E.C., Cook, D.G., Hendrickson, R.C., Rau, H., Reilly, M., Mayer, C., Meabon, J.S., Raskind, M.A., Peskind, E.R., Kleinhans, N., 2020. Effect of blast-related mTBI on the working memory system: a resting state fMRI study. Brain Imaging Behav. 14 (4), 949–960. https://doi.org/10.1007/s11682-018-9987-9.

Palacios, E.M., Yuh, E.L., Chang, Y.S., Yue, J.K., Schnyer, D.M., Okonkwo, D.O., Valadka, A.B., Gordon, W.A., Maas, A.I.R., Vassar, M., Manley, G.T., Mukherjee, P., 2017. Resting-state functional connectivity alterations associated with six-month outcomes in mild traumatic brain injury. J. Neurotrauma 34 (8), 1546–1557. https://doi.org/ 10.1089/neu.2016.4752.

Peters, M.E., Rahman, S., Coughlin, J.M., Pomper, M.G., Sair, H.I., 2020. Characterizing the link between glial activation and changed functional connectivity in National Football League Players Using Multimodal Neuroimaging. J. Neuropsychiatry Clin. Neurosci. 32 (2), 191–195. https://doi.org/10.1176/appi.neuropsych.18110274.

Philippi, C.L., Velez, C.S., Wade, B.S.C., Drennon, A.M., Cooper, D.B., Kennedy, J.E., Bowles, A.O., Lewis, J.D., Reid, M.W., York, G.E., Newsome, M.R., Wilde, E.A., Tate, D.F., 2021. Distinct patterns of resting-state connectivity in U.S. service members with mild traumatic brain injury versus posttraumatic stress disorder. Brain Imaging Behav. 15 (5), 2616–2626. https://doi.org/10.1007/s11682-021-00464-1.

Preti, M.G., Bolton, T.A., Van, D., De, Ville, 2017. The dynamic functional connectome: state-of-the-art and perspectives. Neuroimage 160. https://doi.org/10.1016/ j.neuroimage.2016.12.061.

Priestley, D.R., Staph, J., Koneru, S.D., Rajtmajer, S.M., Cwiek, A., Vervoordt, S., Hillary, F.G., 2023. Establishing ground truth in the traumatic brain injury literature: if replication is the answer, then what are the questions?. brain. Communications 5 (1), fcac322. <https://doi.org/10.1093/braincomms/fcac322>.

Puig, J., Ellis, M.J., Kornelsen, J., Figley, T.D., Figley, C.R., Daunis-i-Estadella, P., Essig, M., 2020. Magnetic resonance imaging biomarkers of brain connectivity in predicting outcome after mild traumatic brain injury: a systematic review. J. Neurotrauma 37 (16), 1761–1776. https://doi.org/10.1089/neu.2019.6623.

Rajesh, A., Cooke, G.E., Monti, J.M., Jahn, A., Daugherty, A.M., Cohen, N.J., Kramer, A.F., 2017. Differences in brain architecture in remote mild traumatic brain injury. J. Neurotrauma 34 (23), 3280–3287. https://doi.org/10.1089/neu.2017.5047.

Rangaprakash, D., Dretsch, M.N., Yan, W., Katz, J.S., Denney, Jr, T.S., Deshpande, G., 2017b. Hemodynamic variability in soldiers with trauma: implications for functional MRI connectivity studies. NeuroImage. Clinical 16, 409–417. https://doi.org/ 10.1016/j.nicl.2017.07.016.

Rigon, A., Duff, M.C., McAuley, E., Kramer, A.F., Voss, M.W., 2016. Is traumatic brain injury associated with reduced inter-hemispheric functional connectivity? a study of large-scale resting state networks following traumatic brain injury. J. Neurotrauma 33 (11), 977–989. https://doi.org/10.1089/neu.2014.3847.

Rigon, A., Voss, M.W., Turkstra, L.S., Mutlu, B., Duff, M.C., 2019. Functional neural correlates of facial affect recognition impairment following TBI. Brain Imaging Behav. 13 (2), 526–540. https://doi.org/10.1007/s11682-018-9889-x.

Robinson, M.E., Lindemer, E.R., Fonda, J.R., Milberg, W.P., McGlinchey, R.E., Salat, D.H., 2015. Close-range blast exposure is associated with altered functional connectivity in veterans independent of concussion symptoms at time of exposure. Hum. Brain Mapp. 36 (3), 911–922. https://doi.org/10.1002/hbm.22675.

Robinson, M.E., Clark, D.C., Milberg, W.P., McGlinchey, R.E., Salat, D.H., 2017. Characterization of differences in functional connectivity associated with close-range blast exposure. J. Neurotrauma 34 (S1), S53–S61. https://doi.org/10.1089/ neu.2016.4709.

Rostowsky, K.A., Maher, A.S., Irimia, A., 2018. Macroscale white matter alterations due to traumatic cerebral microhemorrhages are revealed by diffusion tensor imaging. Front. Neurol. 9, 948. https://doi.org/10.3389/fneur.2018.00948.

Roy, A., Bernier, R.A., Wang, J., Benson, M., French, Jr, J.J., Good, D.C., Hillary, F.G., 2018. Correction: the evolution of cost-efficiency in neural networks during recovery from traumatic brain injury. PLoS One 13 (10), e0206005.

Shafi, R., Crawley, A.P., Tartaglia, M.C., Tator, C.H., Green, R.E., Mikulis, D.J., Colantonio, A., 2020. Sex-specific differences in resting-state functional connectivity of large-scale networks in postconcussion syndrome. Sci. Rep. 10 (1), 21982. https:// doi.org/10.1038/s41598-020-77137-4.

Shapiro, J.S., Takagi, M., Silk, T., Anderson, N., Clarke, C., Davis, G.A., Hearps, S.J.C., Ignjatovic, V., Rausa, V., Seal, M.L., Babl, F.E., Anderson, V., 2021. No evidence of a difference in susceptibility-weighted imaging lesion burden or functional network connectivity between children with typical and delayed recovery two weeks post- concussion. J. Neurotrauma 38 (17), 2384–2390. https://doi.org/10.1089/ neu.2021.0069.

Sharma, B., Obeid, J., DeMatteo, C., Noseworthy, M.D., Timmons, B.W., 2022. Exploring the relationship between resting-state intra-network connectivity and accelerometer- measured physical activity in pediatric concussion: a cohort study. Appl. Physiol. Nutr. Metab. 47 (10), 1014–1022. https://doi.org/10.1139/apnm-2022-0085.

Sheth, C., Rogowska, J., Legarreta, M., McGlade, E., Yurgelun-Todd, D., 2021. Functional connectivity of the anterior cingulate cortex in veterans with mild traumatic brain injury. Behav. Brain Res. 396, 112882. https://doi.org/10.1016/j.bbr.2020.112882.

Slobounov, S.M., Gay, M., Zhang, K., Johnson, B., Pennell, D., Sebastianelli, W., Horovitz, S., Hallett, M., 2011. Alteration of brain functional network at rest and in response to YMCA physical stress test in concussed athletes: RsFMRI study. Neuroimage 55 (4), 1716–1727. https://doi.org/10.1016/j.neuroimage.2011.01.024.

Slobounov, S.M., Walter, A., Breiter, H.C., Zhu, D.C., Bai, X., Bream, T., Seidenberg, P., Mao, X., Johnson, B., Talavage, T.M., 2017. The effect of repetitive subconcussive collisions on brain integrity in collegiate football players over a single football season: a multi-modal neuroimaging study. NeuroImage. Clinical 14, 708–718. https:// doi.org/10.1016/j.nicl.2017.03.006.

Smith, J.L., Trofimova, A., Ahluwalia, V., Casado Garrido, J.J., Hurtado, J., Frank, R., Hodge, A., Gore, R.K., Allen, J.W., 2022. The “vestibular neuromatrix”: a proposed, expanded vestibular network from graph theory in post-concussive vestibular dysfunction. Hum. Brain Mapp. 43 (5), 1501–1518. https://doi.org/10.1002/ hbm.25737.

Sours, C., Zhuo, J., Janowich, J., Aarabi, B., Shanmuganathan, K., Gullapalli, R.P., 2013. Default mode network interference in mild traumatic brain injury - a pilot resting state study. Brain Res. 1537, 201–215. https://doi.org/10.1016/ j.brainres.2013.08.034.

Sours, C., Chen, H., Roys, S., Zhuo, J., Varshney, A., Gullapalli, R.P., 2015a. Investigation of multiple frequency ranges using discrete wavelet decomposition of resting-state functional connectivity in mild traumatic brain injury patients. Brain Connect. 5 (7), 442–450. https://doi.org/10.1089/brain.2014.0333.

Sours, C., George, E.O., Zhuo, J., Roys, S., Gullapalli, R.P., 2015b. Hyper-connectivity of the thalamus during early stages following mild traumatic brain injury. Brain Imaging Behav. 9 (3), 550–563. https://doi.org/10.1007/s11682-015-9424-2.

Sours, C., Rosenberg, J., Kane, R., Roys, S., Zhuo, J., Shanmuganathan, K., Gullapalli, R.P., 2015c. Associations between interhemispheric functional connectivity and the automated neuropsychological assessment metrics (ANAM) in civilian mild TBI. Brain Imaging Behav. 9 (2), 190–203. https://doi.org/10.1007/s11682-014-9295-y.

Sours, C., Zhuo, J., Roys, S., Shanmuganathan, K., Gullapalli, R.P., 2015d. Disruptions in resting state functional connectivity and cerebral blood flow in mild traumatic brain injury patients. PLoS One 10 (8), e0134019.

Spielberg, J.M., McGlinchey, R.E., Milberg, W.P., Salat, D.H., 2015. Brain network disturbance related to posttraumatic stress and traumatic brain injury in veterans. Biol. Psychiatry 78 (3), 210–216. https://doi.org/10.1016/j.biopsych.2015.02.013.

Stephens, J.A., Salorio, C.F., Barber, A.D., Risen, S.R., Mostofsky, S.H., Suskauer, S.J., 2018. Preliminary findings of altered functional connectivity of the default mode network linked to functional outcomes one year after pediatric traumatic brain injury. Dev. Neurorehabil. 21 (7), 423–430. https://doi.org/10.1080/ 17518423.2017.1338777.

Stephenson, D.D., Meier, T.B., Pabbathi Reddy, S., Robertson-Benta, C.R., Hergert, D.C., Dodd, A.B., Shaff, N.A., Ling, J.M., Oglesbee, S.J., Campbell, R.A., Phillips, J.P., Sapien, R.E., Mayer, A.R., 2020. Resting-state power and regional connectivity after pediatric mild traumatic brain injury. Journal of Magnetic Resonance Imaging : JMRI 52 (6), 1701–1713. https://doi.org/10.1002/jmri.27249.

Stevens, M.C., Lovejoy, D., Kim, J., Oakes, H., Kureshi, I., Witt, S.T., 2012. Multiple resting state network functional connectivity abnormalities in mild traumatic brain injury. Brain Imaging Behav. 6 (2), 293–318. https://doi.org/10.1007/s11682-012- 9157-4.

Straathof, M., Sinke, M.R., Dijkhuizen, R.M., Otte, W.M., 2019. A systematic review on the quantitative relationship between structural and functional network connectivity strength in mammalian brains. J. Cereb. Blood Flow Metab. 39 (2), 189–209. https://doi.org/10.1177/0271678X18809547.

Street, A., Gradus, J., Giasson, H., Vogt, D., Resick, P., 2013. Gender differences among veterans deployed in support of the wars in Afghanistan and Iraq. Journal of General International Medicine 28, 556–562.

Suárez, L.E., Markello, R.D., Betzel, R.F., Misic, B., 2020. Linking structure and function in macroscale brain networks. Trends Cogn. Sci. 24 (4), 302–315. https://doi.org/ 10.1016/j.tics.2020.01.008.

Sun, Y., Wang, S., Gan, S., Niu, X., Yin, B., Bai, G., Yang, X., Jia, X., Bai, L., Zhang, M., 2021. Serum neuron-specific enolase levels associated with connectivity alterations in anterior default mode network after mild traumatic brain injury. J. Neurotrauma 38 (11), 1495–1505. https://doi.org/10.1089/neu.2020.7372.

Threlkeld, Z.D., Bodien, Y.G., Rosenthal, E.S., Giacino, J.T., Nieto-Castanon, A., Wu, O., Whitfield-Gabrieli, S., Edlow, B.L., 2018. Functional networks reemerge during recovery of consciousness after acute severe traumatic brain injury. Cortex 106, 299–308. https://doi.org/10.1016/j.cortex.2018.05.004.

Toronov, V., Walker, S., Gupta, R., Choi, J.H., Gratton, E., Hueber, D., Webb, A., 2003. The roles of changes in deoxyhemoglobin concentration and regional cerebral blood volume in the fMRI BOLD signal. Neuroimage 19 (4), 1521–1531. https://doi.org/ 10.1016/S1053-8119(03)00152-6.

Trofimova, A., Smith, J.L., Ahluwalia, V., Hurtado, J., Gore, R.K., Allen, J.W., 2021. Alterations in resting-state functional brain connectivity and correlations with vestibular/ocular-motor screening measures in postconcussion vestibular dysfunction. Journal of Neuroimaging: Official Journal of the American Society of Neuroimaging 31 (2), 277–286. https://doi.org/10.1111/jon.12834.

Vakhtin, A.A., Calhoun, V.D., Jung, R.E., Prestopnik, J.L., Taylor, P.A., Ford, C.C., 2013. Changes in intrinsic functional brain networks following blast-induced mild traumatic brain injury. Brain Inj. 27 (11), 1304–1310. https://doi.org/10.3109/ 02699052.2013.823561.

Valera, E., Kucyi, A., 2017. Brain injury in women experiencing intimate partner-violence: neural mechanistic evidence of an “invisible” trauma. Brain Imaging Behav. 11 (6), 1664–1677. https://doi.org/10.1007/s11682-016-9643-1.

van der Horn, H.J., Liemburg, E.J., Scheenen, M.E., de Koning, M.E., Marsman, J.B., Spikman, J.M., van der Naalt, J., 2016a. Brain network dysregulation, emotion, and complaints after mild traumatic brain injury. Hum. Brain Mapp. 37 (4), 1645–1654. https://doi.org/10.1002/hbm.23126.

van der Horn, H.J., Liemburg, E.J., Scheenen, M.E., de Koning, M.E., Spikman, J.M., van der Naalt, J., 2016b. Post-concussive complaints after mild traumatic brain injury associated with altered brain networks during working memory performance. Brain Imaging Behav. 10 (4), 1243–1253. https://doi.org/10.1007/s11682-015-9489-y.

van der Horn, H.J., Kok, J.G., de Koning, M.E., Scheenen, M.E., Leemans, A., Spikman, J.M., van der Naalt, J., 2017a. Altered wiring of the human structural connectome in adults with mild traumatic brain injury. J. Neurotrauma 34 (5), 1035–1044. https:// doi.org/10.1089/neu.2016.4659.

van der Horn, H.J., Liemburg, E.J., Scheenen, M.E., de Koning, M.E., Spikman, J.M., van der Naalt, J., 2017b. Graph analysis of functional brain networks in patients with mild traumatic brain injury. PLoS One 12 (1), e0171031.

van der Horn, H.J., Vergara, V.M., Espinoza, F.A., Calhoun, V.D., Mayer, A.R., van der Naalt, J., 2020. Functional outcome is tied to dynamic brain states after mild to moderate traumatic brain injury. Hum. Brain Mapp. 41 (3), 617–631. https:// doi.org/10.1002/hbm.24827.

Vanhaudenhuyse, A., Noirhomme, Q., Tshibanda, L. J. F., Bruno, M. A., Boveroux, P., Schnakers, C.,... & Boly, M. (2010). Default network connectivity reflects the level of consciousness in non-communicative brain-damaged patients. *Brain*, *133*(1), 161- 171. https://doi.org/10.1093/brain/awp313.

Vaninetti, M., Lim, M., Khalaf, A., Metzger-Smith, V., Flowers, M., Kunnel, A., Yang, E., Song, D., Lin, L., Tsai, A., Lee, R., Golshan, S., Leung, A., 2021. fMRI findings in MTBI patients with headaches following rTMS. Sci. Rep. 11 (1), 9573. https://doi.org/ 10.1038/s41598-021-89118-2.

Vaughn, K.A., DeMaster, D., Kook, J.H., Vannucci, M., Ewing-Cobbs, L., 2022. Effective connectivity in the default mode network after paediatric traumatic brain injury. Eur. J. Neurosci. 55 (1), 318–336. https://doi.org/10.1111/ejn.15546.

Vergara, V.M., Mayer, A.R., Damaraju, E., Calhoun, V.D., 2017a. The effect of preprocessing in dynamic functional network connectivity used to classify mild traumatic brain injury. Brain and Behavior 7 (10), e00809.

Vergara, V.M., Mayer, A.R., Damaraju, E., Kiehl, K.A., Calhoun, V., 2017b. Detection of mild traumatic brain injury by machine learning classification using resting state functional network connectivity and fractional anisotropy. J. Neurotrauma 34 (5), 1045–1053. https://doi.org/10.1089/neu.2016.4526.

Vergara, V.M., Mayer, A.R., Kiehl, K.A., Calhoun, V.D., 2018. Dynamic functional network connectivity discriminates mild traumatic brain injury through machine learning. NeuroImage. Clinical 19, 30–37. https://doi.org/10.1016/j.nicl.2018.03.017.

Walton, S.R., Powell, J.R., Brett, B.L., Yin, W., Kerr, Z.Y., Liu, M., McCrea, M.A., Guskiewicz, K.M., Giovanello, K.S., 2022. Associations of lifetime concussion history and repetitive head impact exposure with resting-state functional connectivity in former collegiate american football players: an NCAA 15-year follow-up study. PLoS One 17 (9), e0273918.

Wang, S., Hu, L., Cao, J., Huang, W., Sun, C., Zheng, D., Wang, Z., Gan, S., Niu, X., Gu, C., Bai, G., Ye, L., Zhang, D., Zhang, N., Yin, B., Zhang, M., Bai, L., 2018. Sex differences in abnormal intrinsic functional connectivity after acute mild traumatic brain injury. Front. Neural Circuits 12, 107. https://doi.org/10.3389/fncir.2018.00107.

Wang, S., Gan, S., Yang, X., Li, T., Xiong, F., Jia, X., Sun, Y., Liu, J., Zhang, M., Bai, L., 2021a. Decoupling of structural and functional connectivity in hubs and cognitive impairment after mild traumatic brain injury. Brain Connect. 11 (9), 745–758. https://doi.org/10.1089/brain.2020.0852.

Wang, Z., Zhang, M., Sun, C., Wang, S., Cao, J., Wang, K.K.W., Gan, S., Huang, W., Niu, X., Zhu, Y., Sun, Y., Bai, L., 2021b. Single mild traumatic brain injury deteriorates progressive interhemispheric functional and structural connectivity. J. Neurotrauma 38 (4), 464–473. https://doi.org/10.1089/neu.2018.6196.

Whitfield-Gabrieli, S., Nieto-Castanon, A., 2012. Conn: a functional connectivity toolbox for correlated and anticorrelated brain networks. Brain Connect. 2 (3), 125–141. https://doi.org/10.1089/brain.2012.0073.

Wilde, E.A., Newsome, M.R., Ott, S.D., Hunter, J.V., Dash, P., Redell, J., Spruiell, M., Diaz, M., Chu, Z.D., Goodrich-Hunsaker, N., Petrie, J., Li, R., Levin, H., 2019. Persistent disruption of brain connectivity after sports-related concussion in a female athlete. J. Neurotrauma 36 (22), 3164–3171. https://doi.org/10.1089/neu.2019.6377.

Woytowicz, E.J., Sours, C., Gullapalli, R.P., Rosenberg, J., Westlake, K.P., 2018. Modulation of working memory load distinguishes individuals with and without balance impairments following mild traumatic brain injury. Brain Inj. 32 (2), 191–199. https://doi.org/10.1080/02699052.2017.1403045.

Wright, T., Urban, R., Durham, W., Dillon, E.L., Randolph, K.M., Danesi, C., Gilkison, C., Karmonik, C., Zgaljardic, D.J., Masel, B., Bishop, J., Pyles, R., Seidler, R., Hierholzer, A.H., Sheffield-Moore, M., 2020. Growth hormone alters brain morphometry, connectivity, and behavior in subjects with fatigue after mild traumatic brain injury. J. Neurotrauma 37 (8), 1052–1066. https://doi.org/10.1089/neu.2019.6690.

Wu, X., Kirov, I.I., Gonen, O., Ge, Y., Grossman, R.I., Lui, Y.W., 2016. MR imaging applications in mild traumatic brain injury: an imaging update. Radiology 279 (3), 693–707. https://doi.org/10.1148/radiol.16142535.

Wu, T., Rifkin, J.A., Rayfield, A., Panzer, M.B., Meaney, D.F., 2022. An interdisciplinary computational model for predicting traumatic brain injury: linking biomechanics and functional neural networks. Neuroimage 251, 119002. https://doi.org/10.1016/ j.neuroimage.2022.119002.

Xiong, K.L., Zhang, J.N., Zhang, Y.L., Zhang, Y., Chen, H., Qiu, M.G., 2016. Brain functional connectivity and cognition in mild traumatic brain injury. Neuroradiology 58 (7), 733–739. https://doi.org/10.1007/s00234-016-1675-0.

Xu, C., Li, Q., Gao, Y., Huo, H., Zhang, W., 2022. Changes and influencing factors of stress disorder in patients with mild traumatic brain injury stress disorder. Biomed Res. Int. 2022, 9082946. https://doi.org/10.1155/2022/9082946.

Yan, Y., Song, J., Xu, G., Yao, S., Cao, C., Li, C., Peng, G., Du, H., 2017. Correlation between standardized assessment of concussion scores and small-world brain network in mild traumatic brain injury. Journal of Clinical Neuroscience: Official Journal of the Neurosurgical Society of Australasia 44, 114–121. https://doi.org/10.1016/ j.jocn.2017.05.010.

Ye, L., Zhang, D., Shao, M., Zhao, P., Yin, B., Zhuang, J., Wang, F., Yan, Z., Bai, G., 2019. Lower posttraumatic α-synuclein level associated with altered default mode network connectivity following acute mild traumatic brain injury. Front. Neural Circuits 13, 26. https://doi.org/10.3389/fncir.2019.00026.

Yeh, F.C., Irimia, A., de Almeida Bastos, D.C., Golby, A.J., 2021. Tractography methods and findings in brain tumors and traumatic brain injury. Neuroimage 245, 118651. https://doi.org/10.1016/j.neuroimage.2021.118651.

Yuan, W., Wade, S.L., Quatman-Yates, C., Hugentobler, J.A., Gubanich, P.J., Kurowski, B.G., 2017. Structural connectivity related to persistent symptoms after mild TBI in adolescents and response to aerobic training: preliminary investigation. J. Head Trauma Rehabil. 32 (6), 378–384. https://doi.org/10.1097/ HTR.0000000000000318.

Zhan, J., Gao, L., Zhou, F., Kuang, H., Zhao, J., Wang, S., He, L., Zeng, X., Gong, H., 2015. Decreased regional homogeneity in patients with acute mild traumatic brain injury: a resting-state fMRI study. J. Nerv. Ment. Dis. 203 (10), 786–791. https://doi.org/ 10.1097/NMD.0000000000000368.

Zhang, K., Johnson, B., Gay, M., Horovitz, S.G., Hallett, M., Sebastianelli, W., Slobounov, S., 2012. Default mode network in concussed individuals in response to the YMCA physical stress test. J. Neurotrauma 29 (5), 756–765. https://doi.org/10.1089/ neu.2011.2125.

Zhang, J., Zhang, E., Yuan, C., Zhang, H., Wang, X., Yan, F., Pei, Y., Li, Y., Wei, M., Yang, Z., Wang, X., Dong, L., 2022. Abnormal default mode network could be a potential prognostic marker in patients with disorders of consciousness. Clin. Neurol. Neurosurg. 218, 107294. https://doi.org/10.1016/j.clineuro.2022.107294.

Zhao, Y., Dong, Q., Chen, H., Iraji, A., Li, Y., Makkie, M., Kou, Z., Liu, T., 2017. Constructing fine-granularity functional brain network atlases via deep convolutional autoencoder. Med. Image Anal. 42, 200–211. https://doi.org/10.1016/ j.media.2017.08.005.

Zhou, Y., 2017a. Abnormal structural and functional hypothalamic connectivity in mild traumatic brain injury. Journal of Magnetic Resonance Imaging: JMRI 45 (4), 1105–1112. https://doi.org/10.1002/jmri.25413.

Zhou, Y., 2017b. Small world properties changes in mild traumatic brain injury. Journal of Magnetic Resonance Imaging: JMRI 46 (2), 518–527. https://doi.org/10.1002/ jmri.25548.

Zhou, Y., Lui, Y.W., Zuo, X.N., Milham, M.P., Reaume, J., Grossman, R.I., Ge, Y., 2014. Characterization of thalamo-cortical association using amplitude and connectivity of functional MRI in mild traumatic brain injury. Journal of Magnetic Resonance Imaging: JMRI 39 (6), 1558–1568. https://doi.org/10.1002/jmri.24310.

Zhu, D.C., Covassin, T., Nogle, S., Doyle, S., Russell, D., Pearson, R.L., Monroe, J., Liszewski, C.M., DeMarco, J.K., Kaufman, D.I., 2015. A potential biomarker in sports- related concussion: brain functional connectivity alteration of the default-mode network measured with longitudinal resting-state fMRI over thirty days. J. Neurotrauma 32 (5), 327–341. https://doi.org/10.1089/neu.2014.3413.
